# Does combining numerous data types in multi-omics data improve or hinder performance in survival prediction? Insights from a large-scale benchmark study

**DOI:** 10.1186/s12911-024-02642-9

**Published:** 2024-09-02

**Authors:** Yingxia Li, Tobias Herold, Ulrich Mansmann, Roman Hornung

**Affiliations:** 1 Institute for Medical Information Processing, Biometry and Epidemiology, LMU Munich, Marchioninistr. 15, 81377 Munich, Germany; 2 Laboratory for Leukemia Diagnostics, Department of Medicine III, LMU University Hospital, LMU Munich, Munich, Germany; 3 Munich Center for Machine Learning (MCML), Munich, Germany

**Keywords:** Multi-omics data, Prediction, TCGA, Benchmark, Cancer, Survival analysis

## Abstract

**Background:**

Predictive modeling based on multi-omics data, which incorporates several types of omics data for the same patients, has shown potential to outperform single-omics predictive modeling. Most research in this domain focuses on incorporating numerous data types, despite the complexity and cost of acquiring them. The prevailing assumption is that increasing the number of data types necessarily improves predictive performance. However, the integration of less informative or redundant data types could potentially hinder this performance. Therefore, identifying the most effective combinations of omics data types that enhance predictive performance is critical for cost-effective and accurate predictions.

**Methods:**

In this study, we systematically evaluated the predictive performance of all 31 possible combinations including at least one of five genomic data types (mRNA, miRNA, methylation, DNAseq, and copy number variation) using 14 cancer datasets with right-censored survival outcomes, publicly available from the TCGA database. We employed various prediction methods and up-weighted clinical data in every model to leverage their predictive importance. Harrell’s C-index and the integrated Brier Score were used as performance measures. To assess the robustness of our findings, we performed a bootstrap analysis at the level of the included datasets. Statistical testing was conducted for key results, limiting the number of tests to ensure a low risk of false positives.

**Results:**

Contrary to expectations, we found that using only mRNA data or a combination of mRNA and miRNA data was sufficient for most cancer types. For some cancer types, the additional inclusion of methylation data led to improved prediction results. Far from enhancing performance, the introduction of more data types most often resulted in a decline in performance, which varied between the two performance measures.

**Conclusions:**

Our findings challenge the prevailing notion that combining multiple omics data types in multi-omics survival prediction improves predictive performance. Thus, the widespread approach in multi-omics prediction of incorporating as many data types as possible should be reconsidered to avoid suboptimal prediction results and unnecessary expenditure.

**Supplementary Information:**

The online version contains supplementary material available at 10.1186/s12911-024-02642-9.

## Background

Cancer is a global public health problem due to its high morbidity and mortality rates [1]. It is associated with alterations in genes that control normal cell growth and differentiation. Thus, understanding and exploiting the molecular basis of cancer has many benefits, including the possibility of building prediction models [2, 3], discovering biomarkers [4], identifying abnormal pathways [5], and determining optimal treatment options.

Today, various types of omics data exist. These include genomic, epigenomic, transcriptomic, proteomic, and metabolomic data. Many of these data types are publicly available on The Cancer Genome Atlas (TCGA) [6]. In the following, the different types of molecular data are often referred to as “blocks”. Omics data have been used to develop predictive models for more than 20 years. These models traditionally used only one block, the mRNA block being likely the most commonly used. As a well-known example, mRNA data have often been found to be useful for predicting survival or response to therapy in cancer patients [7]. With the increasing availability of other types of blocks, the focus has shifted towards constructing predictive models based on multi-omics data, that is, several block types available for the same patients. Several analyses allow the interpretation that multi-omics data outperform single-omics data in predictive modeling [8–11]. For example, Li et al. [12] found that using multi-omics data delivered notably better results than using single-omics data in the prediction of the stage of lung adenocarcinoma.

The prevailing assumption in the field, as evidenced by the multi-omics literature, is that incorporating as many blocks as feasible optimizes predictive performance. However, recent findings have suggested that this strategy may inadvertently lead to suboptimal results if less informative or redundant blocks are included (see next paragraph for details). To our knowledge, a thorough examination of which blocks consistently improve the predictive performance and which blocks can and should, in general, be left out has not been conducted. Omitting certain data blocks could not only potentially enhance predictive performance but also lead to cost savings. In fact, it is quite costly and laborious to obtain different blocks for the same patient. The huge data volume of multi-omics data can also lead to long computation times and large consumptions of computational memory. Thus, ideally, the number of blocks should be small to reduce the costs and complexities involved in the clinical implementation and acquisition of patients’ molecular data. Moreover, given the costs and efforts needed to obtain multi-omics data, when including multiple blocks at the same time the sample sizes associated with these data can be expected to be small. Prediction models derived from small sample sizes are likely to be less reliable, especially when dealing with a large number of features.

Several studies have compared the predictive performance of different block combinations [2, 3, 13–17]; however [11], these studies tend to be limited in scope and yielded partially inconsistent results. They have almost exclusively either considered very few [2, 3, 15] or a limited number of block combinations [13, 14, 16]. Only one study [17] followed the comprehensive approach used in this paper, evaluating all possible combinations of the considered omics blocks, but it relied on a single dataset of limited size. Some studies used only a few datasets [13, 14] or a single prediction method [16]. In general, in these studies, the predictive performance tended to be better when using subsets of the available blocks compared to the entire set. All of these studies found mRNA data to be particularly effective for prediction, with individual studies also highlighting the predictive value of methylation [16], miRNA [14], copy-number variation (CNV) [14], and plasma protein data [17]. For a more detailed overview of these studies, we direct the interested reader to Additional file 1.

In the present study, we conducted a large-scale benchmark experiment using TCGA data to explore which combinations of blocks tend to provide the most accurate survival prediction results for various cancer types. We compared the predictive performance of all possible 31 combinations that contain at least one of five blocks (mRNA, miRNA, methylation, DNAseq, and CNV) across 14 cancer datasets with survival outcome, using five prediction methods, specifically machine learning and statistical approaches for survival outcomes. Clinical covariates were included in each combination and prioritized for four of the five prediction methods (see “Experimental settings” section for details).

In this work, we focus on the direct, sample-wise concatenation of the different omics data types, rather than integration through data transformations. As described by Picard et al. [18], there are a variety of integration strategies applicable to multi-omics data beyond direct concatenation. These include classical matrix factorization methods as well as more recent techniques using graph-based approaches or deep learning. Notably, recent developments tailored to multi-omics data include UNMF [19], which is based on nonnegative matrix factorization, and TransPro [20], which applies deep learning for the hierarchical integration of omics data types in accordance with the central dogma of molecular biology.

In the interest of developing general guidelines for omitting non-essential blocks, our initial analysis focuses on the average performance of the block combinations across all datasets. This step provides insight into which combinations are generally effective for most cancer types. Subsequently, we examine the performance of these combinations individually for each dataset. This detailed analysis aims to determine if specific cancer types require specific block combinations to achieve strong predictive performance. It is also important to clarify that our aim is not to make biological interpretations. The sole objective of our study is to provide guidance for designing (multi-)omics experiments to enhance predictive performance and reduce costs in the field by identifying which blocks can typically be excluded from multi-omics data-based prediction. In contrast, many studies primarily seek to disentangle the complexity of disease processes. In such cases, where the focus is on interpretation, the inclusion of multiple blocks is undoubtedly advantageous.

## Methods

### Aim of the benchmark study

The aim of this study is to determine the combinations of blocks that are most effective in survival prediction for different types of cancer.

### Datasets

The 14 included multi-omics datasets from TCGA were the same as those studied in [2], except that we included methylation data in addition. For each cancer type, there are five omics blocks and clinical covariates, that is, six groups of features. An overview of these 14 datasets is given in Table 1. We used the same clinical covariates as in [2], where covariates most commonly available across datasets were selected, as well as cancer-specific covariates identified through an informal literature review. In Additional file 1, we provide detailed information on which clinical covariates were used for which datasets. As outcome we used overall survival.

The 14 datasets are a subset of originally 26 available datasets. Datasets with missing omics blocks were excluded and those where less than 5% of patients had observed events, that is, uncensored survival times. Moreover, each dataset was subset to include no missing values in the clinical covariates. For further preprocessing details, refer to [2].

**Table 1:** Overview of the considered datasets. The third to the eighth column show the numbers of features in the respective feature blocks (clin: clinical covariates, cnv: CNV, mirna: miRNA, mut: DNAseq, met: methylation, rna: mRNA). The last four columns show, in this order, the total number of features (f), the numbers of observations (n), the numbers of observed events (n_e), and the proportions of observed events (r_e)

Dataset	Cancer	clin	cnv	mirna	mut	met	rna	f	*n*	n_e	r_e
BLCA	Bladder urothelial	5	57,964	825	18,577	382,711	23,081	483,166	382	103	0.27
BRCA	Breast invasive C.	8	57,964	835	17,975	21,919	22,694	121,398	735	72	0.10
COAD	Colon AC.	7	57,964	802	18,538	22,418	22,210	121,942	191	17	0.09
ESCA	Esophageal C.	6	57,964	763	12,628	383,295	25,494	480,153	106	37	0.35
HNSC	Head–neck squamous CC.	11	57,964	793	17,248	376,058	21,520	473,597	443	152	0.34
LGG	Low grade glioma	10	57,964	645	9235	373,499	22,297	463,653	419	77	0.18
LIHC	Liver hepatocellular C.	11	57,964	776	11,821	378,427	20,994	469,996	159	35	0.22
LUAD	Lung AC.	9	57,964	799	18,388	22,486	23,681	123,330	426	101	0.24
LUSC	Lung squamous CC.	9	57,964	895	18,500	21,364	23,524	122,259	418	132	0.32
PAAD	Pancreatic AC.	10	57,964	612	12,392	375,464	22,348	468,793	124	52	0.42
SARC	Sarcoma	11	57,964	778	10,001	378,139	22,842	469,738	126	38	0.30
SKCM	Skin cutaneous M.	9	57,964	1002	18,593	377,193	22,248	477,012	249	62	0.21
STAD	Stomach AC.	7	57,964	787	18,581	22,557	26,027	125,926	295	62	0.21
UCEC	Uterine corpus EC.	11	57,447	866	21,053	22,517	23,978	125,875	405	38	0.09

*Abbreviations* C. indicates carcinoma; AC., adenocarcinoma; CC., cell carcinoma; M., melanoma; EC., endometrial carcinoma

### Feature selection

The permutation-based variable importance measure of random survival forests (RF-VI) can be used to rank features in terms of their importance to prediction. It can be used in feature selection by retaining the best-ranking variables. In a previous work, we conducted a benchmark study of feature selection strategies for multi-omics data with binary outcomes, where we found that RF-VI is quite robust with respect to the number of features selected and is relatively fast [21]. Thus, for blocks with more than 2,500 variables, we used the RF-VI feature selection method to perform feature selection on the training datasets within (5-fold) cross-validation. Here, we selected the 2,500 features with the largest variable importance measure values from each of these blocks, where Harrell’s concordance index was used as performance measure in RF-VI. This was done for computational efficiency and because most variables do not carry information in the ultra-high-dimensional blocks. Because of the large numbers of features, for some blocks (particularly the methylation block), we used 10,000 trees per random survival forest instead of the 500 trees that are default in the R package ranger (version 0.14) used. Note that it is crucial to perform feature selection within cross-validation on the training datasets. Conducting feature selection on the entire dataset before cross-validation typically results in a substantial overestimation of the predictive performance [22, 23]. This overestimation occurs even when many features are selected [24], as in our study. The choice of 2,500 selected features was not based on any specific statistical criteria, such as predictive performance, which could be optimized through cross-validation-based tuning. Instead, this number was chosen to be sufficiently large to likely include most features of notable influence, while balancing the computational demands. The objective was not to exhaustively identify every influential feature while discarding all non-influential ones, which is the reason behind the absence of statistical testing in our feature selection process.

When more blocks are included, the total number of features available to the prediction models increases. This could potentially benefit combinations that include many blocks. To counter this effect, the same total number of selected features could be used for each model, irrespective of the number of blocks. However, this would be counterproductive, since in practice there are also more features are available in total when more blocks are used. As our aim is to provide recommendations that align with practical applications, our benchmark study mirrors the procedures typically followed in practice. Thus, in scenarios with larger numbers of blocks, the models use more features, which might give these models an advantage over those with fewer blocks, mirroring the real-world scenario.

### Survival prediction methods

We employed five distinct prediction methods that were most commonly used in previous benchmark studies on prediction using multi-omics data [2, 3, 13–16]. These five approaches include both prediction methods specifically designed for multi-omics data, as well as methods appropriate for high-dimensional data broadly. Unlike some earlier studies [15–17], we excluded deep learning approaches because they typically require Python, whereas our study was restricted to methods implemented in R. Additionally, Wissel et al. [15] reported that deep learning methods generally exhibit poorer calibration when applied to (multi-)omics data compared to statistical or classical machine learning methods. For an extensive review of deep learning methods for survival outcomes, refer to Wiegrebe et al. [25]. Bayesian approaches were also excluded. As noted by Zhao et al. [26], while Bayesian methods can readily quantify uncertainty in parameters and predictive outcomes and offer flexible modeling, they are computationally intensive for high-dimensional data; in their overview paper, Zhao et al. provide a detailed discussion of these methods, focused on Cox-based ones. Compared to the multitude of survival prediction methods available for omics data, those specifically tailored to multi-omics data are relatively limited.

Table 2 provides an overview of the five methods used in our study, including the R packages that implement them and the types of prediction outcomes used to measure their performance.

**Table 2:** Overview of the survival prediction methods used in the benchmark study

Method	*R* package (version)	Prediction types
Random survival forests (rsf)	ranger (0.13.1)	For C-index calculation: Sum of the values of the bootstrap ensemble cumulative hazard function [27] $\:{H}_{e}^{*}\left(t\right|{\varvec{x}}_{i})$ calculated at all unique death times. For integrated Brier score: Survival function estimated using $\:{\text{e}\text{x}\text{p}(-H}_{e}^{*}\left(t\right|{\varvec{x}}_{i}\left)\right)$
Block forests (bf)	blockForest (0.2.4)	See rsf above.
Lasso (lasso)	glmnet (4.1-3)	For C-index calculation: Linear predictor $\:{\varvec{x}}_{i}^{T}\widehat{\varvec{\beta\:}}$ For integrated Brier score: Survival function estimated as follows: $\:{\text{e}\text{x}\text{p}(-\widehat{{\Lambda\:}}}_{0}\left(t\right)\:\text{e}\text{x}\text{p}\left({\varvec{x}}_{i}^{T}\widehat{\varvec{\beta\:}}\right))$, where $\:{\widehat{{\Lambda\:}}}_{0}\left(t\right)$ is an estimate of the baseline cumulative hazard function obtained using the Efron estimator
IPF-LASSO (ipflasso)	ipflasso (1.1)	See lasso above.
Priority-Lasso (prioritylasso)	prioritylasso (0.2.5)	See lasso above.

#### Random survival forests

Random forests [28] are ensemble classifiers that use randomly selected training samples as well as repeatedly and randomly selected subsets of variables to produce multiple, heterogeneous decision trees. They have become popular due to their ability to capture complex patterns of dependencies between the outcome and the input features. However, they are not designed to take the multi-omics group structure into account. We used random survival forests (rsf) [27], a variant of random forests or survival outcomes. No hyperparameter tuning was performed for this method, and the default values available in the R package “ranger” (version 0.13.1) were used. For example, the parameter mtry was set to the rounded down square root of the number of features. This use of default hyperparameter values is supported by Probst et al. [29], who demonstrated in a study involving many datasets that the performance of random forests is only slightly affected by the choice of the hyperparameter values.

#### Block forests

The block forests (bf) algorithm [3] is a variant of random forests that modifies the split selection of random forests to incorporate the block structure of multi-omics data. This algorithm has a weight parameter for each block. These hyperparameters were tuned using an optimization procedure described in [3], which is performed by default in the R package “blockForest” (version 0.2.4), which implements the bf algorithm.

#### Lasso

The least absolute shrinkage and selection operator (Lasso) [30] is a penalized regression method that applies an L1 penalty to shrink coefficients of features without strong impact on the predictions to zero. When using multi-omics data to predict clinical outcomes, Lasso regression penalizes each feature equally across all blocks by using a single penalization parameter for the entire dataset. That is, like rsf, the method does not take the multi-omics group structure into account. As the Lasso was originally introduced only for predicting continuous outcomes, we used a version (lasso) for predicting survival prediction [31] based on the Cox model [32], referred to as Cox-Lasso in the following. The penalty parameter was tuned using 10-fold cross-validation with the function “cv.glmnet” from the R package “glmnet” (version 4.1.3).

#### IPF-LASSO

The IPF-LASSO [33] is an extension of the Lasso that takes the group structure into account by using different penalty parameter values for each block. Its version for survival outcomes is based on the Cox-Lasso. We used a variant of the IPF-LASSO, which performs an efficient two-step procedure to optimize the penalty parameter values (ipflasso) [34].

#### Priority-Lasso

The priority-Lasso (prioritylasso) [35], like the IPF-LASSO, is an extension of the Lasso. It is based on the principle of defining a priority order on the blocks of variables. Subsequently, prioritylasso successively fits Lasso regression models to the blocks in the order of their priority, where at each step, the resulting linear predictor is used as an offset for the Lasso model fit to the next block.

For the current study, however, we did not have any substantial domain knowledge needed for assigning the priority order to the blocks for the different cancer types. Therefore, we used the ranking of the penalty factor values determined in the first step of the ipflasso as a surrogate for knowledge-based prioritization, that is, the block with the smallest penalty factor was given the highest priority, the block with the second smallest penalty factor was given the second highest priority, and so on. In the case of survival outcomes, the priority-Lasso is based on Cox-Lasso models. The penalty parameters of the successively fitted Lasso models were optimized using 10-fold cross-validation with the function “cv.glmnet” from the R package “glmnet” (version 4.1.3).

### Experimental settings

Clinical covariates carry important predictive information and several studies have demonstrated that their inclusion improves predictive performance [2, 3]. It is important to up-weight or “prioritize” the clinical covariates over the omics blocks to exploit their predictive information [36, 37] because there are typically many more omics features than clinical covariates. Therefore, except for in the case of ipflasso, where this was not possible, we prioritized the clinical covariates for all prediction methods. For rsf, this was achieved by adding all clinical covariates to the randomly sampled covariates for each split in the trees constituting the rsf. For bf, similarly, the clinical block was always included in the blocks considered for splitting. For lasso, the coefficients of the clinical covariates were exempt from the L1 penalization-based shrinkage. Finally, for prioritylasso, the clinical block always had the highest priority and, as in the case of lasso, no shrinkage was performed for the clinical covariates.

For each dataset, we considered all 2^5^ – 1 = 31 possible combinations containing at least one of the omics blocks (the clinical covariates were always included) and compared the predictive performance achieved with the different combinations. We repeated the analysis for each of the five prediction methods considered.

The integrated Brier score (ibrier) [38] and Harrell’s concordance index (cindex) [39] were used to evaluate the predictive performance. The ibrier is a calibration measure that assesses how accurate the predicted survival functions are. It also measures discrimination and is a commonly used scoring rule.

In contrast, the cindex is a discrimination measure only. It assesses how well the prediction model can rank different patients according to their risk. Specifically, it estimates the probability that, when choosing two patients at random, the model assigns a higher risk to the patient with the shorter survival time. This measure depends on the type of risk measure used. Sonabend et al. [40] elaborate that an appropriate risk measure for the cindex is “expected mortality”, which is calculated by summing the predicted cumulative hazard function over all observed death times [27]. This approach does not necessitate assumptions about the model or the survival distribution beyond the observed time frame and offers clear interpretability: a higher value indicates a greater risk of death. As shown in Table 2, we use this measure for the random forest variants rsf and bf. For the Lasso variants, we use the linear predictor in the calculation of the cindex. However, it is apparent that this is equivalent to using the expected mortality since the formula for the individual patient hazard function in the Lasso variants mirrors that in the classical Cox model. In the latter, it is straightforward to see that the expected mortality monotonically increases with the linear predictor. We used the R packages “pec” (version 2022.03.06) and “survcomp” (version 1.44.1) for estimating the ibrier and cindex, respectively.

As an evaluation scheme, we used 5-fold cross-validation repeated five times, without stratification based on the censoring indicator, although ideally, this should have been done. There were no errors in applying the five prediction methods across any of the cross-validation iterations, resulting in no missing values in the performance measure values. The benchmark experiment was conducted using R version 4.1.2 [41]. All R code written to produce and evaluate our results is available on GitHub (https://github.com/YingxiaLi2023/multi-omics-data, commit hash: 5531dcea6f63a08fe9f1c02e53b7cc0666751227, accessed on August 12, 2024).

## Results

This section presents the full results of our benchmark study. Readers seeking a condensed overview are encouraged to skip to the “Discussion” section, where the main findings are reviewed and contextualized

### Ranking of the predictive information contained in all block combinations per prediction method

In this subsection, we initially present and discuss the results in a purely descriptive manner. We then present the results of a bootstrap analysis, aimed at evaluating the statistical significance of certain overall patterns in the results

For the sake of clarity, we present here only the results obtained for the ibrier with rsf [27], bf [3], and the ipflasso [33, 34]. The results obtained for the ibrier with the lasso [30] and the prioritylasso [35], as well as all results obtained for the cindex are shown in Additional file 1.

**Fig. 1:**
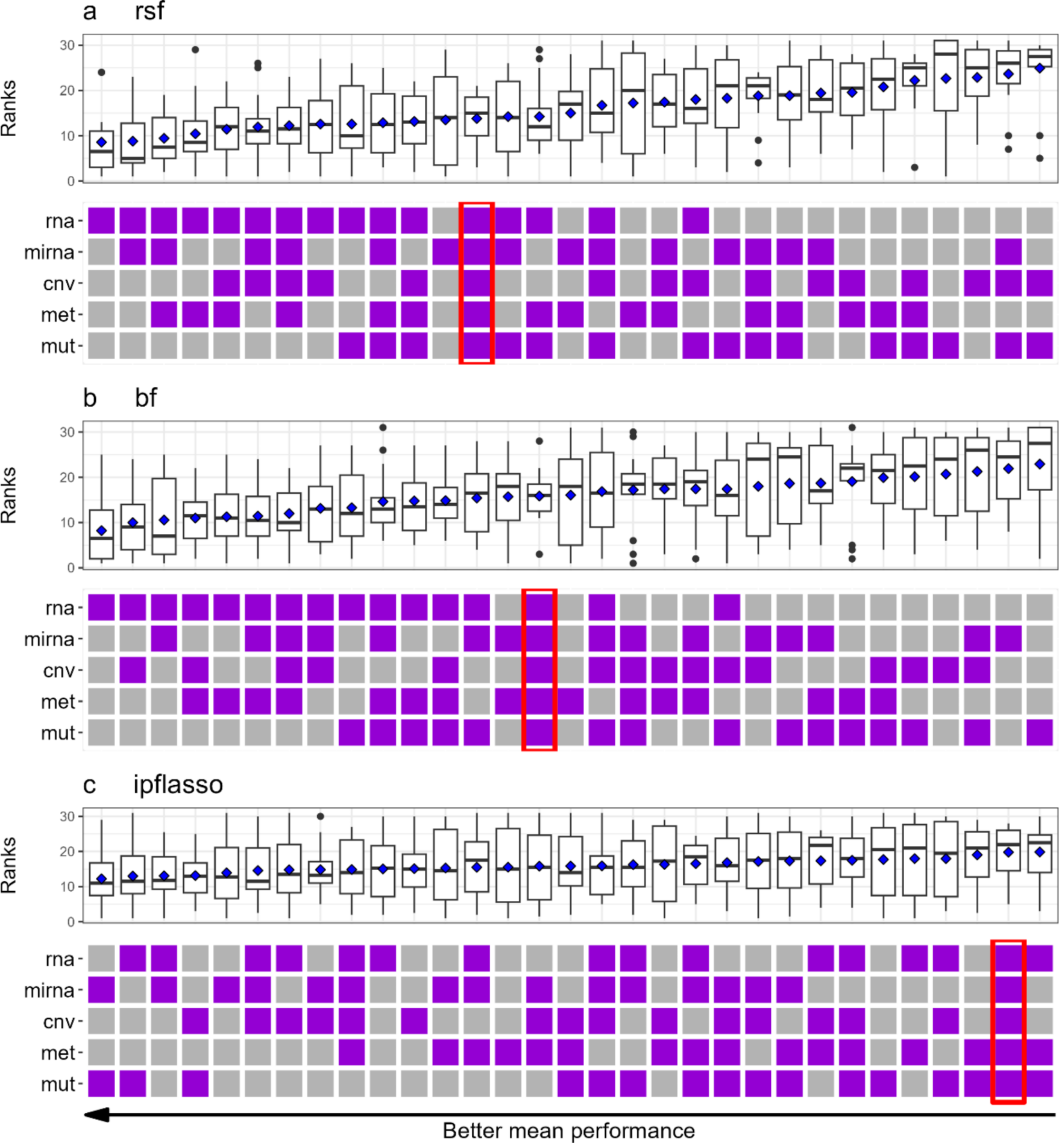
Dataset-specific ranks of each block combination (ibrier). The ranks of each combination among all 31 combinations are shown. The purple squares indicate which omics block(s) were included in the respective combinations. The values shown by the boxplots are the ranks achieved across all 14 datasets, where the blue diamonds represent the means of the ranks. The upper (**a**), middle (**b**), and lower (**c**) panels show the results obtained for rsf, bf, and ipflasso, respectively. Smaller ranks indicate a better predictive performance. The combinations are sorted in increasing order according to the mean ranks across the datasets, which is why the combinations further to the left tend to perform better. The combinations using all five blocks are marked with red boxes. cnv: CNV, mirna: miRNA, mut: DNAseq, met: methylation, rna: mRNA

Figure 1 shows, for each prediction method, the rankings achieved by each block combination among all 31 possible block combinations for all datasets. Figures S1 to S4 in Additional File 1 display these results for the raw cross-validated ibrier and cindex values for all prediction methods. The raw performance measure values for each combination of block combination, dataset, and prediction method are detailed in Tables S2 and S3 in Additional File 1. Although the raw performance measure values provide direct insights into the absolute performance of the various block combinations for different methods, comparing these values across different datasets is challenging due to the widely varying signal strengths among the datasets. For this reason, the following descriptions are based on the ranks rather than the raw performance measure values.

The results differ quite considerably across the different prediction methods. However, a consistent observation we can make is that the best performances were achieved with one to three blocks. Adding more blocks did not deliver better predictive performance, but actually tended to lead to worse results. For rsf and bf, we see that mRNA was very important for prediction, as the best-performing block combinations all included mRNA. Apart from the latter specific observation, there is no clear picture regarding the importance of each individual block. In general, the boxplots in Fig. 1 reveal that the results differ quite strongly across the datasets, particularly for ipflasso. The results obtained for lasso and prioritylasso are shown in Figure S5 in Additional file 1. Interestingly, lasso was the only method for which using more blocks tended to deliver better prediction results. For prioritylasso, we again see a clear trend towards worse predictive performance for block combinations with many blocks, while the best results were obtained with single blocks. In the next subsection it will, however, be seen that the use of prioritylasso tended to lead to worse prediction results than the other prediction methods. While we do see differences in the results obtained for the cindex (Figures S6 and S7 in Additional file 1), the general conclusions are very similar to those obtained with the ibrier. Exceptions are that for lasso we no longer observe a trend towards better predictive performance by including more blocks, and that for ipflasso there was less variability of the results across datasets.

There is a possibility that the number of observations in the available datasets is not sufficient to adequately exploit the predictive information contained in combinations with many omics blocks. If this were the case, contrary to the results described above, combinations with many blocks might outperform combinations with fewer blocks for large datasets. If so, a trend should be observable where combinations with many blocks rank better for larger datasets than for smaller datasets. Conversely, a trend should be observed where combinations with fewer blocks rank worse for larger datasets than for smaller ones. We investigated this using the available datasets in an analysis described in detail in Additional file 1. To summarize, this analysis did not suggest that combinations with many blocks would benefit from larger datasets in prediction. Even though the number of datasets included in our benchmark experiment is comparably large, we still must consider that the mean ranks obtained for the block combinations are associated with considerable variability. This was already indicated by the large variances observed in the boxplots showing the results obtained for the different datasets. To assess statistical uncertainty we performed bootstrap analysis [42, 43] at the level of the 14 included datasets. This analysis was used to construct 95% confidence intervals for the means of the dataset-specific ranks for each combination of block combination and prediction method. Through this approach, we investigated whether the mean ranks for the best-performing combinations were statistically significantly different from those involving all five omics blocks. This was the case for all methods except lasso, where the confidence intervals were very wide. The results obtained for the two performance measures were quite similar, where the confidence intervals for the cindex tended to be narrower. A detailed description of this analysis and its results can be found in Additional file 1.

### Ranking of the predictive performance of all prediction methods for all block combinations

**Fig. 2:**
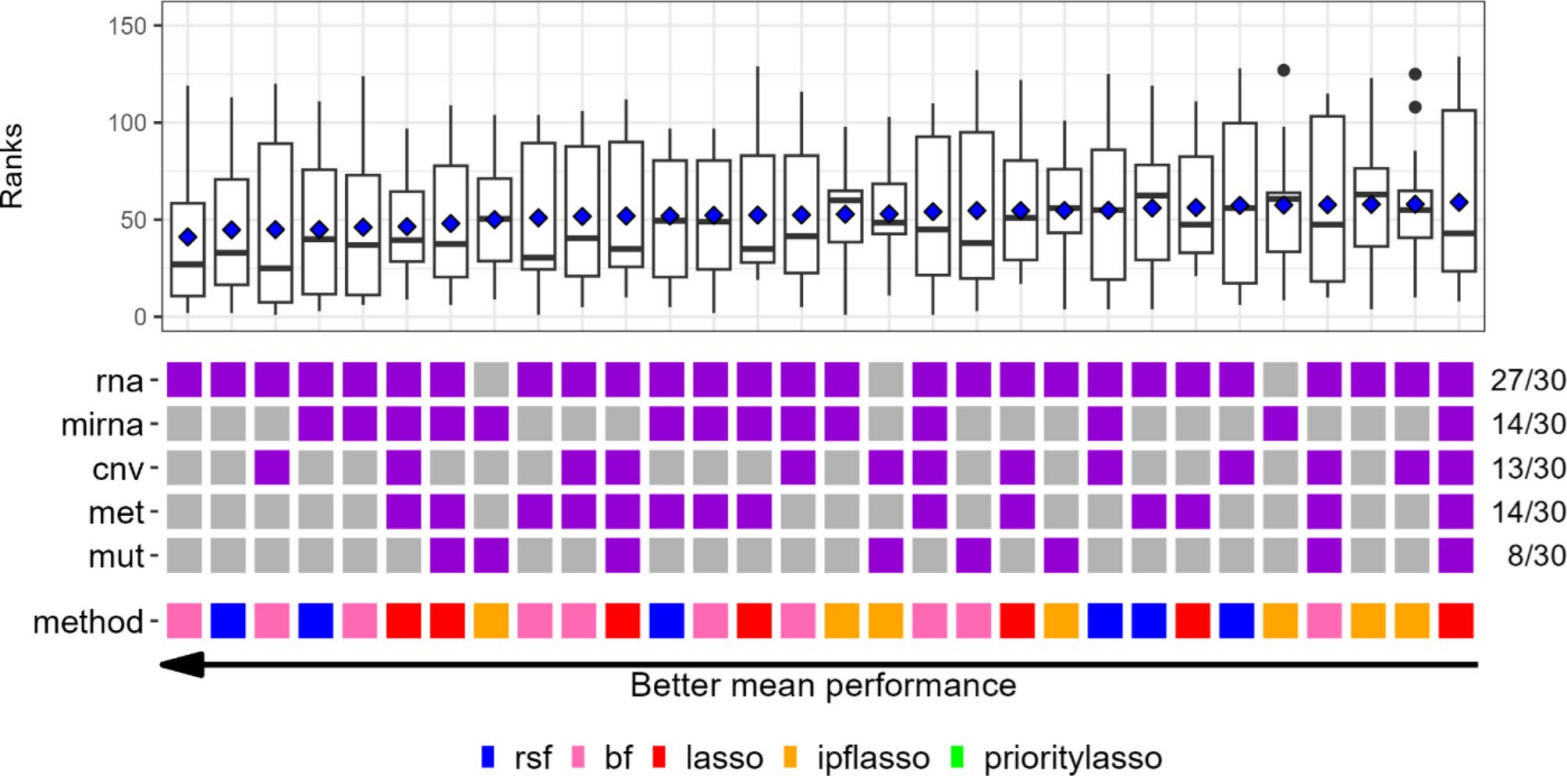
Dataset-specific ranks of each combination of prediction method and blocks (ibrier). The ranks of each combination among all 155 combinations of prediction methods and blocks are shown. The purple squares indicate which omics block(s) were included in the respective combinations. The values shown by the boxplots are the ranks achieved across all 14 datasets, where the blue diamonds represent the means of the ranks. Smaller ranks indicate a better predictive performance. The combinations are sorted in increasing order according to the mean ranks across the datasets, which is why the combinations further to the left tend to perform better. For reasons of clarity, only the 30 combinations with the smallest positions are shown. cnv: CNV, mirna: miRNA, mut: DNAseq, met: methylation, rna: mRNA

In the previous subsection, we analyzed the results per prediction method. This analysis did not allow us to judge which combinations of prediction methods and blocks tend to deliver the best prediction results. Figure 2 shows, for all datasets, the ranking achieved by each prediction method-block combination among all 155 prediction method-block combinations. For clarity, only the 30 combinations with the lowest positions are shown. The corresponding results for the cindex are shown in Figure S12 in Additional file 1.

The prediction method bf occurred the most often in the 30 best combinations, and rsf, lasso, and ipflasso occurred about equally frequently in these combinations. The method prioritylasso was not featured in the best combinations. Almost all of the best combinations featured mRNA, and the two best combinations used only mRNA. We used statistical testing based on the hypergeometric distribution to evaluate whether this frequent occurrence of mRNA in the top 30 combinations could be attributable to random chance. This was found to be highly unlikely (p = $\:5.9\times\:{10}^{-6}$). Details of the statistical test procedure can be found in Additional file 1.

If all 155 combinations were equally important in prediction, the expected number of combinations in the 30 best combinations that feature a particular block would be 15.5. Against this background, the expected number of combinations that feature a particular block is 15.5, Fig. 2 reveals that the remaining blocks were not overrepresented in the top 30 combinations. In particular, DNAseq was featured only in eight of the top 30 combinations. Nevertheless, there is again a large variability between the results obtained for the different datasets. Interestingly, for the cindex (Figure S12 in Additional file 1), lasso was featured by far the most frequently in the top 30 combinations. This result seems surprising at first, considering that lasso was among the worst-performing methods in the benchmark studies of [2] and [3]. However, in contrast to these previous benchmark studies, we did not penalize the coefficients of the clinical covariates. This likely explains why lasso performed much better in our benchmark study given the high predictive importance of clinical covariates. A disadvantage of the lasso, seen in Fig. 2 and Figure S12 (Additional file 1), is that it tends to require more blocks than the other methods. The majority of the 30 best combinations featured mRNA also for the cindex. Again, it is important not to over-interpret details of the obtained results, as the variability across the different datasets is large here as well. Figures S13 and S14 in Additional file 1 present versions of Fig. 2 and S12, displaying the raw cross-validated ibrier and cindex values in the boxplots instead of their corresponding ranks.

### Best-performing combinations of prediction methods and blocks per dataset

As seen above, the rankings achieved by the different combinations of prediction methods and blocks varied widely between the datasets. It is interesting to learn which prediction methods and block combinations are most successful for which datasets. Table 3 shows, for each dataset, the combinations of prediction methods and blocks associated with the smallest cross-validated ibrier values and the largest cross-validated cindex values. For the great majority of datasets, the best performance was achieved using only up to two blocks. Statistical tests based on the binomial distribution confirmed that this is unlikely to occur by chance, with p-values of 0.022 for ibrier and $\:3.9\times\:{10}^{-5}$ for cindex. For a detailed description of the testing procedure, refer to Additional file 1.

We observed quite large variability in the performance of the block combinations across the datasets, and, for each dataset, between the two performance measures. While it is not clear how much of this is due to random variation, it is congruent with the observation made in the previous subsections that there is large variability in the ranks of the block combinations across datasets. For more than half of the datasets, mRNA was used, with miRNA in second place.

In the case of the cindex, for five datasets only mRNA was used. Another difference observed between the results obtained for the ibrier and the cindex is that methylation data was used quite frequently in the case of the ibrier, but only for one dataset in the case of the cindex.

For the cindex, which assesses discrimination alone, the best-performing combinations tended to use few blocks. This suggests that a small number of blocks may be sufficient to achieve good discrimination. Conversely, for the ibrier, which assesses both discrimination and calibration, the best-performing combinations tended to have more blocks. This pattern suggests that achieving good calibration in addition to discrimination may require the integration of more blocks.

Regarding the prediction methods, we do not see a clear winner. For both performance measures, each prediction method was used at least for one dataset.

**Table 3:** The best-performing combinations of prediction methods and blocks per dataset. Cnv: CNV, mirna: miRNA, mut: DNAseq, met: methylation, rna: mRNA

	ibrier		cindex	
dataset	prediction method	blocks	prediction method	blocks
BLCA	lasso	rna, mirna	prioritylasso	rna
BRCA	bf	rna, met	bf	rna, mirna
COAD	bf	met	bf	rna, mut
ESCA	ipflasso	mut	rsf	mirna, mut
HNSC	ipflasso	rna, mirna	rsf	rna
LGG	ipflasso	met, cnv	prioritylasso	rna
LIHC	bf	rna, mirna, met, cnv	lasso	rna, cnv
LUAD	lasso	mirna	ipflasso	mut
LUSC	ipflasso	mirna, met	prioritylasso	rna, mirna
PAAD	prioritylasso	rna	bf	rna
SARC	prioritylasso	met, cnv	rsf	mirna, met, mut
SKCM	lasso	rna, mut, cnv	bf	rna
STAD	rsf	rna, mirna, mut	rsf	mirna
UCEC	bf	rna, cnv	rsf	mirna

In the previous paragraph, we noted that mRNA and miRNA were the most frequent blocks in the optimal combinations. We again used statistical testing to evaluate whether the occurrence of these blocks significantly exceeds what would be expected by random chance. Here, only mRNA was found to be statistically significantly overrepresented in the best-performing combinations, and solely for the cindex (p-value: 0.031). However, these findings should be interpreted considering the limited number of cases (14, the number of datasets), which means that these tests may have relatively low statistical power. We refer the interested reader to Additional file 1 for details on the statistical testing procedure.

In Table 3, we observed that the block combinations that yielded the best predictive performance varied considerably between the different datasets. At the same time, mRNA and miRNA were the most frequent blocks in these optimal combinations (although only partially statistically significant). Consequently, we examine a crucial practical question: Is the availability of only mRNA and miRNA typically sufficient to achieve near-optimal predictive performance compared to the availability of all blocks?

To investigate this, we conducted an analysis of the performance rankings of all block combinations by dataset, presented in detail in Additional file 1. Here combinations involving only mRNA or miRNA (excluding other blocks, i.e., solely mRNA, miRNA, or a combination of both) ranked in the top 30% for all datasets and both performance measures. For the ibrier, after additionally including the methylation data for the datasets COAD, LGG, LIHC, LUSC, and SARC, top 10% rankings were achieved for all datasets. This additional inclusion of methylation data also enhanced the rankings for the cindex. For both performance metrics, the best combinations after additional consideration of methylation data outperformed those using all five blocks across all datasets. For further insights into this analysis, interested readers are referred to Additional file 1.In summary, when only mRNA and miRNA are available, it is generally possible to achieve predictive performance close to that attainable when all considered blocks are available for potential use. However, for certain datasets, the additional inclusion of methylation data can lead to improved prediction results

## Discussion

Despite notable variations in outcomes across different datasets, in our analysis, predictive models incorporating the entire array of available omics data consistently exhibited poorer performance compared to models using only a subset of omics blocks. This challenges the prevailing approach in multi-omics data prediction and suggests that maximal utilization of diverse omics blocks is not always optimal, despite the limitations present in our study (see below).

Moreover, it is important to emphasize that not all omics blocks are equal in their predictive capabilities. Our findings underscore the importance of data source in this context. Specifically, among the various omics data blocks, mRNA data emerged as the most informative and impactful. In most cancer types, predictive performance achieved through combinations of mRNA and miRNA approached the levels attainable with the complete spectrum of analyzed omics blocks. Notably, in specific instances, the incorporation of methylation data contributed to additional enhancements in predictive performance.

Focusing on mRNA, miRNA, and, for certain cancer types, additional methylation data, not only enhances predictive performance but also contributes to resource conservation in terms of time, materials, and finances. Furthermore, the field of multi-omics prediction faces the challenge that the data to which the prediction models are applied often do not contain all of the necessary blocks required by those models, complicating their application [44, 45]. The use of fewer blocks for prediction modeling is anticipated to mitigate this issue. Note that while focusing on a few blocks seems to be beneficial for prediction, integrating many blocks in multi-omics data is informative for understanding cancer biology [46, 47].

As described in the ”Background” section, other studies have also compared different combinations of blocks with respect to their predictive performance [2, 3, 13–17]. Our results are consistent with these studies despite the differences in study designs (refer to the “Background” section for details). The study detailed in this paper is unique in that it used a multitude of datasets and survival prediction methods to examine all possible combinations of available omics blocks. This approach enables more reliable conclusions about the efficacy of different block combinations. Crucially, due to the overlap and interaction of predictive information among blocks, it was important to consider all possible combinations, rather than limiting the analysis to combinations where each block independently carries substantial predictive information. Based on the results of our study, future investigations need not evaluate every possible combination. Instead, combinations containing blocks that did not improve prediction when combined with others in our study could be excluded.

As depicted in Figs. 1 and 2, the rankings of distinct block combinations exhibited pronounced variations across diverse datasets. Consequently, as illustrated in Table 3, varying cancer types called for distinct optimal block combinations. Remarkably, for certain datasets, neither mRNA nor miRNA were part of the optimal block combinations. These findings emphasize that there is no universally superior block combination that outperforms all others across all datasets. However, when interpreting the results in Table 3, it should also be noted that for many datasets, several similar combinations yielded similar performance to the best-performing one (results not shown). This suggests that the findings in Table 3 may be influenced by random variation. Similar statements can be made with respect to Figs. 1 and 2, where the differences in performance between the best-performing combinations were small. The large variability observed in our study emphasizes the importance of large-scale benchmark studies using many datasets, as performed in this paper. It is well known that many observations are necessary to draw valid statistical conclusions, which is due to the large variability between these observations. However, this issue is often overlooked when designing benchmark experiments where the datasets play the roles of the observations [48]. It is common in published benchmark studies that only few (e.g., 5 to 7) datasets are considered. This limitation is occasionally due to the limited availability of suitable datasets in certain fields.

The ranks of the different block combinations also varied quite strongly between the considered prediction methods. However, we did not observe structural differences between methods that do (bf, ipflasso, prioritylasso) and do not consider the group structure of the multi-omics data (rsf, lasso). The best-performing prediction models (Fig. 2) also included many prediction methods that do not consider the group structure of the multi-omics data. In contrast, in the large-scale benchmark studies by Herrmann et al. [2] and Hornung and Wright [3], most prediction methods that consider the group structure outperformed those that do not. This discrepancy can likely be explained by the fact that we prioritized the clinical covariates also for those methods that do not consider the group structure (see “Experimental settings” section), which was not done in Herrmann et al. [2] and Hornung and Wright [3]. In addition, Nießl et al. [49] have shown that the results of benchmark studies in general are variable and sensitive to analytic choices even if large numbers of datasets are used.

Lastly, the results also varied between the two considered performance metrics. The concordance index is not a scoring rule as it only measures discrimination. It is not suitable in situations where the interest is in predicting the risk for a given time horizon [50]. Additionally, Harrell’s version of the concordance index has been shown to be influenced by the censoring distribution [51], and its estimator is increasingly biased for higher censoring rates [52, 53]. Uno et al. proposed an alternative version of the concordance index that is not affected by the censoring distribution [51]. Pencina et al. [54] demonstrated notable variations in estimates obtained from different versions of the concordance index. However, in our benchmark study, our focus was not on the absolute cindex values but on the relative performances of different block combinations. Therefore, the bias in the cindex is of lesser concern here, provided it is consistent across the different combinations.

In contrast, the ibrier assesses both discrimination and calibration. As a scoring rule, it evaluates the quality of survival function predictions, offering a more comprehensive assessment of the predictive performance of models. Therefore, the ibrier should be considered as the primary measure of predictive accuracy.

Given the strong variability across datasets it is difficult to judge how strongly the aggregated results are affected by random variation. We took great care not to interpret details of the obtained results but focused on general observations that could be made across the different prediction methods and performance metrics. Using bootstrap analysis, we were able to strengthen important broad patterns in the results by accounting for result variability among datasets. Additionally, we conducted a series of statistical tests to examine the robustness of a limited number of specific key aspects of the results. This approach was adopted to minimize the risk of generating false positive results.

By prioritizing the clinical covariates, we exploited the predictive information contained in them to a large degree. Given that the predictive information contained in the clinical covariates and the omics features is overlapping, it might be assumed that, if we had not prioritized the clinical covariates, more omics blocks would have been necessary to achieve optimal predictive performance. However, this seems unlikely because few blocks were necessary for almost all datasets, the number of clinical covariates varied widely across datasets, and we made the same observation in the case of ipflasso, the only method for which we did not prioritize the clinical covariates. Irrespective of this, it is always important to prioritize the clinical covariates to exploit their strong predictive information. Furthermore, including clinical covariates is typically feasible, as they are cost-effective and easily obtainable, both in the development and application of the prediction model.

We did not include models that use only the clinical variables in the benchmark study. Unlike the omics blocks, where the included variables within the same block are very similar across different data sources, the same cannot be expected for the clinical variables. The specific sets of clinical variables available in the datasets used in our analysis will often not be available in applications. It can be safely assumed that the predictive performance of models that rely solely on clinical variables strongly depends on the specific clinical variables used. For this reason, the performance of such models in our benchmark study would likely not have been representative of real-world analyses. This would have been problematic considering our goal was to draw generalizable conclusions.

However, it is important to emphasize that models based exclusively on clinical variables can achieve high predictive performance. The large-scale comparison study by Herrmann et al. [2] demonstrated that such models can outperform those based on multi-omics data. Additionally, in the field of predictive modeling based on single-omics data, it is known that omics data often do not provide an additive predictive value over clinical variables alone [37, 55]. Therefore, it is crucial to also evaluate the predictive performance of models based solely on the clinical variables. This evaluation can prevent the unnecessary use of complex omics predictive models in situations where simpler clinical models are equally effective or superior.

There exists a possibility that current prediction methods for multi-omics data do not optimally exploit the interplay among multiple omics blocks. If so, it would be possible to develop methods that leverage this interplay very efficiently, potentially leading to improved predictive performance with multiple omics blocks, contrary to our findings. However, this may be a challenging endeavor. Across all methods in our benchmark study, combinations with fewer blocks consistently outperformed those with all available blocks. Additionally, the studies by Wissel et al. [15], Vale-Silva and Rohr [16] and Osipov et al. [17], which included several methods not used in our study, support our observations. These studies also found that selected block combinations yielded better prediction results compared to the use of all blocks. Given the consistency of these findings across diverse methods, it is crucial to properly address the challenges associated with multi-omics data, particularly overlapping predictive information and feature interactions across omics blocks, in the development of future prediction methods. The results of our benchmark study may offer valuable insights for methodological researchers in this field. Beyond predictive performance, future prediction methods for multi-omics data could place greater emphasis on sparsity and interpretability. A recent notable example of a method that prioritizes these aspects is Stabl [56].

In our benchmark study, we employed prediction methods most commonly used in previous benchmarks. However, these previous studies included varying methods. Future benchmark studies could broaden their scope to include an even wider range of prediction methods, further enhancing the generalizability of the results. A particular challenge in this effort is that these methods are often implemented in different software environments, complicating direct comparisons within a unified benchmark setting. The benchmark study by Wissel et al. [15] is exemplary in this context. Wissel et al. compared (multi-)omics prediction methods implemented in both R and Python. They have made their code publicly available on GitHub, enabling others to adopt a similar approach. As noted above, different datasets were associated with markedly different best-performing block combinations in our study. While this result can be expected to be subject to some degree to random variation, it is also likely to reflect different information structures across cancer entities. It may be the subject of future research whether this formal result can be related to specific biological information structures within specific cancer entities.

In the following we will discuss several limitations of our study. We exclusively used multi-omics datasets from TCGA, which offers the currently largest collection of such datasets. To draw broader conclusions, we implicitly assumed these datasets are representative of multi-omics datasets generally beyond TCGA. In particular, when performing statistical inference using bootstrap analysis and statistical tests, we treated the TCGA multi-omics datasets as a random sample from the entire spectrum of potential multi-omics datasets.

While the quality of the omics data in TCGA datasets has often been praised, there have been concerns about the accompanying clinical data [57]. In particular, it has been noted that the follow-up interval in these data is relatively short [58], resulting in comparatively large proportions of censored survival times. In addition, it has been noted that data from databases containing processed versions of TCGA data can feature error-prone survival information [59]. However, the latter is not an issue in our study because we used the data provided directly by TCGA.

We avoided interpreting finer details of the results to reduce the risk of obtaining nongeneralizable results. However, this risk cannot be excluded, especially given the relatively short follow-up time in TCGA, the impact of preprocessing, and the heterogeneity between data from different sources. Given these issues, more open-source cancer datasets with survival outcomes from different sources are needed. These would provide a good basis for further benchmark studies on various topics. Such benchmark studies are critical in areas such as predictive modeling based on (multi-)omics data, where analytical results are difficult to obtain due to the complexity of these data.

We used exclusively overall survival as the outcome. Depending on the application, progression-free survival can also be relevant. Typically, there is only a modest correlation between overall survival and progression-free survival [60, 61]. A statistical benefit of using progression-free survival is the reduced number of censored observations, as the time to progression is shorter than the time to death. This advantage becomes more important in cancers with longer survival. However, the definition of progression-free survival can vary between studies and its measurement can contain subjective components. In contrast, overall survival is clearly defined, which makes it better comparable across studies. Consequently, overall survival is likely more appropriate for benchmark studies like ours, where the aim is to draw generalizable conclusions.

We did not investigate how sensitive our results are to the number of features selected. The choice of 2,500 features was not based on statistical criteria. Instead, this number was selected as a compromise, balancing the need to capture the relevant predictive information from the omics blocks with maintaining computational demands consistent with practical applications.

However, our findings align with those of previous studies that used different numbers of features. For example, Wissel et al. [15] did not perform feature selection and found that models based solely on mRNA typically outperformed those that incorporated all available omics blocks. Similarly, Vale-Silva et al. [16] and Osipov et al. [17], who selected fewer features than in our study, observed that models with fewer omics blocks generally provided better predictive performance than those incorporating all blocks. Given these previous findings, our results are likely sufficiently robust to the specific number of features selected.

The multi-omics analyses conducted in our study do not pertain to single-cell omics datasets. Whether comparable results can be found in this context should be considered in future analyses.

## Conclusions

The use of multi-omics data to predict clinical outcomes has been an active and productive area of research in recent years. However, obtaining such data is complex and costly, which is why for prediction purposes it would be beneficial to only collect omics data types that contribute to improving the predictive performance. Note that in contrast, if the goal is to better understand cancer biology, the integration of multiple omics data types is likely always beneficial in multi-omics data analysis.

In the extensive benchmark study outlined in this paper, in alignment with prior findings, we observed that the amalgamation of numerous omics data types can impede the effectiveness of multi-omics survival prediction. Our results strongly suggest that employing only a handful of data types tends to yield superior performance. In most instances, leveraging mRNA alone or in combinations with miRNA is sufficient. Yet, for certain cancer types, the inclusion of methylation data demonstrates an ability to enhance predictions.

We anticipate that our results will augment the predictive potential of multi-omics data within the field, simultaneously optimizing resource allocation and minimizing endeavors.

### Electronic supplementary material

Below is the link to the electronic supplementary material.

Supplementary Material 1

## Data Availability

All R code written to produce and evaluate our results is available on GitHub (https://github.com/YingxiaLi2023/multi-omics-data, commit hash: 5531dcea6f63a08fe9f1c02e53b7cc0666751227, accessed on August 12, 2024).

## Abbreviations

TCGA: The Cancer Genome Atlas
RF-VI: The permutation-based variable importance measure of random survival forests
rsf: Random Survival Forests
bf: Block Forest
lasso: The Least Absolute Shrinkage and Selection Operator
ipflasso: The Two-Step Integrative Lasso with Penalty Factors
prioritylasso: Priority-Lasso
cindex: Harrell’s Concordance Index
ibrier: Integrated Brier Score
