# Supplementary material for: Does combining numerous data types in multi-omics data improve or hinder performance in survival prediction? Insights from a large-scale benchmark study
Source: BMC Med Inform Decis Mak. 2024 Sep 2;24:244. doi: 10.1186/s12911-024-02642-9 (PMC11370316; doi:10.1186/s12911-024-02642-9)
Supplement: Supplementary file 1 — Supplementary Material 1 [file 12911_2024_2642_MOESM1_ESM.docx]

**Detailed overview on existing benchmark studies that compared the predictive performance of different block combinations**

A pioneering comparative study by Zhao et al. [1] considered four blocks. They used four datasets and prediction methods that do not take the multi-omics structure into account (more sophisticated methods were not yet available at the time of conducting their analysis). It was observed in that study that once mRNA data and clinical covariates were included in the model, the addition of any further blocks did not substantially improve prediction results.

Gómez-Rueda et al. [2] considered four blocks, performing predictive modeling using each block individually and concurrently using all blocks taken together. Firstly, the integration of four blocks produced a slightly better model performance than using any of the single blocks, and secondly, for the individual blocks, the best predictions were obtained using mRNA, followed by miRNA and copy number variation.

Hornung and Wright [3] used 20 TCGA multi-omics datasets, encompassing four omics blocks and clinical covariates, to compare the predictive performance of various multi-omics prediction methods. They compared the predictive performance not only for clinical covariates combined with all omics blocks but also for clinical covariates combined with mRNA only. In general, the predictive performance was better in the latter case.

Herrmann et al. [4] performed a benchmark study based on 18 TCGA multi-omics cancer datasets with four omics blocks and clinical covariates and found that the predictive performance of multi-omics data using state-of-the-art prediction models is limited. Here, only one prediction method based on multi-omics data (slightly) outperformed the clinical model. However, Herrmann et al. always used all blocks for prediction without considering different combinations of omics blocks.

Wissel et al. [5] conducted another benchmark study using 17 TCGA multi-omics cancer datasets comprising six omics blocks and clinical covariates. The primary goal was to evaluate the robustness of various multi-omics prediction methods with respect to uninformative blocks. Their findings indicated that current prediction methods are indeed sensitive to the presence of uninformative blocks to varying degrees. Consequently, they recommended that, pending the development of more noise-resistant methods, only blocks known to be critical for prediction should be used. However, prior to the study outlined in our present paper, there was a lack of comprehensive empirical evidence indicating which combinations of blocks produce the most accurate predictions for various types of cancer. Similar to Hornung and Wright, Wissel et al. also compared the predictive performance of using clinical covariates with mRNA data versus using clinical covariates with all omics blocks. Consistent with Hornung and Wright, they found that for the vast majority of prediction methods, the former approach resulted in better predictions on average across datasets and in most individual datasets. Another notable observation by Wissel et al. was the relatively poorer calibration of deep learning models compared to other models.

Vale-Silva and Rohr [6] compared the predictive performance of a number of combinations from four omics blocks and image data on a large TCGA multi-omics dataset encompassing 33 cancer types, employing the deep learning method for multi-omics data presented in their paper. Clinical covariates were consistently included in their analyses. The combinations of clinical + mRNA, clinical + methylation data, and clinical + mRNA + methylation data provided the most accurate predictions.

To the best of our knowledge, a study by Osipov et al. [7] is the only one that, like ours, evaluated all possible combinations of omics blocks. This study considered ten different omics blocks and focused on a binary outcome (survival: yes vs. no) using a single, relatively small dataset of pancreatic adenocarcinoma patients (74 samples). Osipov et al. found, consistent with our results, that using only a few omics blocks is sufficient for prediction. However, unlike us, they concluded that models with few omics blocks do not necessarily perform better but can achieve similar predictive performance to models using more blocks. The most critical omics blocks for prediction in their study were plasma protein and mRNA. A distinctive aspect of their study is the external validation of models on TCGA data, where, however, only a subset of the ten omics blocks used in the training data was available.

**Clinical covariates used for each dataset**

Table S1: Overview of clinical covariates used for at least three datasets. The numbers indicate usage, where “1” represents “used” and “0” represents “not used”. Variable labels in the table and their corresponding names in the processed datasets are as follows: “Age” is “age”, “Gender” is “gender_MALE”, “Histologic Grade G3” is “neoplasm_histologic_grade_G3”, “Positive Lymph Nodes” is “number_of_lymphnodes_positive_by_he”, “Pathologic Stage II” is “stage_event_pathologic_stage_Stage.II”, “Pathologic Stage III” is “stage_event_pathologic_stage_Stage.III”, and “Smoking History” is “tobacco_smoking_history”.

|  | Age | Gender | Histologic Grade G3 | Positive Lymph Nodes | Pathologic Stage II | Pathologic Stage III | Smoking History |
| --- | --- | --- | --- | --- | --- | --- | --- |
| BLCA | 1 | 1 | 0 | 0 | 0 | 1 | 0 |
| BRCA | 1 | 0 | 0 | 1 | 0 | 0 | 0 |
| COAD | 1 | 1 | 0 | 1 | 1 | 1 | 0 |
| ESCA | 1 | 1 | 0 | 0 | 1 | 1 | 0 |
| HNSC | 1 | 1 | 1 | 0 | 0 | 0 | 1 |
| LGG | 1 | 1 | 0 | 0 | 0 | 0 | 0 |
| LIHC | 1 | 1 | 0 | 0 | 1 | 0 | 0 |
| LUAD | 1 | 1 | 0 | 0 | 0 | 1 | 1 |
| LUSC | 1 | 1 | 0 | 0 | 0 | 0 | 1 |
| PAAD | 1 | 1 | 1 | 1 | 0 | 0 | 1 |
| SARC | 1 | 1 | 0 | 0 | 0 | 0 | 0 |
| SKCM | 1 | 1 | 0 | 0 | 1 | 1 | 0 |
| STAD | 1 | 1 | 1 | 1 | 1 | 0 | 0 |
| UCEC | 1 | 0 | 0 | 0 | 0 | 0 | 0 |

There were 75 clinical covariates across all datasets. Table S1 summarizes the availability of the seven clinical covariates used for at least three of the 14 datasets. Additionally, nine clinical covariates were used for two datasets each, while the remaining 59 were used for only one dataset.

Below is a list of the clinical covariates used for each dataset, with the number of variables used indicated in parentheses:

- **BLCA (5)**: age, diagnosis_subtype_Papillary, gender_MALE, stage_event_pathologic_stage_Stage.III, stage_event_pathologic_stage_Stage.IV
- **BRCA (8)**: age, breast_carcinoma_estrogen_receptor_status_Positive, breast_carcinoma_progesterone_receptor_status_Positive, breast_carcinoma_surgical_procedure_name_Modified.Radical.Mastectomy, breast_carcinoma_surgical_procedure_name_Other, breast_carcinoma_surgical_procedure_name_Simple.Mastectomy, histological_type_Infiltrating.Lobular.Carcinoma, number_of_lymphnodes_positive_by_he
- **COAD (7)**: age, gender_MALE, lymphatic_invasion_YES, number_of_lymphnodes_positive_by_he, stage_event_pathologic_stage_Stage.II, stage_event_pathologic_stage_Stage.III, venous_invasion_YES
- **ESCA (6)**: age, gender_MALE, primary_pathology_histological_type_Esophagus.Squamous.Cell.Carcinoma, primary_pathology_residual_tumor_R0, stage_event_pathologic_stage_Stage.II, stage_event_pathologic_stage_Stage.III
- **HNSC (11)**: age, alcohol_history_documented_YES, gender_MALE, lymphnode_neck_dissection_YES, neoplasm_histologic_grade_G1, neoplasm_histologic_grade_G2, neoplasm_histologic_grade_G3, stage_event_clinical_stage_Stage.II, stage_event_clinical_stage_Stage.III, stage_event_clinical_stage_Stage.IV.B.C, tobacco_smoking_history
- **LGG (10)**: age, gender_MALE, histological_type_Oligoastrocytoma, histological_type_Oligodendroglioma, laterality_Right, motor_movement_changes_YES, sensory_changes_YES, tumor_location_Supratentorial..Not.Otherwise.Specified, tumor_location_Supratentorial..Temporal.Lobe, visual_changes_YES
- **LIHC (11)**: age, albumin_result_specified_value, creatinine_value_in_mg_dl, fetoprotein_outcome_value, fibrosis_ishak_score_1.2...Portal.Fibrosis, fibrosis_ishak_score_5.6...Incomplete.Established.Cirrhosis, gender_MALE, stage_event_pathologic_stage_Stage.I, stage_event_pathologic_stage_Stage.II, vascular_tumor_cell_type_Micro, vascular_tumor_cell_type_None
- **LUAD (9)**: age, anatomic_neoplasm_subdivision_L.Upper, anatomic_neoplasm_subdivision_R.Lower, anatomic_neoplasm_subdivision_R.Upper, gender_MALE, stage_event_pathologic_stage_Stage.IB, stage_event_pathologic_stage_Stage.IIA, stage_event_pathologic_stage_Stage.III, tobacco_smoking_history
- **LUSC (9)**: age, anatomic_neoplasm_subdivision_L.Upper, anatomic_neoplasm_subdivision_R.Lower, anatomic_neoplasm_subdivision_R.Upper, gender_MALE, stage_event_pathologic_stage_Stage.IB, stage_event_pathologic_stage_Stage.IIA, stage_event_pathologic_stage_Stage.IIIA, tobacco_smoking_history
- **PAAD (10)**: age, anatomic_neoplasm_subdivision_Head.of.Pancreas, gender_MALE, histological_type_Pancreas.Adenocarcinoma.Other.Subtype, maximum_tumor_dimension, neoplasm_histologic_grade_G3, number_of_lymphnodes_positive_by_he, stage_event_pathologic_stage_Stage.I, surgery_performed_type_Whipple, tobacco_smoking_history
- **SARC (11)**: age, gender_MALE, primary_pathology_histological_type_Leiomyosarcoma..LMS., primary_pathology_histological_type_Myxofibrosarcoma, primary_pathology_histological_type_Undifferentiated.Pleomorphic.Sarcoma, primary_pathology_metastatic_diagnosis_YES, primary_pathology_tumor_tissue_sites_Lower.Extremity, primary_pathology_tumor_tissue_sites_Retroperitoneum.Upper.abdominal, primary_pathology_tumor_total_necrosis_percent_0...no.necrosis.or.no.mention.of.necrosis., primary_pathology_tumor_total_necrosis_percent_Moderate.Necrosis....10...50.., radiation_therapy_YES
- **SKCM (9)**: age, breslow_depth_value, gender_MALE, melanoma_ulceration_indicator_YES, stage_event_pathologic_stage_Stage.II, stage_event_pathologic_stage_Stage.III, stage_event_pathologic_stage_Stage.IIIC, tumor_tissue_site_Primary.Tumor, tumor_tissue_site_Regional.Lymph.Node
- **STAD (7)**: age, gender_MALE, neoplasm_histologic_grade_G3, number_of_lymphnodes_positive_by_he, stage_event_pathologic_stage_Stage.II, stage_event_pathologic_stage_Stage.IIIA, stage_event_pathologic_stage_Stage.IIIC
- **UCEC (11)**: age, histological_type_Serous.endometrial.adenocarcinoma, neoplasm_histologic_grade_G2, neoplasm_histologic_grade_High.Grade, pct_tumor_invasion, stage_event_clinical_stage_Stage.IB, stage_event_clinical_stage_Stage.IC, surgical_approach_open, total_aor_lnr, total_pelv_lnr, weight

**Mean cross-validated performance metric values**


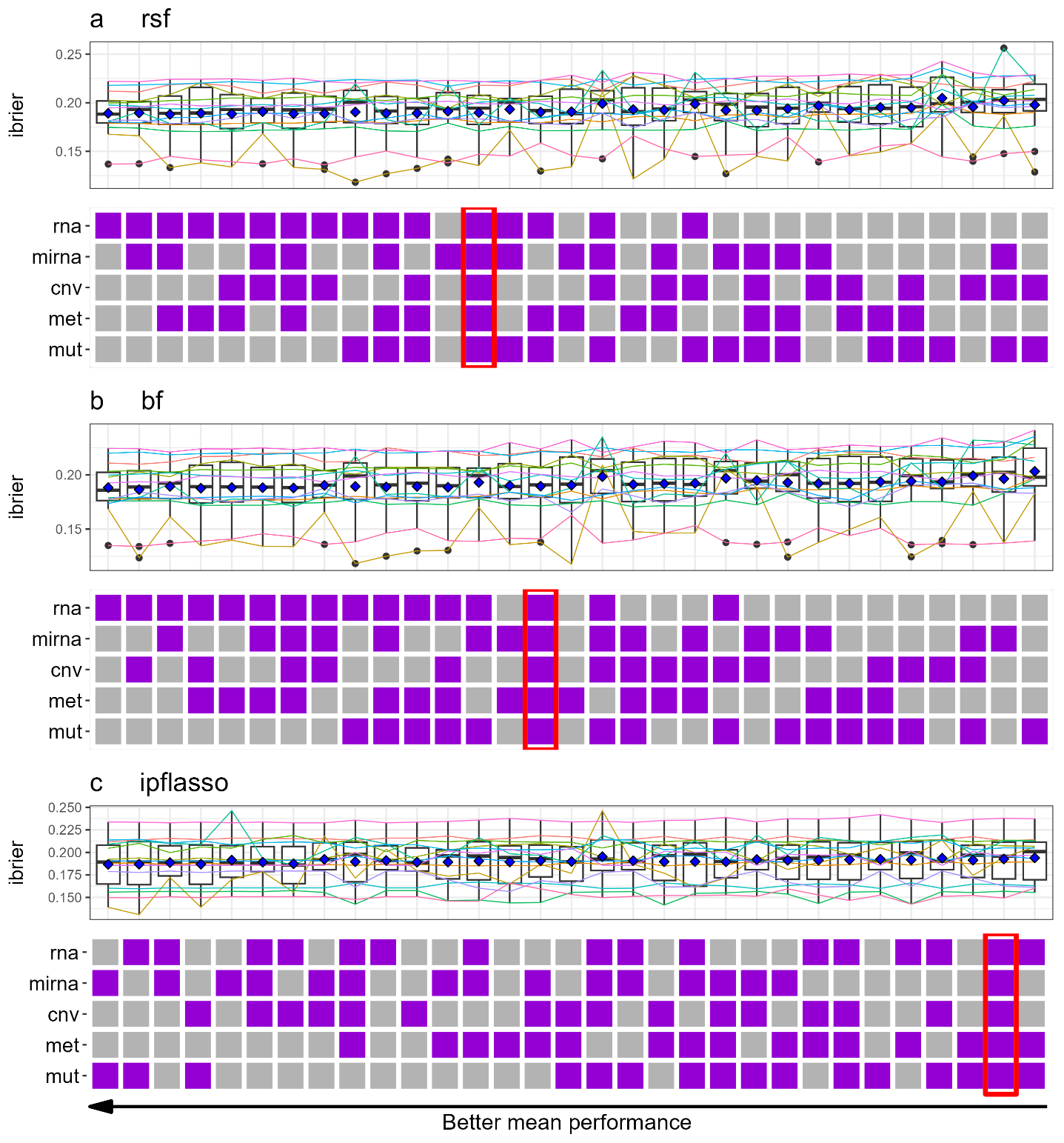


Figure S1: Dataset specific mean cross-validated ibrier values of each block combination. The purple squares indicate which omics block(s) were included in the respective combinations. The values shown by the boxplots are the ibrier values achieved across all 14 datasets, where the blue diamonds represent the means of the values. The upper (a), middle (b), and lower (c) panels show the results obtained for rsf, bf, and ipflasso, respectively. The combinations are sorted in increasing order according to the mean ranks across the datasets, which is why the combinations further to the left tend to perform better. cnv: CNV, mirna: miRNA, mut: DNAseq, met: methylation, rna: mRNA.


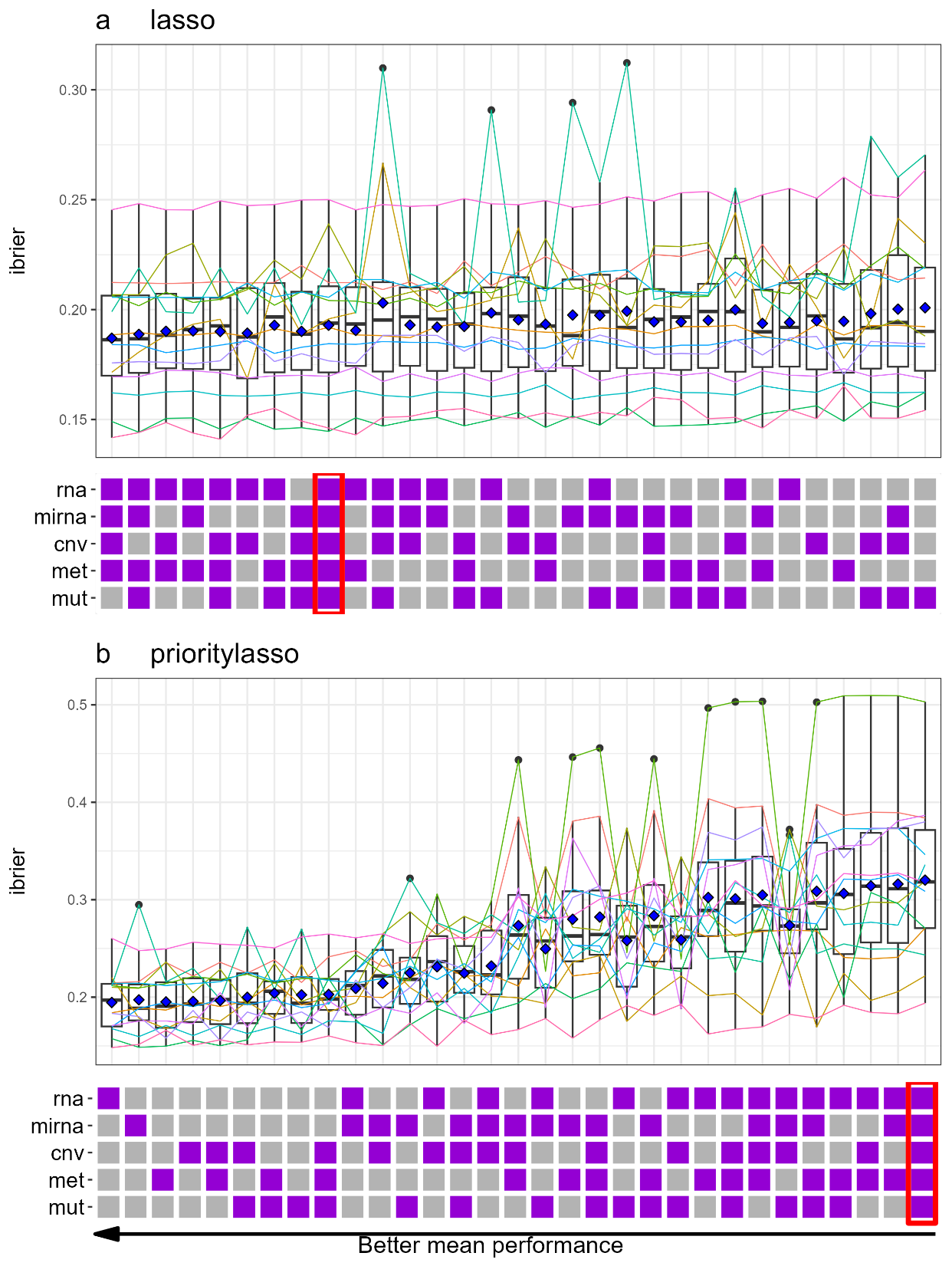


Figure S2: Dataset specific mean cross-validated ibrier values of each block combination. The purple squares indicate which omics block(s) were included in the respective combinations. The values shown by the boxplots are the ibrier values achieved across all 14 datasets, where the blue diamonds represent the means of the values. The upper (a) and lower (b) panels show the results obtained for lasso and prioritylasso, respectively. The combinations are sorted in increasing order according to the mean ranks across the datasets, which is why the combinations further to the left tend to perform better. cnv: CNV, mirna: miRNA, mut: DNAseq, met: methylation, rna: mRNA.


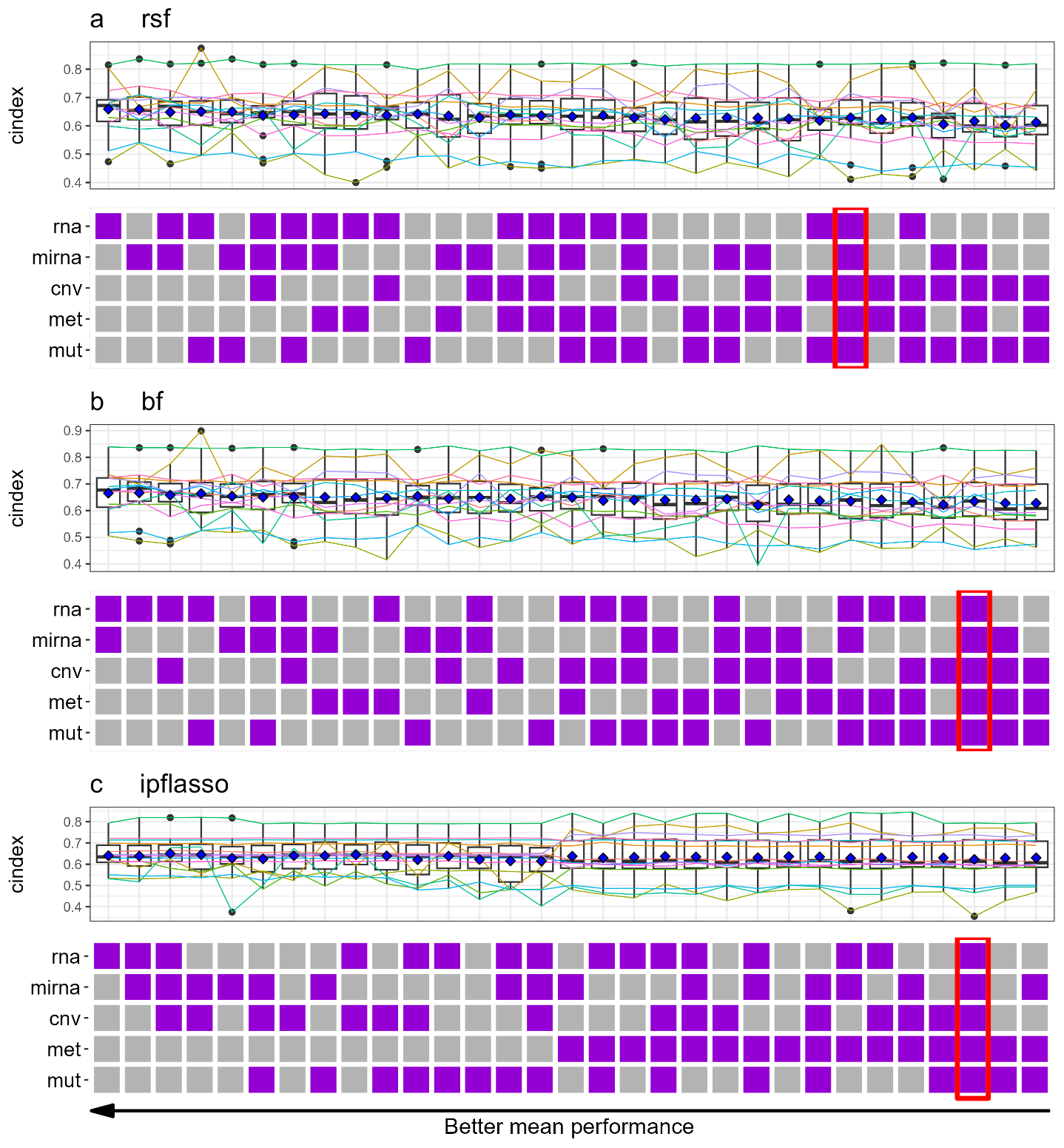


Figure S3: Dataset specific mean cross-validated cindex values of each block combination. The purple squares indicate which omics block(s) were included in the respective combinations. The values shown by the boxplots are the cindex values achieved across all 14 datasets, where the blue diamonds represent the means of the values. The upper (a), middle (b), and lower (c) panels show the results obtained for rsf, bf, and ipflasso, respectively. The combinations are sorted in increasing order according to the mean ranks across the datasets, which is why the combinations further to the left tend to perform better. cnv: CNV, mirna: miRNA, mut: DNAseq, met: methylation, rna: mRNA.

**
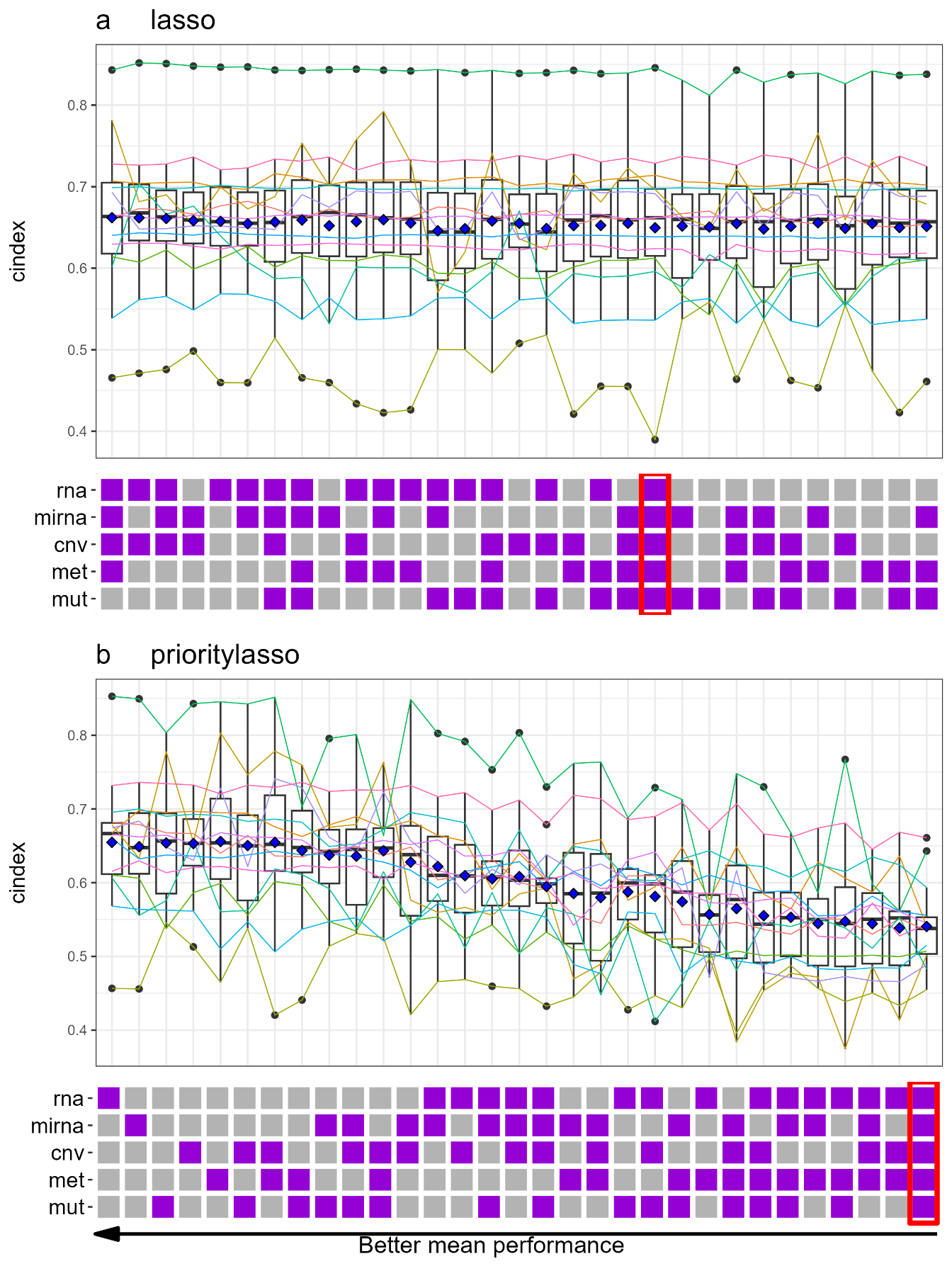
**

Figure S4: Dataset specific mean cross-validated cindex values of each block combination. The purple squares indicate which omics block(s) were included in the respective combinations. The values shown by the boxplots are the cindex values achieved across all 14 datasets, where the blue diamonds represent the means of the values. The upper (a) and lower (b) panels show the results obtained for lasso and prioritylasso, respectively. The combinations are sorted in increasing order according to the mean ranks across the datasets, which is why the combinations further to the left tend to perform better. cnv: CNV, mirna: miRNA, mut: DNAseq, met: methylation, rna: mRNA.

Table S2: Dataset specific mean cross-validated ibrier values for each combination of block combination, dataset, and prediction method. cnv: CNV, mirna: miRNA, mut: DNAseq, met: methylation, rna: mRNA

| Dataset | sMethod | 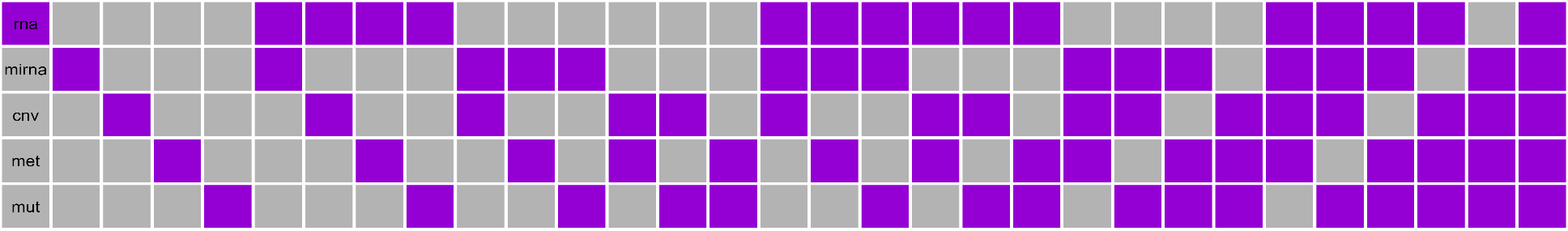 | | | | | | | | | | | | | | | | | | | | | | | | | | | | | | |
| --- | --- | --- | --- | --- | --- | --- | --- | --- | --- | --- | --- | --- | --- | --- | --- | --- | --- | --- | --- | --- | --- | --- | --- | --- | --- | --- | --- | --- | --- | --- | --- | --- |
| BLCA | bf | 0.21 | 0.21 | 0.21 | 0.22 | 0.22 | 0.21 | 0.21 | 0.22 | 0.21 | 0.21 | 0.22 | 0.21 | 0.22 | 0.22 | 0.22 | 0.21 | 0.22 | 0.22 | 0.22 | 0.21 | 0.22 | 0.22 | 0.22 | 0.22 | 0.22 | 0.22 | 0.22 | 0.22 | 0.22 | 0.22 | 0.22 |
|  | rsf | 0.21 | 0.21 | 0.22 | 0.23 | 0.22 | 0.21 | 0.21 | 0.22 | 0.22 | 0.21 | 0.22 | 0.22 | 0.22 | 0.22 | 0.23 | 0.21 | 0.22 | 0.22 | 0.22 | 0.21 | 0.22 | 0.22 | 0.22 | 0.23 | 0.22 | 0.21 | 0.21 | 0.22 | 0.22 | 0.22 | 0.22 |
|  | lasso | 0.21 | 0.22 | 0.22 | 0.23 | 0.21 | 0.21 | 0.21 | 0.21 | 0.21 | 0.22 | 0.23 | 0.22 | 0.22 | 0.22 | 0.23 | 0.21 | 0.21 | 0.21 | 0.21 | 0.21 | 0.21 | 0.23 | 0.21 | 0.22 | 0.22 | 0.21 | 0.21 | 0.21 | 0.21 | 0.22 | 0.21 |
|  | ipflasso | 0.22 | 0.22 | 0.22 | 0.22 | 0.21 | 0.22 | 0.22 | 0.21 | 0.21 | 0.22 | 0.22 | 0.21 | 0.22 | 0.21 | 0.22 | 0.22 | 0.21 | 0.21 | 0.21 | 0.21 | 0.21 | 0.22 | 0.21 | 0.22 | 0.22 | 0.21 | 0.21 | 0.21 | 0.21 | 0.22 | 0.21 |
|  | prioritylasso | 0.21 | 0.22 | 0.22 | 0.24 | 0.22 | 0.25 | 0.28 | 0.40 | 0.28 | 0.23 | 0.38 | 0.24 | 0.24 | 0.22 | 0.24 | 0.27 | 0.39 | 0.28 | 0.39 | 0.29 | 0.39 | 0.38 | 0.26 | 0.39 | 0.24 | 0.40 | 0.28 | 0.40 | 0.39 | 0.39 | 0.38 |
| BRCA | bf | 0.18 | 0.19 | 0.19 | 0.18 | 0.20 | 0.18 | 0.18 | 0.18 | 0.18 | 0.19 | 0.18 | 0.19 | 0.19 | 0.19 | 0.19 | 0.18 | 0.18 | 0.18 | 0.18 | 0.18 | 0.18 | 0.18 | 0.19 | 0.19 | 0.19 | 0.18 | 0.18 | 0.18 | 0.18 | 0.18 | 0.18 |
|  | rsf | 0.18 | 0.18 | 0.19 | 0.19 | 0.20 | 0.18 | 0.18 | 0.18 | 0.18 | 0.19 | 0.18 | 0.19 | 0.19 | 0.19 | 0.19 | 0.18 | 0.18 | 0.18 | 0.18 | 0.18 | 0.18 | 0.19 | 0.19 | 0.18 | 0.19 | 0.18 | 0.18 | 0.18 | 0.18 | 0.19 | 0.18 |
|  | lasso | 0.19 | 0.19 | 0.19 | 0.19 | 0.19 | 0.19 | 0.19 | 0.19 | 0.19 | 0.19 | 0.19 | 0.19 | 0.19 | 0.19 | 0.19 | 0.19 | 0.19 | 0.19 | 0.19 | 0.19 | 0.19 | 0.19 | 0.19 | 0.19 | 0.19 | 0.19 | 0.19 | 0.19 | 0.19 | 0.19 | 0.19 |
|  | ipflasso | 0.19 | 0.19 | 0.19 | 0.19 | 0.19 | 0.19 | 0.19 | 0.19 | 0.19 | 0.19 | 0.19 | 0.19 | 0.18 | 0.19 | 0.19 | 0.19 | 0.19 | 0.19 | 0.19 | 0.19 | 0.19 | 0.19 | 0.19 | 0.19 | 0.19 | 0.19 | 0.19 | 0.19 | 0.19 | 0.19 | 0.19 |
|  | prioritylasso | 0.18 | 0.19 | 0.20 | 0.19 | 0.19 | 0.20 | 0.23 | 0.26 | 0.28 | 0.20 | 0.22 | 0.22 | 0.19 | 0.20 | 0.22 | 0.22 | 0.24 | 0.27 | 0.24 | 0.27 | 0.24 | 0.21 | 0.22 | 0.23 | 0.20 | 0.27 | 0.27 | 0.27 | 0.26 | 0.23 | 0.27 |
| COAD | bf | 0.17 | 0.14 | 0.14 | 0.12 | 0.18 | 0.16 | 0.12 | 0.14 | 0.12 | 0.19 | 0.14 | 0.12 | 0.15 | 0.12 | 0.15 | 0.17 | 0.13 | 0.17 | 0.13 | 0.20 | 0.13 | 0.15 | 0.18 | 0.14 | 0.16 | 0.13 | 0.21 | 0.13 | 0.13 | 0.15 | 0.14 |
|  | rsf | 0.17 | 0.14 | 0.14 | 0.12 | 0.19 | 0.17 | 0.13 | 0.14 | 0.12 | 0.20 | 0.13 | 0.13 | 0.15 | 0.13 | 0.15 | 0.17 | 0.13 | 0.17 | 0.13 | 0.20 | 0.13 | 0.14 | 0.19 | 0.14 | 0.16 | 0.13 | 0.21 | 0.13 | 0.13 | 0.14 | 0.14 |
|  | lasso | 0.22 | 0.18 | 0.20 | 0.18 | 0.23 | 0.20 | 0.17 | 0.20 | 0.20 | 0.24 | 0.19 | 0.19 | 0.20 | 0.20 | 0.21 | 0.21 | 0.19 | 0.22 | 0.19 | 0.24 | 0.21 | 0.20 | 0.24 | 0.20 | 0.20 | 0.17 | 0.27 | 0.18 | 0.20 | 0.19 | 0.20 |
|  | ipflasso | 0.20 | 0.17 | 0.18 | 0.17 | 0.20 | 0.17 | 0.16 | 0.20 | 0.13 | 0.22 | 0.17 | 0.14 | 0.18 | 0.14 | 0.17 | 0.18 | 0.18 | 0.19 | 0.19 | 0.21 | 0.21 | 0.19 | 0.20 | 0.18 | 0.18 | 0.17 | 0.25 | 0.16 | 0.20 | 0.17 | 0.19 |
|  | prioritylasso | 0.22 | 0.18 | 0.18 | 0.17 | 0.23 | 0.19 | 0.19 | 0.20 | 0.18 | 0.24 | 0.24 | 0.21 | 0.18 | 0.20 | 0.18 | 0.20 | 0.21 | 0.19 | 0.20 | 0.22 | 0.22 | 0.20 | 0.22 | 0.20 | 0.18 | 0.18 | 0.26 | 0.17 | 0.20 | 0.24 | 0.22 |
| ESCA | bf | 0.20 | 0.20 | 0.20 | 0.22 | 0.20 | 0.20 | 0.20 | 0.21 | 0.20 | 0.20 | 0.21 | 0.20 | 0.22 | 0.20 | 0.22 | 0.20 | 0.21 | 0.20 | 0.21 | 0.20 | 0.21 | 0.22 | 0.20 | 0.22 | 0.22 | 0.21 | 0.21 | 0.21 | 0.21 | 0.21 | 0.21 |
|  | rsf | 0.20 | 0.19 | 0.21 | 0.23 | 0.20 | 0.20 | 0.21 | 0.22 | 0.20 | 0.21 | 0.22 | 0.20 | 0.22 | 0.20 | 0.23 | 0.20 | 0.21 | 0.20 | 0.21 | 0.21 | 0.21 | 0.22 | 0.20 | 0.22 | 0.22 | 0.21 | 0.21 | 0.21 | 0.20 | 0.21 | 0.21 |
|  | lasso | 0.21 | 0.21 | 0.20 | 0.23 | 0.19 | 0.21 | 0.21 | 0.22 | 0.21 | 0.21 | 0.22 | 0.19 | 0.23 | 0.19 | 0.23 | 0.21 | 0.23 | 0.21 | 0.22 | 0.21 | 0.22 | 0.23 | 0.20 | 0.23 | 0.22 | 0.21 | 0.20 | 0.21 | 0.20 | 0.21 | 0.24 |
|  | ipflasso | 0.19 | 0.19 | 0.20 | 0.21 | 0.19 | 0.19 | 0.19 | 0.20 | 0.19 | 0.19 | 0.20 | 0.19 | 0.21 | 0.19 | 0.21 | 0.19 | 0.21 | 0.19 | 0.21 | 0.19 | 0.21 | 0.21 | 0.19 | 0.21 | 0.21 | 0.20 | 0.19 | 0.20 | 0.20 | 0.20 | 0.21 |
|  | prioritylasso | 0.21 | 0.21 | 0.20 | 0.24 | 0.20 | 0.21 | 0.26 | 0.29 | 0.37 | 0.27 | 0.27 | 0.29 | 0.23 | 0.19 | 0.23 | 0.27 | 0.30 | 0.33 | 0.30 | 0.34 | 0.29 | 0.25 | 0.28 | 0.26 | 0.21 | 0.27 | 0.37 | 0.29 | 0.26 | 0.27 | 0.32 |
| HNSC | bf | 0.20 | 0.21 | 0.22 | 0.21 | 0.23 | 0.20 | 0.20 | 0.20 | 0.21 | 0.21 | 0.21 | 0.21 | 0.21 | 0.22 | 0.21 | 0.20 | 0.20 | 0.20 | 0.20 | 0.21 | 0.21 | 0.21 | 0.21 | 0.21 | 0.21 | 0.21 | 0.20 | 0.21 | 0.21 | 0.21 | 0.21 |
|  | rsf | 0.20 | 0.21 | 0.21 | 0.21 | 0.23 | 0.20 | 0.21 | 0.20 | 0.20 | 0.21 | 0.21 | 0.21 | 0.21 | 0.21 | 0.21 | 0.20 | 0.20 | 0.20 | 0.20 | 0.21 | 0.20 | 0.21 | 0.21 | 0.21 | 0.21 | 0.21 | 0.20 | 0.20 | 0.20 | 0.21 | 0.20 |
|  | lasso | 0.21 | 0.21 | 0.22 | 0.21 | 0.22 | 0.20 | 0.21 | 0.20 | 0.21 | 0.21 | 0.21 | 0.21 | 0.21 | 0.22 | 0.21 | 0.21 | 0.20 | 0.21 | 0.21 | 0.23 | 0.20 | 0.21 | 0.23 | 0.21 | 0.21 | 0.21 | 0.20 | 0.20 | 0.20 | 0.21 | 0.20 |
|  | ipflasso | 0.20 | 0.20 | 0.21 | 0.21 | 0.21 | 0.20 | 0.22 | 0.21 | 0.21 | 0.21 | 0.21 | 0.20 | 0.21 | 0.21 | 0.21 | 0.21 | 0.21 | 0.21 | 0.21 | 0.21 | 0.21 | 0.21 | 0.21 | 0.21 | 0.21 | 0.21 | 0.20 | 0.21 | 0.21 | 0.21 | 0.21 |
|  | prioritylasso | 0.21 | 0.21 | 0.22 | 0.21 | 0.22 | 0.24 | 0.31 | 0.50 | 0.25 | 0.22 | 0.45 | 0.22 | 0.22 | 0.22 | 0.21 | 0.30 | 0.51 | 0.23 | 0.51 | 0.24 | 0.51 | 0.44 | 0.23 | 0.44 | 0.22 | 0.50 | 0.25 | 0.50 | 0.50 | 0.46 | 0.50 |
| LGG | bf | 0.18 | 0.18 | 0.18 | 0.17 | 0.20 | 0.18 | 0.18 | 0.17 | 0.18 | 0.17 | 0.17 | 0.18 | 0.17 | 0.18 | 0.17 | 0.17 | 0.17 | 0.18 | 0.17 | 0.18 | 0.17 | 0.17 | 0.17 | 0.17 | 0.17 | 0.17 | 0.18 | 0.17 | 0.17 | 0.17 | 0.17 |
|  | rsf | 0.17 | 0.18 | 0.18 | 0.17 | 0.20 | 0.17 | 0.17 | 0.17 | 0.18 | 0.17 | 0.17 | 0.18 | 0.17 | 0.18 | 0.17 | 0.17 | 0.17 | 0.18 | 0.17 | 0.17 | 0.17 | 0.17 | 0.17 | 0.17 | 0.17 | 0.17 | 0.17 | 0.17 | 0.17 | 0.17 | 0.17 |
|  | lasso | 0.15 | 0.15 | 0.16 | 0.15 | 0.16 | 0.15 | 0.15 | 0.15 | 0.15 | 0.15 | 0.15 | 0.16 | 0.15 | 0.16 | 0.15 | 0.15 | 0.15 | 0.15 | 0.15 | 0.15 | 0.15 | 0.15 | 0.16 | 0.15 | 0.15 | 0.15 | 0.15 | 0.14 | 0.15 | 0.15 | 0.14 |
|  | ipflasso | 0.16 | 0.16 | 0.16 | 0.14 | 0.16 | 0.16 | 0.16 | 0.14 | 0.16 | 0.16 | 0.15 | 0.16 | 0.14 | 0.16 | 0.16 | 0.16 | 0.15 | 0.16 | 0.14 | 0.16 | 0.16 | 0.14 | 0.16 | 0.15 | 0.16 | 0.14 | 0.16 | 0.15 | 0.16 | 0.16 | 0.16 |
|  | prioritylasso | 0.16 | 0.15 | 0.16 | 0.15 | 0.17 | 0.18 | 0.19 | 0.30 | 0.24 | 0.15 | 0.20 | 0.17 | 0.15 | 0.16 | 0.20 | 0.19 | 0.30 | 0.21 | 0.31 | 0.23 | 0.20 | 0.19 | 0.18 | 0.23 | 0.22 | 0.29 | 0.22 | 0.28 | 0.23 | 0.21 | 0.27 |
| LIHC | bf | 0.18 | 0.23 | 0.19 | 0.19 | 0.23 | 0.19 | 0.18 | 0.18 | 0.22 | 0.18 | 0.18 | 0.21 | 0.18 | 0.21 | 0.19 | 0.18 | 0.18 | 0.21 | 0.17 | 0.22 | 0.18 | 0.18 | 0.23 | 0.19 | 0.17 | 0.17 | 0.23 | 0.18 | 0.18 | 0.17 | 0.18 |
|  | rsf | 0.18 | 0.22 | 0.19 | 0.19 | 0.23 | 0.19 | 0.18 | 0.18 | 0.22 | 0.18 | 0.19 | 0.21 | 0.18 | 0.22 | 0.19 | 0.18 | 0.18 | 0.20 | 0.17 | 0.23 | 0.18 | 0.18 | 0.26 | 0.19 | 0.17 | 0.17 | 0.23 | 0.18 | 0.18 | 0.17 | 0.18 |
|  | lasso | 0.20 | 0.29 | 0.22 | 0.21 | 0.27 | 0.21 | 0.20 | 0.20 | 0.29 | 0.20 | 0.21 | 0.31 | 0.20 | 0.28 | 0.21 | 0.22 | 0.20 | 0.26 | 0.20 | 0.26 | 0.22 | 0.20 | 0.26 | 0.21 | 0.19 | 0.20 | 0.31 | 0.22 | 0.22 | 0.19 | 0.22 |
|  | ipflasso | 0.21 | 0.25 | 0.19 | 0.20 | 0.21 | 0.21 | 0.19 | 0.22 | 0.21 | 0.20 | 0.20 | 0.21 | 0.20 | 0.21 | 0.20 | 0.19 | 0.22 | 0.21 | 0.22 | 0.22 | 0.21 | 0.20 | 0.22 | 0.20 | 0.19 | 0.22 | 0.22 | 0.21 | 0.21 | 0.19 | 0.21 |
|  | prioritylasso | 0.21 | 0.29 | 0.23 | 0.22 | 0.27 | 0.23 | 0.28 | 0.24 | 0.29 | 0.26 | 0.25 | 0.32 | 0.20 | 0.27 | 0.21 | 0.28 | 0.25 | 0.29 | 0.25 | 0.28 | 0.25 | 0.27 | 0.27 | 0.26 | 0.19 | 0.24 | 0.37 | 0.24 | 0.24 | 0.25 | 0.24 |
| LUAD | bf | 0.20 | 0.22 | 0.21 | 0.19 | 0.21 | 0.20 | 0.20 | 0.20 | 0.20 | 0.21 | 0.19 | 0.21 | 0.20 | 0.21 | 0.20 | 0.20 | 0.20 | 0.20 | 0.20 | 0.21 | 0.20 | 0.20 | 0.21 | 0.20 | 0.20 | 0.20 | 0.21 | 0.20 | 0.20 | 0.20 | 0.20 |
|  | rsf | 0.20 | 0.19 | 0.20 | 0.19 | 0.21 | 0.20 | 0.20 | 0.19 | 0.20 | 0.20 | 0.19 | 0.21 | 0.19 | 0.21 | 0.20 | 0.20 | 0.19 | 0.20 | 0.20 | 0.21 | 0.20 | 0.19 | 0.21 | 0.20 | 0.20 | 0.20 | 0.20 | 0.20 | 0.20 | 0.20 | 0.20 |
|  | lasso | 0.16 | 0.16 | 0.16 | 0.17 | 0.16 | 0.16 | 0.16 | 0.16 | 0.16 | 0.16 | 0.17 | 0.16 | 0.17 | 0.16 | 0.16 | 0.16 | 0.16 | 0.16 | 0.16 | 0.16 | 0.16 | 0.16 | 0.16 | 0.16 | 0.16 | 0.16 | 0.16 | 0.16 | 0.16 | 0.16 | 0.16 |
|  | ipflasso | 0.16 | 0.16 | 0.16 | 0.17 | 0.16 | 0.16 | 0.16 | 0.17 | 0.16 | 0.16 | 0.17 | 0.16 | 0.17 | 0.16 | 0.16 | 0.16 | 0.17 | 0.16 | 0.17 | 0.16 | 0.16 | 0.17 | 0.16 | 0.16 | 0.16 | 0.17 | 0.16 | 0.16 | 0.16 | 0.16 | 0.16 |
|  | prioritylasso | 0.17 | 0.16 | 0.16 | 0.17 | 0.16 | 0.17 | 0.19 | 0.27 | 0.29 | 0.16 | 0.24 | 0.24 | 0.17 | 0.16 | 0.17 | 0.18 | 0.27 | 0.31 | 0.28 | 0.31 | 0.27 | 0.24 | 0.21 | 0.28 | 0.18 | 0.30 | 0.29 | 0.33 | 0.32 | 0.26 | 0.34 |
| LUSC | bf | 0.22 | 0.23 | 0.23 | 0.22 | 0.24 | 0.22 | 0.22 | 0.22 | 0.22 | 0.22 | 0.22 | 0.22 | 0.22 | 0.23 | 0.22 | 0.22 | 0.22 | 0.22 | 0.22 | 0.22 | 0.22 | 0.22 | 0.23 | 0.22 | 0.22 | 0.22 | 0.23 | 0.22 | 0.22 | 0.22 | 0.22 |
|  | rsf | 0.22 | 0.22 | 0.23 | 0.22 | 0.24 | 0.22 | 0.22 | 0.22 | 0.22 | 0.22 | 0.22 | 0.23 | 0.22 | 0.23 | 0.22 | 0.22 | 0.22 | 0.22 | 0.22 | 0.22 | 0.22 | 0.22 | 0.23 | 0.22 | 0.23 | 0.22 | 0.22 | 0.22 | 0.22 | 0.22 | 0.22 |
|  | lasso | 0.21 | 0.21 | 0.21 | 0.21 | 0.22 | 0.21 | 0.21 | 0.21 | 0.22 | 0.22 | 0.21 | 0.22 | 0.21 | 0.22 | 0.21 | 0.21 | 0.21 | 0.22 | 0.21 | 0.22 | 0.21 | 0.21 | 0.21 | 0.21 | 0.21 | 0.21 | 0.21 | 0.21 | 0.21 | 0.21 | 0.21 |
|  | ipflasso | 0.21 | 0.21 | 0.21 | 0.20 | 0.21 | 0.21 | 0.21 | 0.21 | 0.21 | 0.21 | 0.20 | 0.21 | 0.20 | 0.21 | 0.20 | 0.21 | 0.20 | 0.21 | 0.20 | 0.21 | 0.20 | 0.20 | 0.21 | 0.20 | 0.20 | 0.20 | 0.21 | 0.20 | 0.21 | 0.20 | 0.21 |
|  | prioritylasso | 0.21 | 0.21 | 0.21 | 0.21 | 0.22 | 0.21 | 0.22 | 0.34 | 0.30 | 0.22 | 0.31 | 0.23 | 0.22 | 0.23 | 0.22 | 0.22 | 0.37 | 0.28 | 0.37 | 0.28 | 0.37 | 0.29 | 0.23 | 0.29 | 0.22 | 0.34 | 0.33 | 0.36 | 0.34 | 0.30 | 0.35 |
| PAAD | bf | 0.17 | 0.19 | 0.19 | 0.18 | 0.19 | 0.18 | 0.18 | 0.18 | 0.17 | 0.19 | 0.18 | 0.19 | 0.19 | 0.19 | 0.18 | 0.18 | 0.18 | 0.18 | 0.18 | 0.18 | 0.18 | 0.19 | 0.19 | 0.18 | 0.19 | 0.18 | 0.18 | 0.18 | 0.18 | 0.19 | 0.18 |
|  | rsf | 0.18 | 0.18 | 0.20 | 0.19 | 0.19 | 0.18 | 0.19 | 0.19 | 0.18 | 0.20 | 0.19 | 0.18 | 0.19 | 0.20 | 0.19 | 0.19 | 0.18 | 0.18 | 0.19 | 0.19 | 0.18 | 0.19 | 0.20 | 0.19 | 0.19 | 0.19 | 0.19 | 0.18 | 0.19 | 0.19 | 0.19 |
|  | lasso | 0.19 | 0.19 | 0.18 | 0.18 | 0.18 | 0.19 | 0.19 | 0.18 | 0.19 | 0.18 | 0.19 | 0.18 | 0.18 | 0.18 | 0.18 | 0.19 | 0.18 | 0.19 | 0.18 | 0.19 | 0.18 | 0.18 | 0.18 | 0.18 | 0.18 | 0.18 | 0.19 | 0.18 | 0.18 | 0.18 | 0.18 |
|  | ipflasso | 0.19 | 0.19 | 0.19 | 0.19 | 0.19 | 0.19 | 0.19 | 0.20 | 0.19 | 0.19 | 0.20 | 0.19 | 0.20 | 0.19 | 0.20 | 0.19 | 0.19 | 0.19 | 0.19 | 0.19 | 0.19 | 0.19 | 0.19 | 0.19 | 0.19 | 0.20 | 0.19 | 0.20 | 0.20 | 0.19 | 0.20 |
|  | prioritylasso | 0.17 | 0.19 | 0.19 | 0.19 | 0.19 | 0.23 | 0.24 | 0.29 | 0.20 | 0.25 | 0.25 | 0.19 | 0.20 | 0.19 | 0.20 | 0.22 | 0.33 | 0.21 | 0.32 | 0.25 | 0.32 | 0.26 | 0.20 | 0.26 | 0.19 | 0.29 | 0.28 | 0.28 | 0.28 | 0.25 | 0.31 |
| SARC | bf | 0.18 | 0.18 | 0.19 | 0.17 | 0.18 | 0.18 | 0.18 | 0.17 | 0.18 | 0.19 | 0.18 | 0.18 | 0.17 | 0.19 | 0.17 | 0.19 | 0.18 | 0.18 | 0.18 | 0.18 | 0.18 | 0.18 | 0.19 | 0.18 | 0.18 | 0.18 | 0.19 | 0.18 | 0.18 | 0.18 | 0.18 |
|  | rsf | 0.18 | 0.18 | 0.20 | 0.17 | 0.19 | 0.18 | 0.19 | 0.18 | 0.18 | 0.20 | 0.18 | 0.18 | 0.18 | 0.20 | 0.18 | 0.19 | 0.18 | 0.18 | 0.18 | 0.19 | 0.18 | 0.18 | 0.19 | 0.18 | 0.18 | 0.18 | 0.19 | 0.18 | 0.18 | 0.18 | 0.18 |
|  | lasso | 0.19 | 0.18 | 0.19 | 0.17 | 0.18 | 0.19 | 0.19 | 0.18 | 0.19 | 0.19 | 0.18 | 0.19 | 0.17 | 0.19 | 0.18 | 0.19 | 0.18 | 0.19 | 0.18 | 0.19 | 0.18 | 0.18 | 0.18 | 0.18 | 0.18 | 0.18 | 0.19 | 0.18 | 0.18 | 0.18 | 0.18 |
|  | ipflasso | 0.18 | 0.18 | 0.18 | 0.16 | 0.18 | 0.18 | 0.18 | 0.16 | 0.18 | 0.18 | 0.17 | 0.18 | 0.16 | 0.18 | 0.17 | 0.18 | 0.16 | 0.18 | 0.16 | 0.18 | 0.16 | 0.17 | 0.18 | 0.17 | 0.17 | 0.16 | 0.18 | 0.16 | 0.16 | 0.17 | 0.16 |
|  | prioritylasso | 0.18 | 0.18 | 0.19 | 0.16 | 0.18 | 0.22 | 0.24 | 0.37 | 0.24 | 0.19 | 0.30 | 0.21 | 0.16 | 0.18 | 0.18 | 0.26 | 0.37 | 0.25 | 0.37 | 0.25 | 0.34 | 0.31 | 0.23 | 0.30 | 0.17 | 0.37 | 0.24 | 0.38 | 0.36 | 0.31 | 0.38 |
| SKCM | bf | 0.19 | 0.20 | 0.20 | 0.20 | 0.19 | 0.19 | 0.19 | 0.20 | 0.20 | 0.20 | 0.20 | 0.20 | 0.20 | 0.20 | 0.20 | 0.19 | 0.20 | 0.20 | 0.20 | 0.20 | 0.20 | 0.20 | 0.20 | 0.20 | 0.20 | 0.20 | 0.20 | 0.20 | 0.20 | 0.20 | 0.20 |
|  | rsf | 0.19 | 0.20 | 0.20 | 0.20 | 0.19 | 0.20 | 0.20 | 0.20 | 0.20 | 0.20 | 0.20 | 0.20 | 0.20 | 0.20 | 0.20 | 0.20 | 0.20 | 0.20 | 0.20 | 0.20 | 0.20 | 0.20 | 0.20 | 0.20 | 0.20 | 0.20 | 0.20 | 0.20 | 0.20 | 0.20 | 0.20 |
|  | lasso | 0.17 | 0.17 | 0.17 | 0.17 | 0.17 | 0.17 | 0.17 | 0.17 | 0.17 | 0.17 | 0.17 | 0.17 | 0.17 | 0.17 | 0.17 | 0.17 | 0.17 | 0.17 | 0.17 | 0.17 | 0.17 | 0.17 | 0.17 | 0.17 | 0.17 | 0.17 | 0.17 | 0.17 | 0.17 | 0.17 | 0.17 |
|  | ipflasso | 0.19 | 0.19 | 0.19 | 0.20 | 0.19 | 0.19 | 0.19 | 0.20 | 0.19 | 0.19 | 0.20 | 0.19 | 0.20 | 0.19 | 0.20 | 0.19 | 0.20 | 0.19 | 0.20 | 0.19 | 0.20 | 0.20 | 0.19 | 0.20 | 0.20 | 0.20 | 0.19 | 0.20 | 0.20 | 0.20 | 0.20 |
|  | prioritylasso | 0.17 | 0.18 | 0.17 | 0.18 | 0.17 | 0.18 | 0.20 | 0.33 | 0.20 | 0.19 | 0.36 | 0.18 | 0.20 | 0.17 | 0.20 | 0.21 | 0.38 | 0.19 | 0.36 | 0.19 | 0.36 | 0.31 | 0.17 | 0.32 | 0.20 | 0.35 | 0.21 | 0.35 | 0.34 | 0.31 | 0.39 |
| STAD | bf | 0.22 | 0.23 | 0.23 | 0.23 | 0.24 | 0.22 | 0.22 | 0.22 | 0.22 | 0.23 | 0.23 | 0.22 | 0.23 | 0.23 | 0.23 | 0.22 | 0.22 | 0.22 | 0.22 | 0.22 | 0.22 | 0.23 | 0.23 | 0.22 | 0.23 | 0.22 | 0.22 | 0.22 | 0.22 | 0.23 | 0.22 |
|  | rsf | 0.22 | 0.22 | 0.23 | 0.23 | 0.24 | 0.22 | 0.22 | 0.22 | 0.22 | 0.23 | 0.23 | 0.22 | 0.23 | 0.23 | 0.23 | 0.22 | 0.22 | 0.22 | 0.22 | 0.22 | 0.22 | 0.23 | 0.23 | 0.23 | 0.23 | 0.23 | 0.22 | 0.22 | 0.22 | 0.23 | 0.22 |
|  | lasso | 0.26 | 0.25 | 0.25 | 0.26 | 0.26 | 0.25 | 0.25 | 0.25 | 0.25 | 0.25 | 0.25 | 0.25 | 0.25 | 0.25 | 0.25 | 0.25 | 0.25 | 0.25 | 0.25 | 0.25 | 0.25 | 0.25 | 0.25 | 0.25 | 0.25 | 0.25 | 0.25 | 0.25 | 0.25 | 0.25 | 0.25 |
|  | ipflasso | 0.23 | 0.23 | 0.23 | 0.24 | 0.24 | 0.23 | 0.23 | 0.24 | 0.23 | 0.23 | 0.23 | 0.23 | 0.24 | 0.23 | 0.24 | 0.23 | 0.24 | 0.23 | 0.24 | 0.23 | 0.24 | 0.24 | 0.23 | 0.24 | 0.23 | 0.24 | 0.23 | 0.24 | 0.24 | 0.24 | 0.24 |
|  | prioritylasso | 0.26 | 0.25 | 0.26 | 0.25 | 0.26 | 0.26 | 0.26 | 0.28 | 0.31 | 0.26 | 0.28 | 0.26 | 0.25 | 0.25 | 0.25 | 0.26 | 0.33 | 0.28 | 0.32 | 0.28 | 0.33 | 0.30 | 0.26 | 0.32 | 0.26 | 0.29 | 0.29 | 0.30 | 0.32 | 0.30 | 0.32 |
| UCEC | bf | 0.14 | 0.14 | 0.14 | 0.16 | 0.14 | 0.14 | 0.13 | 0.14 | 0.14 | 0.14 | 0.14 | 0.14 | 0.15 | 0.14 | 0.14 | 0.14 | 0.15 | 0.14 | 0.14 | 0.14 | 0.15 | 0.15 | 0.14 | 0.15 | 0.15 | 0.14 | 0.14 | 0.15 | 0.14 | 0.14 | 0.14 |
|  | rsf | 0.14 | 0.14 | 0.14 | 0.17 | 0.14 | 0.14 | 0.14 | 0.14 | 0.14 | 0.14 | 0.15 | 0.15 | 0.15 | 0.15 | 0.16 | 0.14 | 0.14 | 0.15 | 0.14 | 0.14 | 0.16 | 0.15 | 0.15 | 0.16 | 0.16 | 0.14 | 0.14 | 0.15 | 0.14 | 0.15 | 0.15 |
|  | lasso | 0.15 | 0.15 | 0.15 | 0.17 | 0.15 | 0.15 | 0.15 | 0.14 | 0.15 | 0.15 | 0.15 | 0.15 | 0.15 | 0.15 | 0.15 | 0.15 | 0.14 | 0.15 | 0.15 | 0.15 | 0.16 | 0.16 | 0.15 | 0.16 | 0.15 | 0.14 | 0.15 | 0.14 | 0.14 | 0.15 | 0.15 |
|  | ipflasso | 0.15 | 0.15 | 0.15 | 0.17 | 0.15 | 0.15 | 0.15 | 0.14 | 0.15 | 0.15 | 0.15 | 0.15 | 0.15 | 0.15 | 0.15 | 0.15 | 0.15 | 0.15 | 0.15 | 0.15 | 0.16 | 0.16 | 0.15 | 0.16 | 0.15 | 0.15 | 0.15 | 0.15 | 0.15 | 0.15 | 0.15 |
|  | prioritylasso | 0.15 | 0.15 | 0.15 | 0.17 | 0.15 | 0.15 | 0.15 | 0.16 | 0.19 | 0.15 | 0.16 | 0.17 | 0.16 | 0.15 | 0.15 | 0.16 | 0.18 | 0.18 | 0.18 | 0.19 | 0.19 | 0.17 | 0.18 | 0.18 | 0.16 | 0.17 | 0.18 | 0.18 | 0.17 | 0.18 | 0.19 |

Table S3: Dataset specific mean cross-validated cindex values for each combination of block combination, dataset, and prediction method. cnv: CNV, mirna: miRNA, mut: DNAseq, met: methylation, rna: mRNA.

| Dataset | Method | 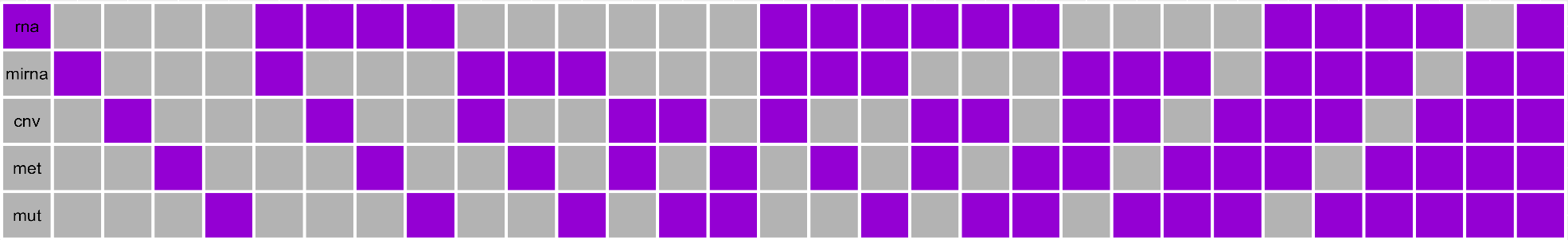 | | | | | | | | | | | | | | | | | | | | | | | | | | | | | | |
| --- | --- | --- | --- | --- | --- | --- | --- | --- | --- | --- | --- | --- | --- | --- | --- | --- | --- | --- | --- | --- | --- | --- | --- | --- | --- | --- | --- | --- | --- | --- | --- | --- |
| BLCA | bf | 0.67 | 0.66 | 0.63 | 0.60 | 0.65 | 0.67 | 0.67 | 0.62 | 0.66 | 0.65 | 0.60 | 0.66 | 0.59 | 0.62 | 0.58 | 0.67 | 0.61 | 0.65 | 0.62 | 0.66 | 0.60 | 0.59 | 0.64 | 0.56 | 0.56 | 0.60 | 0.65 | 0.58 | 0.59 | 0.56 | 0.59 |
|  | rsf | 0.66 | 0.65 | 0.62 | 0.55 | 0.63 | 0.67 | 0.67 | 0.60 | 0.65 | 0.65 | 0.56 | 0.64 | 0.57 | 0.61 | 0.54 | 0.67 | 0.61 | 0.65 | 0.62 | 0.66 | 0.59 | 0.59 | 0.64 | 0.56 | 0.56 | 0.62 | 0.66 | 0.60 | 0.61 | 0.57 | 0.61 |
|  | lasso | 0.68 | 0.67 | 0.67 | 0.65 | 0.67 | 0.68 | 0.67 | 0.66 | 0.67 | 0.67 | 0.65 | 0.67 | 0.65 | 0.66 | 0.65 | 0.67 | 0.66 | 0.68 | 0.66 | 0.67 | 0.66 | 0.65 | 0.67 | 0.65 | 0.66 | 0.66 | 0.67 | 0.66 | 0.66 | 0.66 | 0.66 |
|  | ipflasso | 0.66 | 0.66 | 0.66 | 0.62 | 0.65 | 0.66 | 0.66 | 0.62 | 0.65 | 0.66 | 0.62 | 0.65 | 0.61 | 0.65 | 0.61 | 0.66 | 0.62 | 0.65 | 0.62 | 0.65 | 0.62 | 0.61 | 0.65 | 0.61 | 0.61 | 0.63 | 0.65 | 0.63 | 0.63 | 0.61 | 0.63 |
|  | prioritylasso | 0.68 | 0.68 | 0.67 | 0.64 | 0.67 | 0.60 | 0.61 | 0.54 | 0.61 | 0.65 | 0.56 | 0.65 | 0.64 | 0.66 | 0.64 | 0.60 | 0.55 | 0.63 | 0.55 | 0.61 | 0.55 | 0.54 | 0.65 | 0.54 | 0.64 | 0.53 | 0.59 | 0.53 | 0.54 | 0.55 | 0.54 |
| BRCA | bf | 0.72 | 0.71 | 0.70 | 0.71 | 0.69 | 0.72 | 0.71 | 0.71 | 0.71 | 0.71 | 0.71 | 0.70 | 0.69 | 0.70 | 0.70 | 0.71 | 0.71 | 0.72 | 0.71 | 0.71 | 0.70 | 0.69 | 0.71 | 0.70 | 0.69 | 0.71 | 0.71 | 0.71 | 0.71 | 0.69 | 0.71 |
|  | rsf | 0.68 | 0.71 | 0.69 | 0.68 | 0.69 | 0.68 | 0.67 | 0.67 | 0.68 | 0.68 | 0.68 | 0.70 | 0.67 | 0.68 | 0.68 | 0.68 | 0.67 | 0.68 | 0.67 | 0.66 | 0.66 | 0.67 | 0.68 | 0.67 | 0.66 | 0.67 | 0.67 | 0.66 | 0.65 | 0.65 | 0.66 |
|  | lasso | 0.70 | 0.70 | 0.70 | 0.70 | 0.70 | 0.70 | 0.70 | 0.71 | 0.71 | 0.71 | 0.71 | 0.71 | 0.70 | 0.71 | 0.70 | 0.71 | 0.71 | 0.71 | 0.71 | 0.70 | 0.71 | 0.70 | 0.70 | 0.70 | 0.70 | 0.71 | 0.72 | 0.71 | 0.71 | 0.71 | 0.71 |
|  | ipflasso | 0.70 | 0.69 | 0.70 | 0.69 | 0.69 | 0.70 | 0.70 | 0.69 | 0.70 | 0.70 | 0.69 | 0.69 | 0.69 | 0.69 | 0.69 | 0.70 | 0.68 | 0.70 | 0.68 | 0.70 | 0.70 | 0.69 | 0.70 | 0.69 | 0.69 | 0.68 | 0.69 | 0.68 | 0.69 | 0.69 | 0.69 |
|  | prioritylasso | 0.67 | 0.70 | 0.70 | 0.69 | 0.70 | 0.66 | 0.66 | 0.58 | 0.58 | 0.68 | 0.65 | 0.68 | 0.68 | 0.69 | 0.66 | 0.64 | 0.63 | 0.59 | 0.64 | 0.59 | 0.61 | 0.66 | 0.69 | 0.65 | 0.67 | 0.59 | 0.60 | 0.56 | 0.58 | 0.65 | 0.54 |
| COAD | bf | 0.70 | 0.66 | 0.77 | 0.80 | 0.83 | 0.74 | 0.78 | 0.81 | 0.90 | 0.73 | 0.80 | 0.70 | 0.83 | 0.64 | 0.82 | 0.72 | 0.81 | 0.76 | 0.80 | 0.68 | 0.85 | 0.81 | 0.71 | 0.80 | 0.76 | 0.76 | 0.77 | 0.73 | 0.72 | 0.73 | 0.76 |
|  | rsf | 0.80 | 0.67 | 0.70 | 0.75 | 0.74 | 0.69 | 0.69 | 0.79 | 0.87 | 0.69 | 0.79 | 0.69 | 0.80 | 0.56 | 0.80 | 0.63 | 0.81 | 0.73 | 0.80 | 0.63 | 0.81 | 0.80 | 0.64 | 0.78 | 0.73 | 0.76 | 0.76 | 0.75 | 0.81 | 0.73 | 0.76 |
|  | lasso | 0.70 | 0.70 | 0.64 | 0.73 | 0.65 | 0.67 | 0.68 | 0.73 | 0.62 | 0.65 | 0.77 | 0.66 | 0.72 | 0.66 | 0.69 | 0.69 | 0.79 | 0.57 | 0.76 | 0.62 | 0.68 | 0.72 | 0.67 | 0.68 | 0.69 | 0.78 | 0.69 | 0.75 | 0.74 | 0.72 | 0.70 |
|  | ipflasso | 0.62 | 0.61 | 0.52 | 0.75 | 0.63 | 0.58 | 0.59 | 0.78 | 0.70 | 0.56 | 0.77 | 0.71 | 0.78 | 0.71 | 0.77 | 0.62 | 0.79 | 0.48 | 0.75 | 0.54 | 0.73 | 0.73 | 0.56 | 0.74 | 0.74 | 0.77 | 0.59 | 0.75 | 0.79 | 0.77 | 0.77 |
|  | prioritylasso | 0.68 | 0.63 | 0.66 | 0.80 | 0.78 | 0.56 | 0.57 | 0.51 | 0.55 | 0.58 | 0.47 | 0.68 | 0.78 | 0.75 | 0.76 | 0.58 | 0.47 | 0.56 | 0.41 | 0.52 | 0.37 | 0.49 | 0.68 | 0.52 | 0.76 | 0.49 | 0.59 | 0.48 | 0.45 | 0.38 | 0.50 |
| ESCA | bf | 0.49 | 0.52 | 0.49 | 0.46 | 0.55 | 0.51 | 0.48 | 0.41 | 0.53 | 0.51 | 0.48 | 0.55 | 0.44 | 0.54 | 0.43 | 0.47 | 0.46 | 0.53 | 0.47 | 0.52 | 0.46 | 0.47 | 0.53 | 0.49 | 0.46 | 0.46 | 0.50 | 0.49 | 0.46 | 0.49 | 0.46 |
|  | rsf | 0.47 | 0.54 | 0.47 | 0.42 | 0.57 | 0.47 | 0.45 | 0.40 | 0.49 | 0.49 | 0.45 | 0.58 | 0.43 | 0.52 | 0.43 | 0.47 | 0.43 | 0.50 | 0.46 | 0.50 | 0.47 | 0.45 | 0.51 | 0.47 | 0.44 | 0.45 | 0.48 | 0.46 | 0.42 | 0.44 | 0.41 |
|  | lasso | 0.46 | 0.46 | 0.51 | 0.47 | 0.56 | 0.46 | 0.47 | 0.43 | 0.50 | 0.50 | 0.45 | 0.54 | 0.42 | 0.55 | 0.42 | 0.48 | 0.42 | 0.50 | 0.43 | 0.52 | 0.46 | 0.46 | 0.54 | 0.46 | 0.46 | 0.47 | 0.51 | 0.47 | 0.47 | 0.46 | 0.39 |
|  | ipflasso | 0.53 | 0.53 | 0.54 | 0.48 | 0.57 | 0.53 | 0.53 | 0.44 | 0.57 | 0.54 | 0.48 | 0.56 | 0.43 | 0.56 | 0.43 | 0.53 | 0.38 | 0.57 | 0.43 | 0.57 | 0.46 | 0.47 | 0.57 | 0.47 | 0.47 | 0.47 | 0.56 | 0.47 | 0.51 | 0.48 | 0.36 |
|  | prioritylasso | 0.46 | 0.46 | 0.51 | 0.46 | 0.55 | 0.47 | 0.47 | 0.50 | 0.43 | 0.42 | 0.44 | 0.51 | 0.42 | 0.54 | 0.44 | 0.46 | 0.46 | 0.46 | 0.43 | 0.45 | 0.44 | 0.48 | 0.53 | 0.43 | 0.53 | 0.45 | 0.43 | 0.49 | 0.46 | 0.40 | 0.45 |
| HNSC | bf | 0.62 | 0.61 | 0.60 | 0.59 | 0.55 | 0.62 | 0.62 | 0.60 | 0.58 | 0.62 | 0.60 | 0.58 | 0.59 | 0.58 | 0.58 | 0.62 | 0.60 | 0.60 | 0.60 | 0.60 | 0.58 | 0.59 | 0.61 | 0.58 | 0.58 | 0.60 | 0.60 | 0.58 | 0.58 | 0.58 | 0.58 |
|  | rsf | 0.63 | 0.62 | 0.61 | 0.58 | 0.57 | 0.63 | 0.62 | 0.60 | 0.60 | 0.62 | 0.59 | 0.59 | 0.59 | 0.59 | 0.58 | 0.63 | 0.60 | 0.61 | 0.60 | 0.62 | 0.59 | 0.60 | 0.62 | 0.59 | 0.59 | 0.60 | 0.61 | 0.60 | 0.60 | 0.59 | 0.60 |
|  | lasso | 0.61 | 0.61 | 0.59 | 0.60 | 0.54 | 0.63 | 0.61 | 0.61 | 0.59 | 0.60 | 0.61 | 0.57 | 0.60 | 0.56 | 0.61 | 0.62 | 0.62 | 0.59 | 0.61 | 0.59 | 0.61 | 0.61 | 0.56 | 0.61 | 0.60 | 0.61 | 0.60 | 0.62 | 0.61 | 0.61 | 0.61 |
|  | ipflasso | 0.61 | 0.60 | 0.57 | 0.58 | 0.47 | 0.62 | 0.57 | 0.58 | 0.55 | 0.56 | 0.58 | 0.50 | 0.58 | 0.51 | 0.58 | 0.58 | 0.58 | 0.48 | 0.58 | 0.49 | 0.58 | 0.58 | 0.48 | 0.58 | 0.58 | 0.58 | 0.48 | 0.58 | 0.58 | 0.58 | 0.58 |
|  | prioritylasso | 0.61 | 0.61 | 0.59 | 0.60 | 0.54 | 0.55 | 0.52 | 0.50 | 0.54 | 0.55 | 0.51 | 0.55 | 0.60 | 0.56 | 0.60 | 0.50 | 0.50 | 0.54 | 0.50 | 0.52 | 0.50 | 0.51 | 0.53 | 0.51 | 0.60 | 0.50 | 0.53 | 0.50 | 0.50 | 0.51 | 0.51 |
| LGG | bf | 0.84 | 0.83 | 0.84 | 0.83 | 0.80 | 0.84 | 0.84 | 0.83 | 0.83 | 0.84 | 0.83 | 0.83 | 0.83 | 0.84 | 0.83 | 0.84 | 0.82 | 0.84 | 0.83 | 0.83 | 0.82 | 0.83 | 0.84 | 0.83 | 0.82 | 0.82 | 0.83 | 0.82 | 0.82 | 0.83 | 0.82 |
|  | rsf | 0.81 | 0.84 | 0.81 | 0.82 | 0.80 | 0.82 | 0.82 | 0.82 | 0.82 | 0.82 | 0.82 | 0.84 | 0.82 | 0.81 | 0.82 | 0.82 | 0.82 | 0.82 | 0.82 | 0.82 | 0.82 | 0.82 | 0.82 | 0.82 | 0.82 | 0.82 | 0.82 | 0.82 | 0.82 | 0.82 | 0.82 |
|  | lasso | 0.85 | 0.84 | 0.84 | 0.84 | 0.81 | 0.85 | 0.85 | 0.84 | 0.84 | 0.85 | 0.84 | 0.83 | 0.84 | 0.83 | 0.84 | 0.85 | 0.84 | 0.84 | 0.84 | 0.84 | 0.84 | 0.84 | 0.83 | 0.84 | 0.84 | 0.84 | 0.84 | 0.84 | 0.84 | 0.84 | 0.85 |
|  | ipflasso | 0.79 | 0.82 | 0.79 | 0.84 | 0.79 | 0.82 | 0.79 | 0.84 | 0.79 | 0.82 | 0.84 | 0.79 | 0.84 | 0.79 | 0.79 | 0.82 | 0.84 | 0.79 | 0.84 | 0.79 | 0.79 | 0.85 | 0.79 | 0.79 | 0.79 | 0.84 | 0.79 | 0.80 | 0.80 | 0.80 | 0.79 |
|  | prioritylasso | 0.85 | 0.85 | 0.84 | 0.85 | 0.80 | 0.80 | 0.79 | 0.56 | 0.69 | 0.85 | 0.76 | 0.80 | 0.85 | 0.84 | 0.70 | 0.80 | 0.59 | 0.75 | 0.56 | 0.73 | 0.77 | 0.76 | 0.80 | 0.71 | 0.66 | 0.60 | 0.73 | 0.67 | 0.73 | 0.75 | 0.64 |
| LIHC | bf | 0.63 | 0.60 | 0.68 | 0.57 | 0.61 | 0.60 | 0.67 | 0.58 | 0.53 | 0.67 | 0.56 | 0.62 | 0.61 | 0.57 | 0.56 | 0.66 | 0.59 | 0.48 | 0.65 | 0.50 | 0.56 | 0.61 | 0.39 | 0.53 | 0.58 | 0.64 | 0.51 | 0.57 | 0.62 | 0.59 | 0.62 |
|  | rsf | 0.60 | 0.59 | 0.65 | 0.53 | 0.61 | 0.59 | 0.67 | 0.58 | 0.53 | 0.65 | 0.54 | 0.61 | 0.59 | 0.56 | 0.52 | 0.66 | 0.59 | 0.52 | 0.64 | 0.50 | 0.57 | 0.60 | 0.41 | 0.53 | 0.58 | 0.64 | 0.52 | 0.57 | 0.63 | 0.59 | 0.63 |
|  | lasso | 0.64 | 0.53 | 0.67 | 0.59 | 0.62 | 0.61 | 0.70 | 0.60 | 0.57 | 0.68 | 0.60 | 0.58 | 0.59 | 0.55 | 0.61 | 0.67 | 0.60 | 0.58 | 0.60 | 0.56 | 0.59 | 0.60 | 0.54 | 0.61 | 0.59 | 0.60 | 0.59 | 0.59 | 0.60 | 0.59 | 0.60 |
|  | ipflasso | 0.53 | 0.37 | 0.67 | 0.50 | 0.43 | 0.52 | 0.67 | 0.46 | 0.53 | 0.68 | 0.49 | 0.54 | 0.50 | 0.54 | 0.49 | 0.68 | 0.46 | 0.50 | 0.46 | 0.50 | 0.47 | 0.50 | 0.50 | 0.49 | 0.49 | 0.46 | 0.40 | 0.47 | 0.47 | 0.49 | 0.47 |
|  | prioritylasso | 0.61 | 0.56 | 0.66 | 0.56 | 0.58 | 0.58 | 0.51 | 0.55 | 0.53 | 0.54 | 0.56 | 0.59 | 0.62 | 0.56 | 0.61 | 0.50 | 0.55 | 0.59 | 0.55 | 0.41 | 0.54 | 0.45 | 0.54 | 0.46 | 0.61 | 0.51 | 0.57 | 0.54 | 0.55 | 0.48 | 0.48 |
| LUAD | bf | 0.70 | 0.71 | 0.68 | 0.70 | 0.68 | 0.69 | 0.67 | 0.66 | 0.66 | 0.68 | 0.71 | 0.68 | 0.68 | 0.65 | 0.68 | 0.68 | 0.67 | 0.67 | 0.66 | 0.65 | 0.64 | 0.69 | 0.66 | 0.68 | 0.68 | 0.67 | 0.63 | 0.66 | 0.64 | 0.67 | 0.65 |
|  | rsf | 0.68 | 0.71 | 0.66 | 0.69 | 0.64 | 0.69 | 0.66 | 0.68 | 0.65 | 0.67 | 0.70 | 0.66 | 0.66 | 0.64 | 0.65 | 0.66 | 0.68 | 0.65 | 0.66 | 0.63 | 0.65 | 0.67 | 0.64 | 0.65 | 0.63 | 0.66 | 0.64 | 0.65 | 0.64 | 0.63 | 0.64 |
|  | lasso | 0.70 | 0.70 | 0.70 | 0.70 | 0.70 | 0.70 | 0.70 | 0.70 | 0.70 | 0.70 | 0.70 | 0.70 | 0.69 | 0.70 | 0.70 | 0.70 | 0.70 | 0.70 | 0.70 | 0.70 | 0.70 | 0.70 | 0.70 | 0.70 | 0.70 | 0.70 | 0.70 | 0.70 | 0.70 | 0.70 | 0.70 |
|  | ipflasso | 0.71 | 0.71 | 0.71 | 0.71 | 0.72 | 0.71 | 0.71 | 0.71 | 0.71 | 0.71 | 0.71 | 0.71 | 0.71 | 0.71 | 0.71 | 0.71 | 0.71 | 0.71 | 0.71 | 0.72 | 0.71 | 0.71 | 0.71 | 0.71 | 0.71 | 0.71 | 0.71 | 0.71 | 0.71 | 0.71 | 0.71 |
|  | prioritylasso | 0.70 | 0.70 | 0.69 | 0.69 | 0.69 | 0.69 | 0.68 | 0.64 | 0.60 | 0.70 | 0.65 | 0.65 | 0.69 | 0.68 | 0.68 | 0.67 | 0.62 | 0.61 | 0.62 | 0.60 | 0.61 | 0.64 | 0.65 | 0.63 | 0.67 | 0.63 | 0.60 | 0.61 | 0.62 | 0.63 | 0.59 |
| LUSC | bf | 0.52 | 0.54 | 0.48 | 0.49 | 0.52 | 0.52 | 0.49 | 0.50 | 0.52 | 0.47 | 0.50 | 0.54 | 0.46 | 0.48 | 0.50 | 0.48 | 0.50 | 0.51 | 0.48 | 0.50 | 0.48 | 0.47 | 0.47 | 0.49 | 0.47 | 0.48 | 0.48 | 0.49 | 0.48 | 0.47 | 0.45 |
|  | rsf | 0.51 | 0.54 | 0.47 | 0.50 | 0.49 | 0.51 | 0.48 | 0.51 | 0.50 | 0.46 | 0.49 | 0.50 | 0.44 | 0.46 | 0.51 | 0.48 | 0.50 | 0.50 | 0.47 | 0.48 | 0.48 | 0.46 | 0.46 | 0.49 | 0.45 | 0.46 | 0.48 | 0.45 | 0.45 | 0.47 | 0.46 |
|  | lasso | 0.57 | 0.56 | 0.56 | 0.53 | 0.56 | 0.57 | 0.56 | 0.54 | 0.56 | 0.55 | 0.53 | 0.56 | 0.53 | 0.56 | 0.53 | 0.57 | 0.54 | 0.56 | 0.54 | 0.56 | 0.54 | 0.53 | 0.56 | 0.54 | 0.54 | 0.54 | 0.56 | 0.54 | 0.54 | 0.54 | 0.54 |
|  | ipflasso | 0.55 | 0.55 | 0.55 | 0.50 | 0.52 | 0.54 | 0.54 | 0.49 | 0.49 | 0.54 | 0.50 | 0.54 | 0.50 | 0.53 | 0.50 | 0.55 | 0.49 | 0.48 | 0.49 | 0.48 | 0.49 | 0.50 | 0.52 | 0.50 | 0.49 | 0.49 | 0.48 | 0.49 | 0.48 | 0.50 | 0.48 |
|  | prioritylasso | 0.57 | 0.56 | 0.56 | 0.51 | 0.56 | 0.57 | 0.56 | 0.50 | 0.56 | 0.54 | 0.49 | 0.55 | 0.51 | 0.55 | 0.54 | 0.56 | 0.48 | 0.56 | 0.48 | 0.56 | 0.48 | 0.48 | 0.55 | 0.48 | 0.52 | 0.48 | 0.55 | 0.50 | 0.49 | 0.49 | 0.52 |
| PAAD | bf | 0.69 | 0.65 | 0.59 | 0.65 | 0.66 | 0.68 | 0.67 | 0.67 | 0.69 | 0.59 | 0.65 | 0.63 | 0.62 | 0.59 | 0.65 | 0.65 | 0.66 | 0.68 | 0.67 | 0.67 | 0.68 | 0.62 | 0.59 | 0.66 | 0.62 | 0.66 | 0.66 | 0.66 | 0.66 | 0.62 | 0.66 |
|  | rsf | 0.68 | 0.62 | 0.56 | 0.64 | 0.65 | 0.67 | 0.62 | 0.66 | 0.67 | 0.56 | 0.64 | 0.64 | 0.60 | 0.58 | 0.64 | 0.63 | 0.66 | 0.67 | 0.63 | 0.63 | 0.66 | 0.60 | 0.58 | 0.64 | 0.61 | 0.63 | 0.63 | 0.66 | 0.62 | 0.60 | 0.63 |
|  | lasso | 0.64 | 0.64 | 0.64 | 0.64 | 0.64 | 0.64 | 0.64 | 0.64 | 0.64 | 0.64 | 0.64 | 0.64 | 0.64 | 0.64 | 0.64 | 0.64 | 0.64 | 0.64 | 0.64 | 0.64 | 0.64 | 0.64 | 0.64 | 0.64 | 0.64 | 0.64 | 0.64 | 0.64 | 0.64 | 0.64 | 0.64 |
|  | ipflasso | 0.63 | 0.63 | 0.63 | 0.60 | 0.63 | 0.63 | 0.63 | 0.60 | 0.63 | 0.63 | 0.61 | 0.63 | 0.59 | 0.63 | 0.59 | 0.63 | 0.60 | 0.63 | 0.60 | 0.63 | 0.60 | 0.60 | 0.63 | 0.60 | 0.60 | 0.61 | 0.63 | 0.61 | 0.61 | 0.62 | 0.59 |
|  | prioritylasso | 0.66 | 0.63 | 0.64 | 0.63 | 0.64 | 0.59 | 0.62 | 0.61 | 0.62 | 0.62 | 0.62 | 0.64 | 0.64 | 0.64 | 0.64 | 0.63 | 0.55 | 0.62 | 0.56 | 0.61 | 0.56 | 0.60 | 0.63 | 0.60 | 0.63 | 0.58 | 0.59 | 0.59 | 0.59 | 0.60 | 0.56 |
| SARC | bf | 0.71 | 0.68 | 0.63 | 0.74 | 0.66 | 0.72 | 0.67 | 0.74 | 0.70 | 0.64 | 0.75 | 0.69 | 0.72 | 0.62 | 0.73 | 0.67 | 0.74 | 0.72 | 0.73 | 0.68 | 0.74 | 0.73 | 0.63 | 0.75 | 0.72 | 0.73 | 0.69 | 0.75 | 0.73 | 0.73 | 0.72 |
|  | rsf | 0.70 | 0.69 | 0.60 | 0.73 | 0.67 | 0.70 | 0.66 | 0.72 | 0.70 | 0.62 | 0.73 | 0.69 | 0.69 | 0.62 | 0.74 | 0.65 | 0.73 | 0.69 | 0.70 | 0.65 | 0.73 | 0.69 | 0.64 | 0.75 | 0.68 | 0.69 | 0.65 | 0.73 | 0.69 | 0.70 | 0.71 |
|  | lasso | 0.65 | 0.67 | 0.63 | 0.71 | 0.65 | 0.65 | 0.65 | 0.69 | 0.65 | 0.65 | 0.69 | 0.66 | 0.70 | 0.64 | 0.69 | 0.65 | 0.69 | 0.65 | 0.69 | 0.65 | 0.69 | 0.69 | 0.65 | 0.69 | 0.69 | 0.69 | 0.65 | 0.69 | 0.69 | 0.69 | 0.69 |
|  | ipflasso | 0.65 | 0.65 | 0.65 | 0.74 | 0.65 | 0.65 | 0.65 | 0.75 | 0.64 | 0.65 | 0.74 | 0.65 | 0.73 | 0.65 | 0.73 | 0.65 | 0.74 | 0.65 | 0.74 | 0.65 | 0.74 | 0.73 | 0.65 | 0.73 | 0.74 | 0.74 | 0.65 | 0.74 | 0.74 | 0.73 | 0.74 |
|  | prioritylasso | 0.67 | 0.68 | 0.63 | 0.73 | 0.65 | 0.63 | 0.59 | 0.47 | 0.60 | 0.66 | 0.62 | 0.66 | 0.74 | 0.62 | 0.73 | 0.60 | 0.47 | 0.62 | 0.47 | 0.60 | 0.47 | 0.63 | 0.64 | 0.62 | 0.72 | 0.47 | 0.64 | 0.47 | 0.48 | 0.62 | 0.49 |
| SKCM | bf | 0.68 | 0.63 | 0.63 | 0.62 | 0.64 | 0.67 | 0.66 | 0.63 | 0.65 | 0.63 | 0.61 | 0.62 | 0.62 | 0.61 | 0.60 | 0.66 | 0.64 | 0.65 | 0.64 | 0.65 | 0.60 | 0.62 | 0.62 | 0.59 | 0.59 | 0.64 | 0.65 | 0.62 | 0.61 | 0.59 | 0.61 |
|  | rsf | 0.66 | 0.64 | 0.62 | 0.61 | 0.64 | 0.65 | 0.63 | 0.63 | 0.64 | 0.62 | 0.61 | 0.61 | 0.62 | 0.60 | 0.59 | 0.64 | 0.63 | 0.63 | 0.62 | 0.62 | 0.61 | 0.62 | 0.60 | 0.59 | 0.60 | 0.63 | 0.62 | 0.61 | 0.61 | 0.60 | 0.61 |
|  | lasso | 0.66 | 0.66 | 0.67 | 0.66 | 0.66 | 0.66 | 0.66 | 0.66 | 0.66 | 0.67 | 0.67 | 0.66 | 0.67 | 0.66 | 0.66 | 0.66 | 0.66 | 0.66 | 0.67 | 0.67 | 0.67 | 0.66 | 0.66 | 0.66 | 0.66 | 0.67 | 0.66 | 0.67 | 0.66 | 0.66 | 0.66 |
|  | ipflasso | 0.64 | 0.64 | 0.64 | 0.60 | 0.64 | 0.64 | 0.64 | 0.60 | 0.64 | 0.64 | 0.58 | 0.64 | 0.58 | 0.64 | 0.58 | 0.64 | 0.60 | 0.64 | 0.61 | 0.64 | 0.61 | 0.60 | 0.64 | 0.60 | 0.59 | 0.61 | 0.64 | 0.61 | 0.61 | 0.59 | 0.60 |
|  | prioritylasso | 0.67 | 0.66 | 0.65 | 0.67 | 0.66 | 0.66 | 0.64 | 0.57 | 0.63 | 0.65 | 0.54 | 0.63 | 0.66 | 0.66 | 0.66 | 0.64 | 0.55 | 0.64 | 0.56 | 0.64 | 0.56 | 0.58 | 0.65 | 0.59 | 0.66 | 0.57 | 0.61 | 0.58 | 0.58 | 0.57 | 0.54 |
| STAD | bf | 0.64 | 0.63 | 0.56 | 0.59 | 0.59 | 0.61 | 0.58 | 0.58 | 0.62 | 0.56 | 0.59 | 0.64 | 0.54 | 0.55 | 0.59 | 0.57 | 0.57 | 0.61 | 0.54 | 0.58 | 0.57 | 0.53 | 0.55 | 0.58 | 0.53 | 0.55 | 0.57 | 0.55 | 0.54 | 0.53 | 0.54 |
|  | rsf | 0.61 | 0.65 | 0.53 | 0.56 | 0.59 | 0.59 | 0.57 | 0.57 | 0.61 | 0.55 | 0.57 | 0.63 | 0.54 | 0.54 | 0.57 | 0.56 | 0.56 | 0.60 | 0.55 | 0.57 | 0.58 | 0.53 | 0.55 | 0.58 | 0.54 | 0.55 | 0.57 | 0.58 | 0.56 | 0.54 | 0.56 |
|  | lasso | 0.62 | 0.63 | 0.62 | 0.62 | 0.61 | 0.63 | 0.63 | 0.63 | 0.63 | 0.63 | 0.62 | 0.62 | 0.63 | 0.62 | 0.62 | 0.63 | 0.63 | 0.63 | 0.63 | 0.63 | 0.63 | 0.63 | 0.62 | 0.62 | 0.62 | 0.63 | 0.63 | 0.63 | 0.62 | 0.62 | 0.62 |
|  | ipflasso | 0.61 | 0.61 | 0.61 | 0.59 | 0.61 | 0.61 | 0.61 | 0.59 | 0.62 | 0.61 | 0.60 | 0.62 | 0.60 | 0.62 | 0.60 | 0.61 | 0.60 | 0.61 | 0.60 | 0.61 | 0.60 | 0.60 | 0.61 | 0.59 | 0.61 | 0.60 | 0.61 | 0.60 | 0.59 | 0.59 | 0.59 |
|  | prioritylasso | 0.62 | 0.63 | 0.62 | 0.62 | 0.61 | 0.62 | 0.61 | 0.59 | 0.59 | 0.63 | 0.61 | 0.62 | 0.61 | 0.62 | 0.62 | 0.61 | 0.53 | 0.61 | 0.53 | 0.60 | 0.52 | 0.59 | 0.62 | 0.59 | 0.61 | 0.57 | 0.60 | 0.57 | 0.54 | 0.58 | 0.54 |
| UCEC | bf | 0.73 | 0.74 | 0.73 | 0.72 | 0.71 | 0.72 | 0.72 | 0.72 | 0.71 | 0.74 | 0.72 | 0.72 | 0.70 | 0.73 | 0.71 | 0.72 | 0.70 | 0.69 | 0.70 | 0.70 | 0.69 | 0.70 | 0.73 | 0.70 | 0.70 | 0.69 | 0.71 | 0.68 | 0.69 | 0.70 | 0.69 |
|  | rsf | 0.72 | 0.74 | 0.72 | 0.70 | 0.70 | 0.73 | 0.71 | 0.72 | 0.69 | 0.72 | 0.71 | 0.71 | 0.70 | 0.66 | 0.70 | 0.72 | 0.73 | 0.70 | 0.71 | 0.68 | 0.70 | 0.70 | 0.67 | 0.69 | 0.68 | 0.71 | 0.69 | 0.71 | 0.70 | 0.69 | 0.70 |
|  | lasso | 0.72 | 0.74 | 0.74 | 0.72 | 0.73 | 0.72 | 0.73 | 0.73 | 0.73 | 0.74 | 0.72 | 0.74 | 0.74 | 0.74 | 0.74 | 0.73 | 0.73 | 0.73 | 0.72 | 0.73 | 0.73 | 0.73 | 0.74 | 0.73 | 0.73 | 0.73 | 0.73 | 0.73 | 0.73 | 0.74 | 0.73 |
|  | ipflasso | 0.72 | 0.72 | 0.72 | 0.70 | 0.72 | 0.72 | 0.72 | 0.72 | 0.72 | 0.72 | 0.71 | 0.72 | 0.72 | 0.72 | 0.72 | 0.72 | 0.70 | 0.72 | 0.72 | 0.72 | 0.72 | 0.70 | 0.72 | 0.70 | 0.70 | 0.71 | 0.72 | 0.71 | 0.72 | 0.72 | 0.72 |
|  | prioritylasso | 0.73 | 0.74 | 0.73 | 0.72 | 0.73 | 0.72 | 0.72 | 0.67 | 0.69 | 0.73 | 0.72 | 0.72 | 0.73 | 0.73 | 0.74 | 0.71 | 0.67 | 0.70 | 0.67 | 0.69 | 0.68 | 0.71 | 0.73 | 0.71 | 0.73 | 0.65 | 0.68 | 0.66 | 0.67 | 0.71 | 0.66 |

**Dataset specific ranks of each block combination**


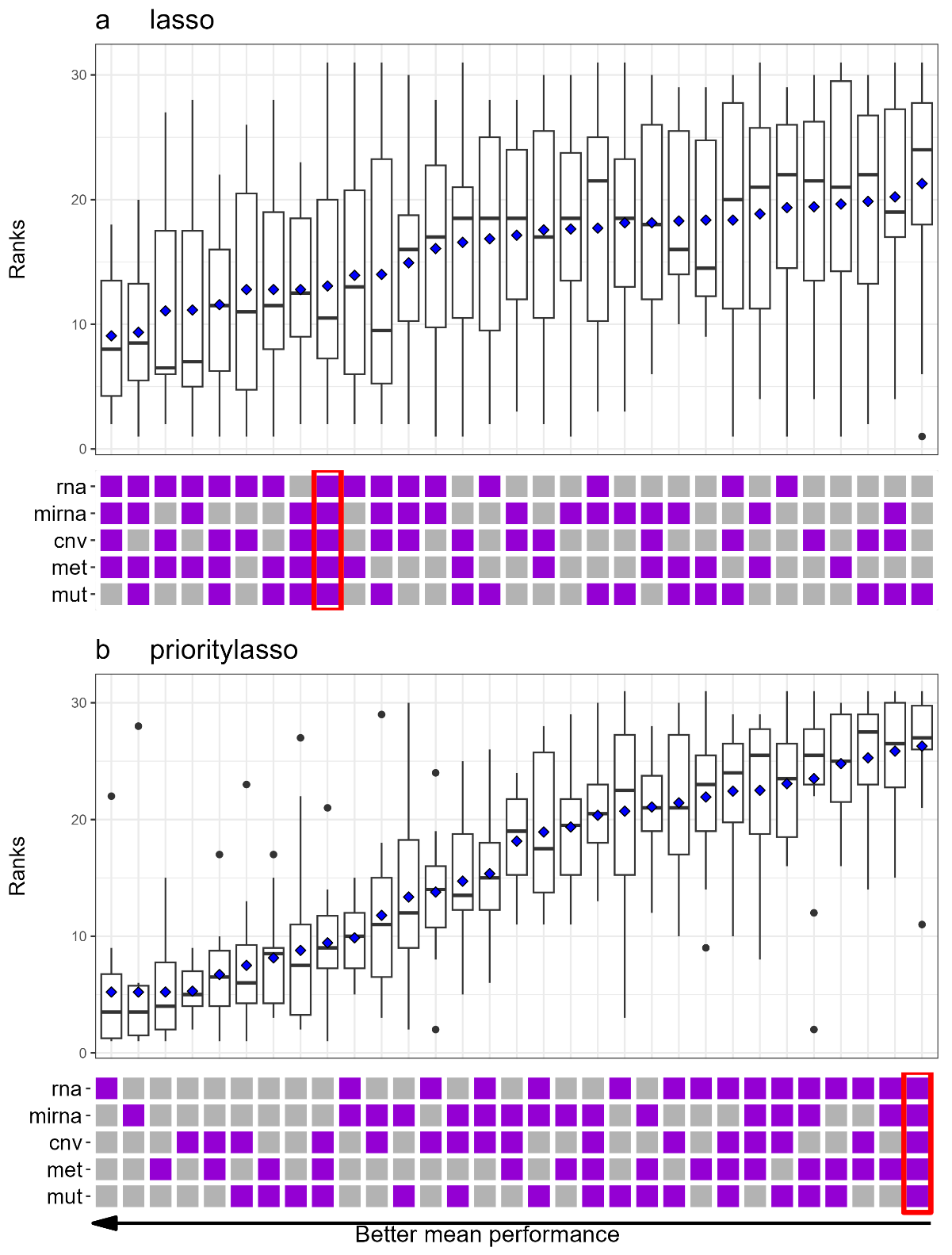


Figure S5: Dataset specific ranks of each block combination (ibrier). The ranks of each combination among all 31 combinations are shown. The purple squares indicate which omics block(s) were included in the respective combinations. The values shown by the boxplots are the ranks achieved across all 14 datasets, where the blue diamonds represent the means of the ranks. The upper (a) and lower (b) panels show the results obtained for lasso and prioritylasso, respectively. Smaller ranks indicate a better predictive performance. The combinations are sorted in increasing order according to the mean ranks across the datasets, which is why the combinations further to the left tend to perform better. cnv: CNV, mirna: miRNA, mut: DNAseq, met: methylation, rna: mRNA.


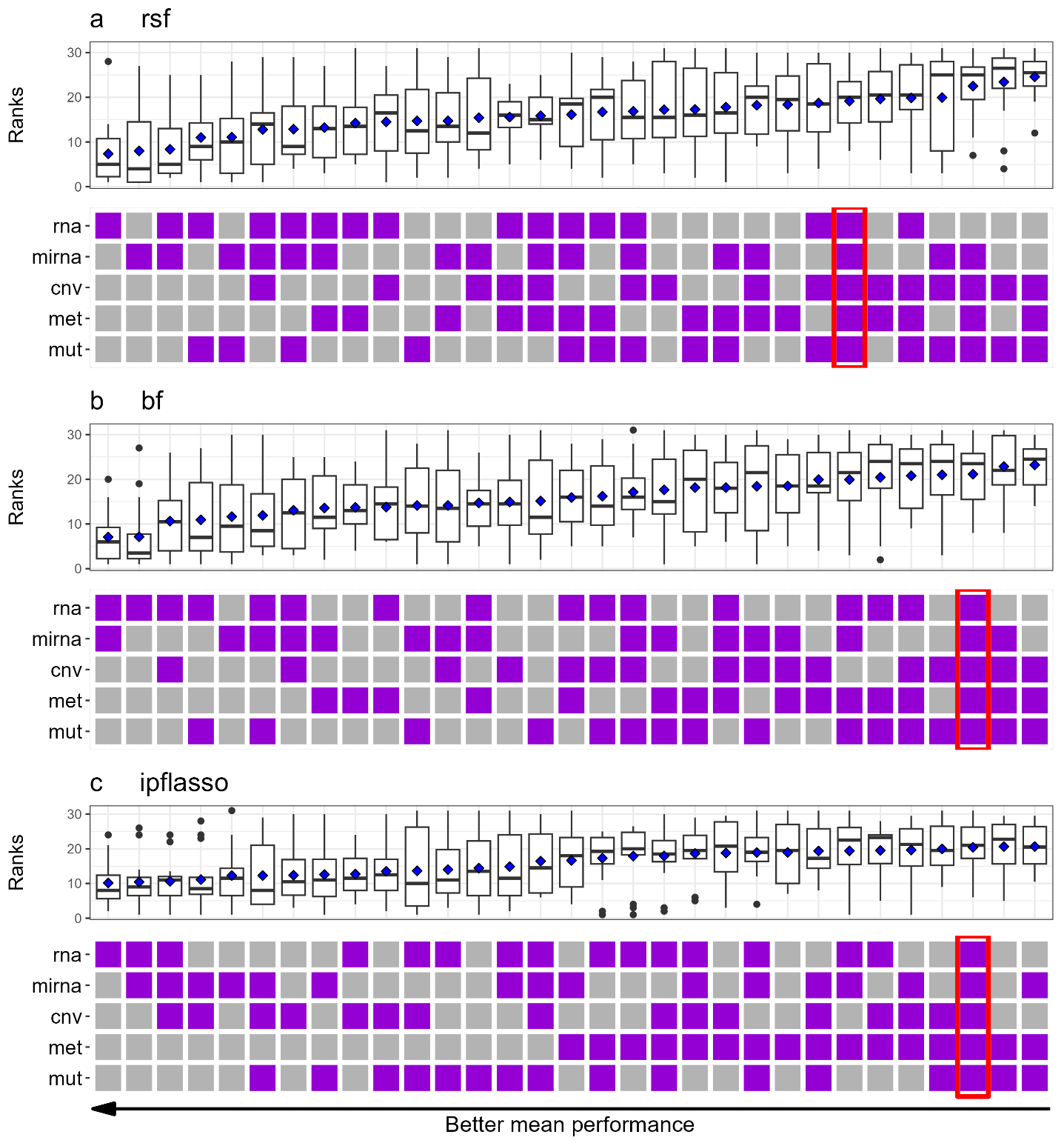


Figure S6: Dataset specific ranks of each block combination (cindex). The ranks of each combination among all 31 combinations are shown. The purple squares indicate which omics block(s) were included in the respective combinations. The values shown by the boxplots are the ranks achieved across all 14 datasets, where the blue diamonds represent the means of the ranks. The upper (a), middle (b), and lower (c) panels show the results obtained for rsf, bf, and ipflasso, respectively. Smaller ranks indicate a better predictive performance. The combinations are sorted in increasing order according to the mean ranks across the datasets, which is why the combinations further to the left tend to perform better. cnv: CNV, mirna: miRNA, mut: DNAseq, met: methylation, rna: mRNA.


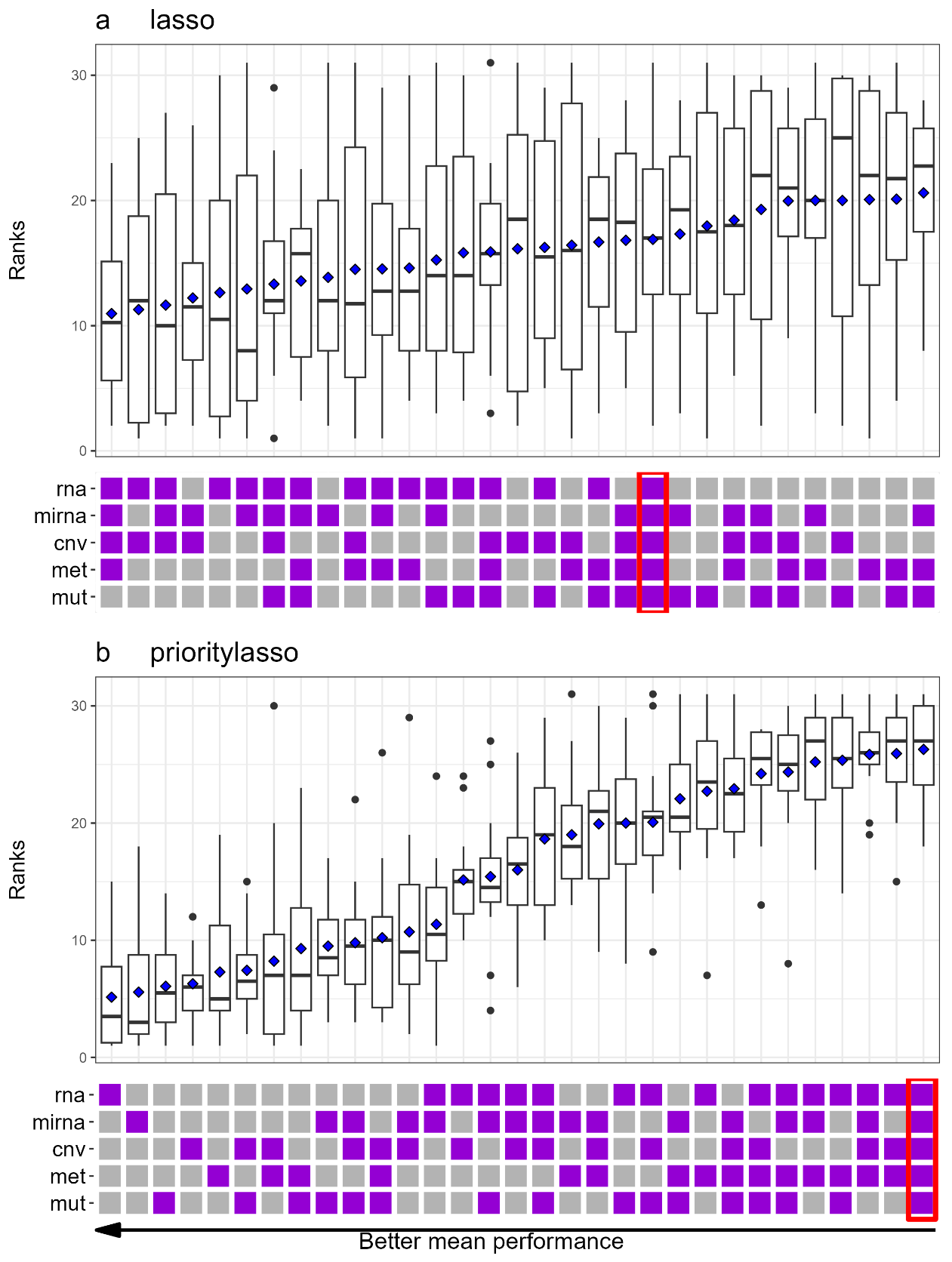


Figure S7: Dataset specific ranks of each block combination (cindex). The ranks of each combination among all 31 combinations are shown. The purple squares indicate which omics block(s) were included in the respective combinations. The values shown by the boxplots are the ranks achieved across all 14 datasets, where the blue diamonds represent the means of the ranks. The upper (a) and lower (b) panels show the results obtained for lasso and prioritylasso, respectively. Smaller ranks indicate a better predictive performance. The combinations are sorted in increasing order according to the mean ranks across the datasets, which is why the combinations further to the left tend to perform better. cnv: CNV, mirna: miRNA, mut: DNAseq, met: methylation, rna: mRNA.

**Relationship between the dataset specific ranks and the number of events**

As also described in the “Ranking of the predictive information contained in all block combinations per prediction method” section of the main paper, there is a possibility that the number of observations in the available datasets is not sufficient to adequately exploit the predictive information contained in combinations with many omics blocks. If this were the case, contrary to the results described above, combinations with many blocks might outperform combinations with fewer blocks for large datasets. If so, a trend should be observable where combinations with many blocks rank better for larger datasets than for smaller datasets. Conversely, a trend should be observed where combinations with fewer blocks rank worse for larger datasets than for smaller ones.

We investigated this using the available datasets, limiting ourselves for clarity to six combinations: the five consisting of only one of the five omics blocks and the one containing all five omics blocks. In Figures S8 to S11, for each of these combinations, separated by prediction method and performance measure, the dataset-specific ranks are plotted against the number of events. There is no observable trend that the combination with all blocks ranks better for larger numbers of events. An exception is Lasso with respect to ibrier. As described in the main paper, this method was the only one for which using more blocks led to better prediction results regarding the ibrier. Moreover, for the cindex, the ranks of the combinations with only mRNA or only miRNA improved for larger numbers of events. Beyond these observations, no clear relationships between the dataset-specific ranks and the number of events are visible. In univariable linear models, no significant effects of the number of events on the ranks were found after Bonferroni-Holm adjustment (results not shown). These findings do not allow the conclusion that combinations with many blocks would benefit from larger datasets in prediction.

**
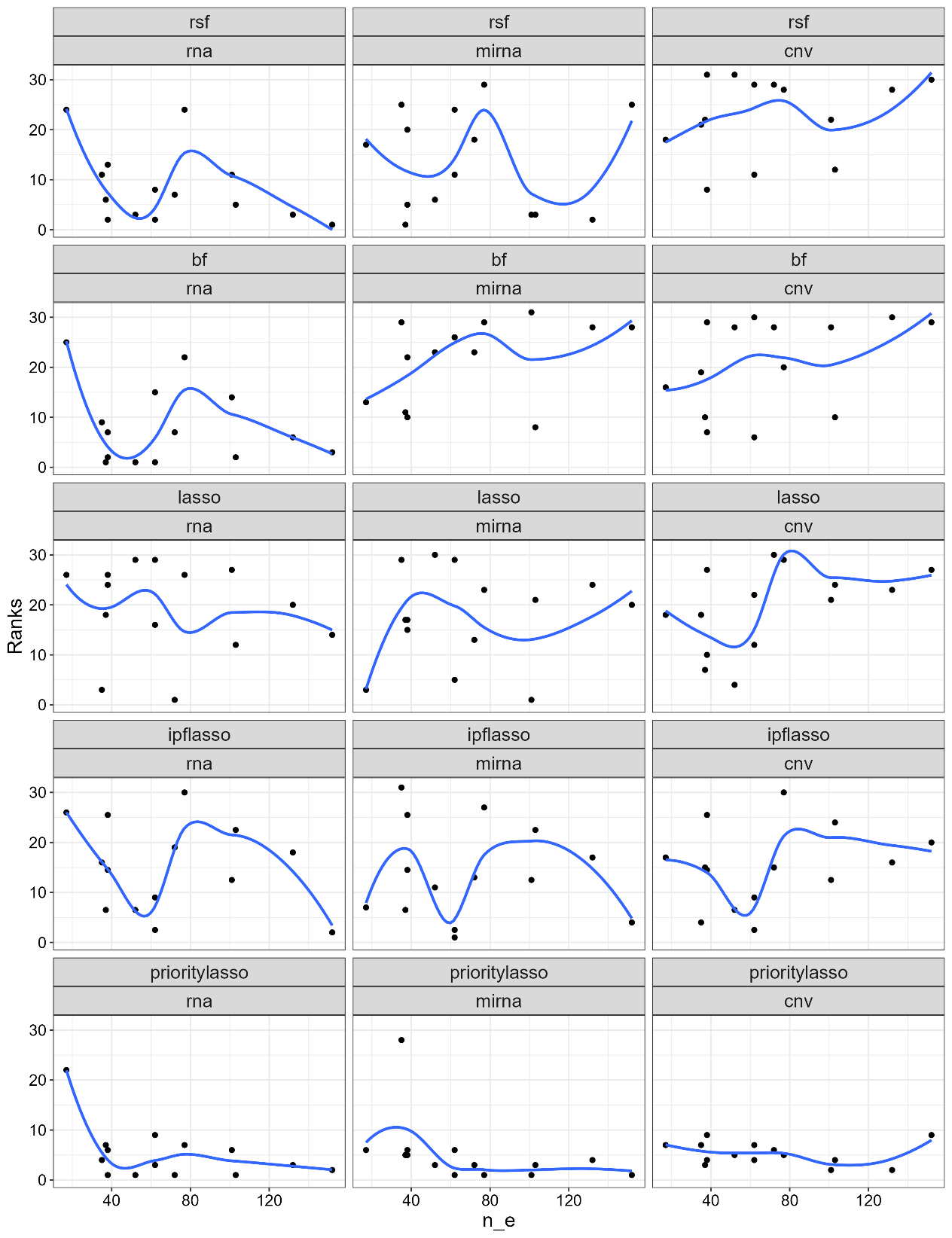
**

Figure S8: Relationship between the dataset specific ranks (ibrier) and the number of events (n_e) for mRNA, miRNA and CNV. Each point represents one of the 14 datasets. The blue curves were determined by local regression (LOESS) with Gaussian error terms for simplicity, using a neighborhood parameter of 0.75 and second-degree polynomials. cnv: CNV, mirna: miRNA, mut: DNAseq, met: methylation, rna: mRNA.


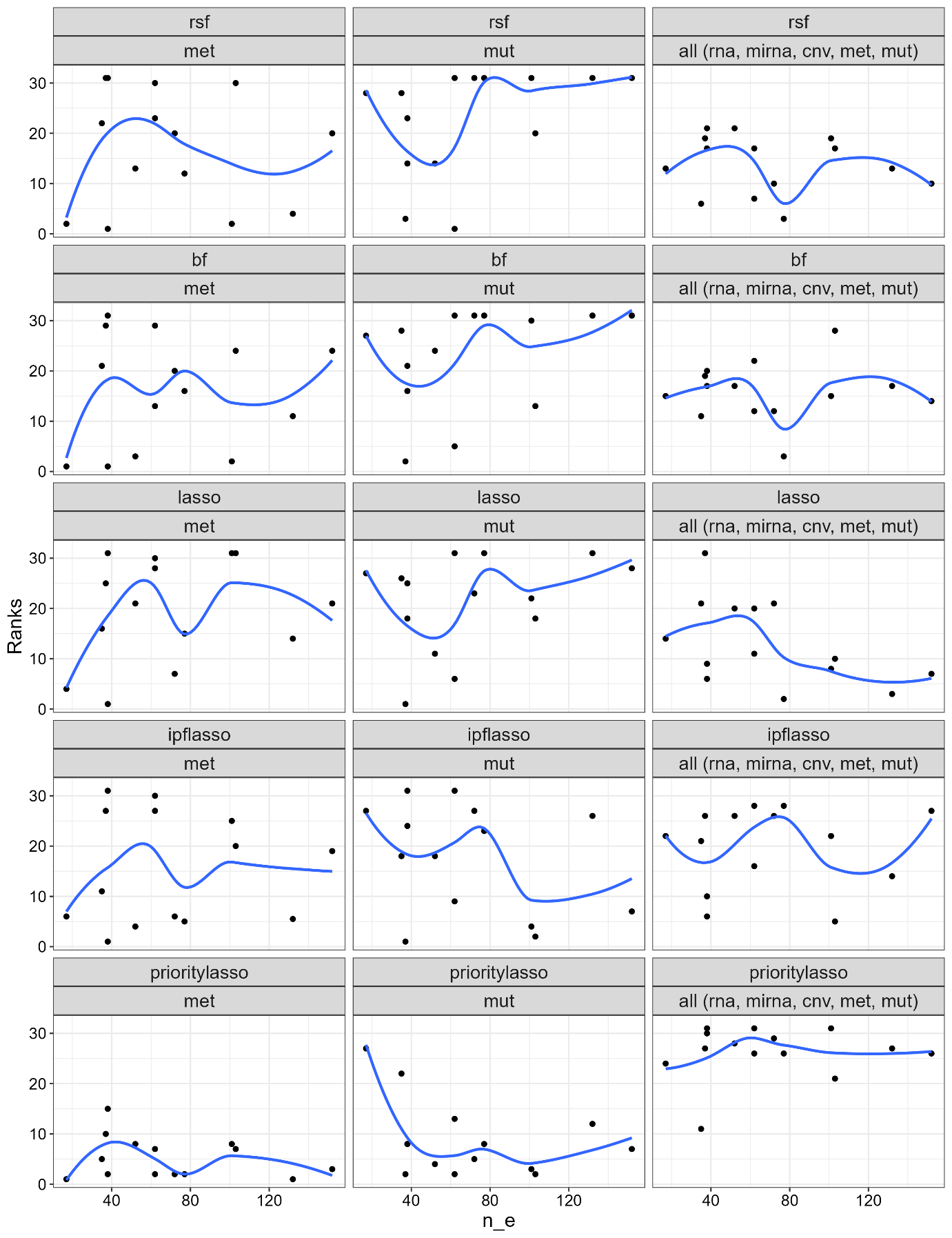


Figure S9: Relationship between the dataset specific ranks (ibrier) and the number of events (n_e) for methylation, DNAseq and the combination of all omics blocks. Each point represents one of the 14 datasets. The blue curves were determined by local regression (LOESS) with Gaussian error terms for simplicity, using a neighborhood parameter of 0.75 and second-degree polynomials. cnv: CNV, mirna: miRNA, mut: DNAseq, met: methylation, rna: mRNA.


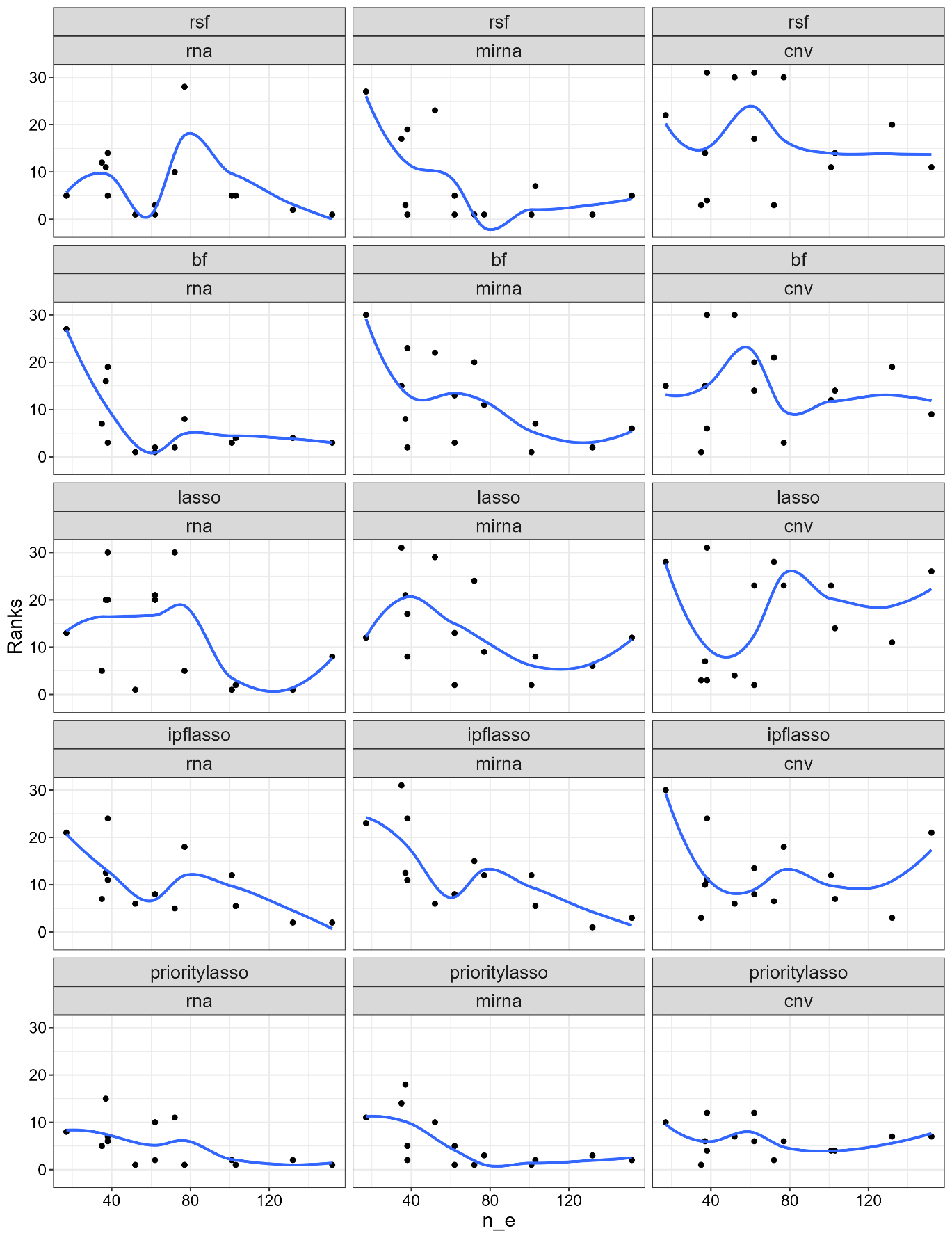


Figure S10: Relationship between the dataset specific ranks (cindex) and the number of events (n_e) for mRNA, miRNA and CNV. Each point represents one of the 14 datasets. The blue curves were determined by local regression (LOESS) with Gaussian error terms for simplicity, using a neighborhood parameter of 0.75 and second-degree polynomials. cnv: CNV, mirna: miRNA, mut: DNAseq, met: methylation, rna: mRNA.

**
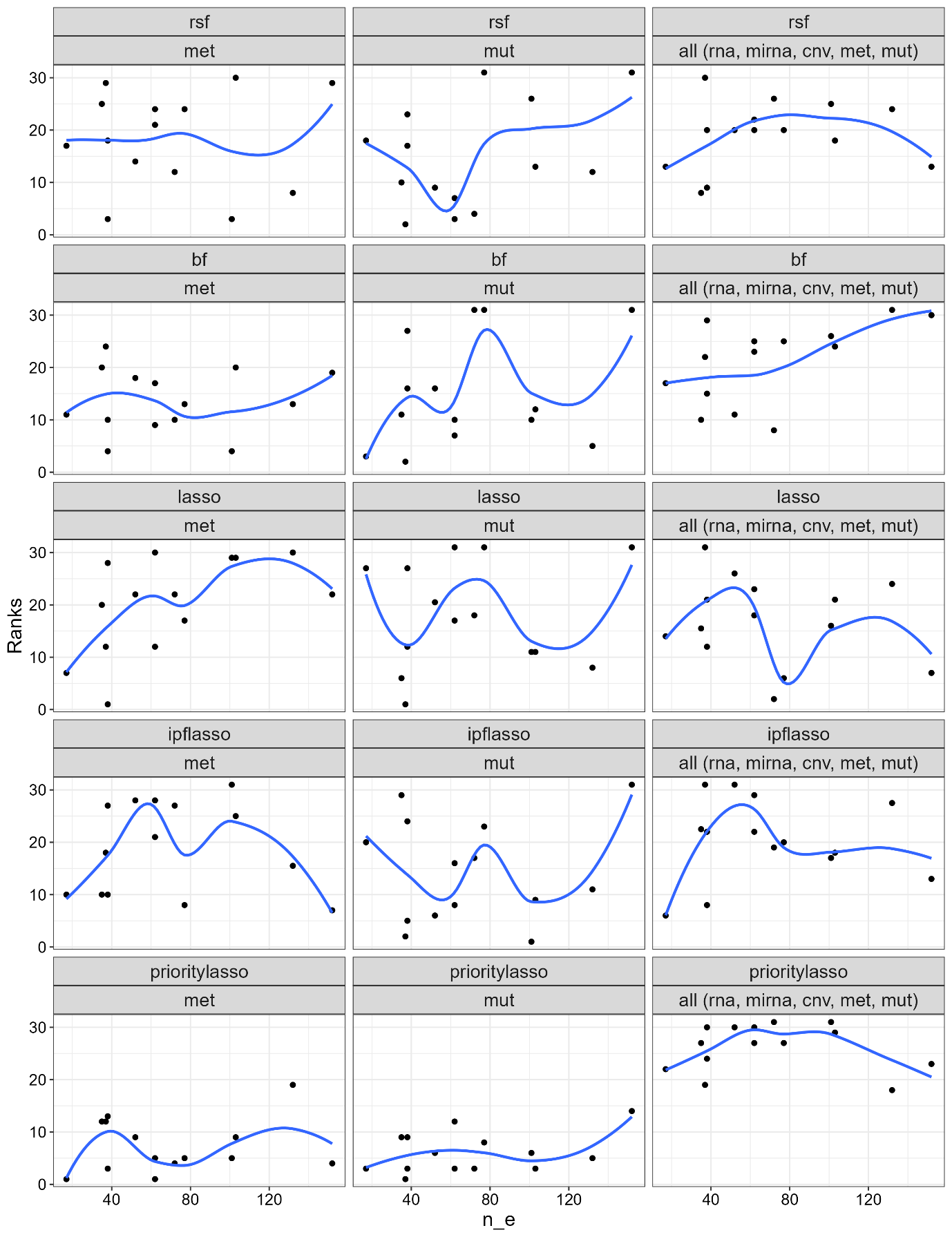
**

Figure S11: Relationship between the dataset specific ranks (cindex) and the number of events (n_e) for methylation, DNAseq and the combination of all omics blocks. Each point represents one of the 14 datasets. The blue curves were determined by local regression (LOESS) with Gaussian error terms for simplicity, using a neighborhood parameter of 0.75 and second-degree polynomials. cnv: CNV, mirna: miRNA, mut: DNAseq, met: methylation, rna: mRNA.

**Results of the bootstrap analysis**

As described in the “Ranking of the predictive information contained in all block combinations per prediction method” section of the main paper, to assess statistical uncertainty and to derive 95% confidence intervals, we performed bootstrap analysis [8], [9] at the level of the 14 included datasets. For each sub-analysis (e.g., those shown in Figure 1 in the main paper and Figure S5), we drew 5,000 bootstrap samples, and each time recalculated the mean ranks associated with the different combinations and the ranks of each mean rank among all other mean ranks. These ranks of the mean ranks are hereafter referred to as ‘positions’. Subsequently, we computed the mean positions across the 5,000 bootstrap samples and 95% percentile confidence intervals for these mean positions. These intervals are calculated by taking the 2.5% and 97.5% quantiles of the 5,000 positions calculated using the bootstrap samples. It is important to note that the interpretation of confidence intervals is closely related to that of statistical tests. For example, if a confidence interval does not contain a value c, a (bootstrap-based) statistical test would conclude that the corresponding mean position is significantly different from c. Conversely, if the confidence interval contains c, a statistical test would not conclude that the mean position is significantly different from c. In this manner, the following results can also be interpreted in terms of statistical significance.

The results corresponding to Figure 1 in the main paper are shown in Table S4. As expected from the high variability observed across the datasets, the confidence intervals are wide in all cases. However, for rsf and bf, our general conclusions made above can be confirmed. It can be seen that the upper bounds of the confidence intervals obtained for the best-performing combinations, which tended to include only a few blocks, are still relatively low in most cases. This confirms that these are indeed among the better combinations. For example, the confidence intervals obtained for using only mRNA suggest that these are among the ten best combinations. For ipflasso, however, the confidence intervals are very wide, which is why for these we cannot confirm that the best combinations are significantly better. For all three methods (rsf, bf, and ipflasso), the lower bounds of the confidence intervals obtained for the combination of all five blocks are greater than five, which confirms that using all blocks generally does not lead to the best predictive performance. The results of the bootstrap analysis for lasso and prioritylasso are shown in Table S5. For lasso, the confidence intervals tend to be very wide, which is why we cannot draw reliable conclusions about the rankings of the block combinations from this analysis. This result was expected given the large variability of the results across datasets for lasso (see Figure S5). For prioritylasso, the confidence intervals tended to be much narrower. However, as noted above, in the following subsection it will be seen that prioritylasso tended to produce worse prediction results compared to other prediction methods, which is why we do not interpret these results any further.

The results of the bootstrap analysis obtained for the cindex (Tables S7 and S8 in Additional file 1) are quite similar to those obtained for the ibrier. The confidence intervals tend to be slightly narrower. For all prediction methods with the exception of ipflasso, the values of the mean positions and the lower limits of their confidence intervals are much higher for the cindex when all blocks are combined. This may indicate that the cindex suffers more from the inclusion of unnecessary blocks. Note that we did not adjust the confidence intervals for multiple testing. This choice was made because, in the interpretation of the results, we did not consider each individual block combination. Instead, we focused on verifying a few specific observations, namely whether the combinations with the best mean positions are indeed among the best positions, and whether the combinations of all blocks are significantly worse than the combinations with the best positions.

Table S4: Results of the bootstrap analysis (ibrier). The purple squares indicate which omics block(s) were included in the respective combinations. For the corresponding results obtained for lasso and prioritylasso, see Table S5. The rows are ordered according to the positions obtained for rsf calculated using all datasets (without bootstrap). Low values correspond to a better and high values to a worse ranking. The columns “mean” and “ci” show the mean positions calculated using the 5000 bootstrap samples and their 95% percentile confidence intervals. The combination using all five blocks is marked with a red box. Cnv: CNV, mirna: miRNA, mut: DNAseq, met: methylation, rna: mRNA.

| No. | Combination | | | | | rsf | | bf | | ipflasso | |
| --- | --- | --- | --- | --- | --- | --- | --- | --- | --- | --- | --- |
|  | mut | met | cnv | mirna | rna | mean | ci | mean | ci | mean | ci |
| 1 | 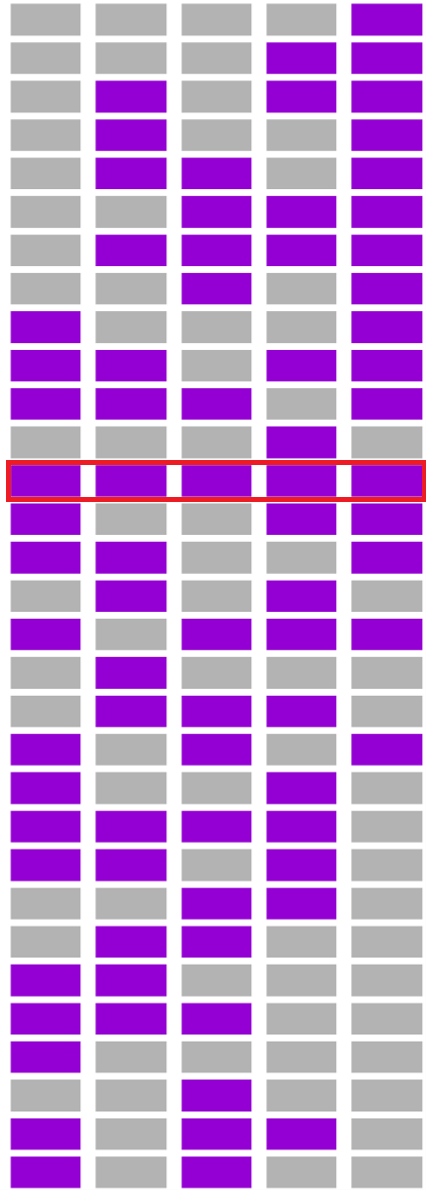 | | | | | 2.8 | [1.0, 10.0] | 2.0 | [1.0, 8.0] | 13.4 | [2.0, 28.0] |
| 2 |  |  |  |  |  | 3.2 | [1.0, 11.0] | 5.1 | [1.0, 16.0] | 6.8 | [1.0, 20.0] |
| 3 |  |  |  |  |  | 3.4 | [1.0, 9.0] | 5.7 | [1.0, 13.0] | 14.1 | [1.0, 27.0] |
| 4 |  |  |  |  |  | 5.3 | [1.0, 14.0] | 5.4 | [1.0, 11.0] | 21.9 | [4.0, 31.0] |
| 5 |  |  |  |  |  | 7.1 | [2.0, 14.0] | 4.8 | [1.0, 10.0] | 20.2 | [5.0, 30.0] |
| 6 |  |  |  |  |  | 8.4 | [3.0, 18.0] | 9.7 | [4.0, 20.0] | 11.6 | [1.0, 26.0] |
| 7 |  |  |  |  |  | 9.1 | [4.0, 16.0] | 7.0 | [2.0, 14.0] | 11.7 | [1.0, 25.0] |
| 8 |  |  |  |  |  | 9.9 | [2.0, 20.0] | 4.0 | [1.0, 12.0] | 12.9 | [1.0, 29.0] |
| 9 |  |  |  |  |  | 9.5 | [1.0, 19.0] | 9.8 | [2.0, 21.0] | 7.3 | [1.0, 22.0] |
| 10 |  |  |  |  |  | 9.9 | [3.0, 18.0] | 12.7 | [6.0, 23.0] | 17.8 | [6.0, 27.0] |
| 11 |  |  |  |  |  | 10.8 | [3.0, 18.0] | 13.2 | [7.0, 21.0] | 20.6 | [6.0, 30.0] |
| 12 |  |  |  |  |  | 11.4 | [3.0, 22.0] | 28.1 | [20.0, 31.0] | 10.0 | [1.0, 27.0] |
| 13 |  |  |  |  |  | 12.4 | [6.0, 18.0] | 15.7 | [9.0, 24.0] | 26.9 | [13.0, 31.0] |
| 14 |  |  |  |  |  | 13.0 | [5.0, 21.0] | 13.1 | [5.0, 24.0] | 27.1 | [15.0, 31.0] |
| 15 |  |  |  |  |  | 12.9 | [4.0, 22.0] | 14.6 | [5.0, 24.0] | 17.5 | [6.0, 29.0] |
| 16 |  |  |  |  |  | 14.2 | [5.0, 21.0] | 14.8 | [6.0, 24.0] | 13.6 | [1.0, 29.0] |
| 17 |  |  |  |  |  | 18.0 | [9.0, 26.0] | 18.1 | [7.0, 29.0] | 15.9 | [3.0, 29.0] |
| 18 |  |  |  |  |  | 18.6 | [7.0, 28.0] | 16.0 | [4.0, 28.0] | 14.1 | [1.0, 29.0] |
| 19 |  |  |  |  |  | 19.1 | [12.0, 25.0] | 19.5 | [10.0, 28.0] | 15.2 | [1.0, 30.0] |
| 20 |  |  |  |  |  | 20.7 | [13.0, 27.0] | 19.4 | [9.0, 28.0] | 22.2 | [6.0, 31.0] |
| 21 |  |  |  |  |  | 20.8 | [10.0, 28.0] | 21.4 | [9.0, 29.0] | 5.0 | [1.0, 18.0] |
| 22 |  |  |  |  |  | 21.8 | [15.0, 26.0] | 19.1 | [8.0, 29.0] | 18.6 | [5.0, 29.0] |
| 23 |  |  |  |  |  | 21.9 | [14.0, 28.0] | 22.9 | [14.0, 31.0] | 20.2 | [4.0, 31.0] |
| 24 |  |  |  |  |  | 22.6 | [14.0, 28.0] | 19.7 | [6.0, 28.0] | 12.1 | [3.0, 25.0] |
| 25 |  |  |  |  |  | 23.2 | [17.0, 28.0] | 19.2 | [8.0, 28.0] | 16.6 | [2.0, 30.0] |
| 26 |  |  |  |  |  | 25.4 | [18.0, 31.0] | 23.4 | [13.0, 31.0] | 25.7 | [13.0, 31.0] |
| 27 |  |  |  |  |  | 27.4 | [22.0, 31.0] | 25.5 | [17.0, 31.0] | 15.3 | [2.0, 29.0] |
| 28 |  |  |  |  |  | 27.1 | [18.0, 31.0] | 29.1 | [19.0, 31.0] | 21.3 | [4.0, 31.0] |
| 29 |  |  |  |  |  | 27.8 | [21.0, 31.0] | 25.6 | [14.0, 30.0] | 13.6 | [2.0, 28.0] |
| 30 |  |  |  |  |  | 28.6 | [22.0, 31.0] | 26.8 | [16.0, 31.0] | 19.8 | [5.0, 30.0] |
| 31 |  |  |  |  |  | 29.9 | [25.0, 31.0] | 24.4 | [12.0, 30.0] | 7.3 | [2.0, 18.0] |

Table S5: Results of the bootstrap analysis (ibrier). The purple squares indicate which omics block(s) were included in the respective combinations. For the corresponding results obtained for rsf, bf, and ipflasso, see Table S4. The rows are ordered according to the positions obtained for rsf calculated using all datasets (without bootstrap). Low values correspond to a better and high values to a worse ranking. The columns “mean” and “ci” show the mean positions calculated using the 5,000 bootstrap samples and their 95% percentile confidence intervals. cnv: CNV, mirna: miRNA, mut: DNAseq, met: methylation, rna: mRNA.

| N0. | Combination | | | | | lasso | | prioritylasso | |
| --- | --- | --- | --- | --- | --- | --- | --- | --- | --- |
|  | mut | met | cnv | mirna | rna | mean | ci | mean | ci |
| 1 | 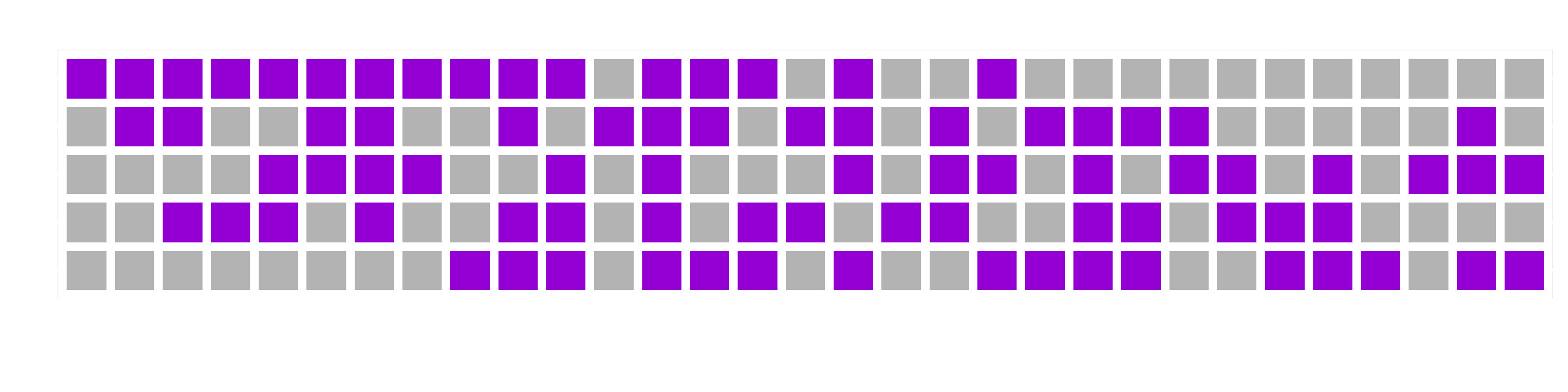 | | | | | 23.9 | [12.0, 31.0] | 2.9 | [1.0, 7.0] |
| 2 |  |  |  |  |  | 15.8 | [6.0, 27.0] | 9.3 | [7.0, 12.0] |
| 3 |  |  |  |  |  | 5.8 | [1.0, 16.0] | 29.7 | [27.0, 31.0] |
| 4 |  |  |  |  |  | 11.2 | [4.0, 24.0] | 22.7 | [18.0, 27.0] |
| 5 |  |  |  |  |  | 5.4 | [1.0, 15.0] | 28.5 | [25.0, 31.0] |
| 6 |  |  |  |  |  | 12.8 | [5.0, 24.0] | 14.5 | [13.0, 16.0] |
| 7 |  |  |  |  |  | 2.4 | [1.0, 6.0] | 23.9 | [18.0, 27.0] |
| 8 |  |  |  |  |  | 8.2 | [1.0, 19.0] | 12.8 | [10.0, 15.0] |
| 9 |  |  |  |  |  | 17.4 | [6.0, 28.0] | 21.1 | [16.0, 29.0] |
| 10 |  |  |  |  |  | 2.6 | [1.0, 7.0] | 25.9 | [19.0, 30.0] |
| 11 |  |  |  |  |  | 6.0 | [2.0, 13.0] | 23.9 | [19.0, 27.0] |
| 12 |  |  |  |  |  | 19.8 | [8.0, 31.0] | 2.9 | [1.0, 8.0] |
| 13 |  |  |  |  |  | 9.2 | [3.0, 20.0] | 30.1 | [27.0, 31.0] |
| 14 |  |  |  |  |  | 8.1 | [1.0, 18.0] | 27.7 | [23.0, 31.0] |
| 15 |  |  |  |  |  | 19.9 | [8.0, 30.0] | 17.9 | [15.0, 24.0] |
| 16 |  |  |  |  |  | 22.6 | [10.0, 31.0] | 18.5 | [16.0, 24.0] |
| 17 |  |  |  |  |  | 10.7 | [1.0, 25.0] | 25.3 | [20.0, 31.0] |
| 18 |  |  |  |  |  | 24.3 | [11.0, 31.0] | 2.8 | [1.0, 6.0] |
| 19 |  |  |  |  |  | 20.9 | [11.0, 30.0] | 16.8 | [14.0, 20.0] |
| 20 |  |  |  |  |  | 21.5 | [9.0, 31.0] | 22.2 | [17.0, 29.0] |
| 21 |  |  |  |  |  | 20.5 | [9.0, 29.0] | 12.3 | [9.0, 16.0] |
| 22 |  |  |  |  |  | 8.2 | [3.0, 16.0] | 20.1 | [17.0, 25.0] |
| 23 |  |  |  |  |  | 21.4 | [13.0, 30.0] | 21.3 | [18.0, 25.0] |
| 24 |  |  |  |  |  | 18.0 | [8.0, 28.0] | 11.0 | [8.0, 14.0] |
| 25 |  |  |  |  |  | 19.2 | [9.0, 30.0] | 5.1 | [2.0, 8.0] |
| 26 |  |  |  |  |  | 21.5 | [11.0, 31.0] | 7.0 | [4.0, 10.0] |
| 27 |  |  |  |  |  | 16.6 | [7.0, 28.0] | 8.9 | [5.0, 12.0] |
| 28 |  |  |  |  |  | 27.7 | [16.0, 31.0] | 7.8 | [4.0, 11.0] |
| 29 |  |  |  |  |  | 23.7 | [12.0, 31.0] | 2.8 | [1.0, 5.0] |
| 30 |  |  |  |  |  | 25.8 | [16.0, 31.0] | 13.9 | [12.0, 16.0] |
| 31 |  |  |  |  |  | 24.9 | [13.0, 31.0] | 6.1 | [3.0, 9.0] |

Table S7: Results of the bootstrap analysis (cindex). The purple squares indicate which omics block(s) were included in the respective combinations. For the corresponding results obtained for lasso and prioritylasso, see Table S8. The rows are ordered according to the positions obtained for rsf calculated using the data (without bootstrap). Low values correspond to a better and high values to a worse ranking. The columns “mean” and “ci” show the mean positions calculated using the 5,000 bootstrap samples and their 95% percentile confidence intervals. cnv: CNV, mirna: miRNA, mut: DNAseq, met: methylation, rna: mRNA.

| N0. | Combination | | | | | rsf | | bf | | ipflasso | |
| --- | --- | --- | --- | --- | --- | --- | --- | --- | --- | --- | --- |
|  | mut | met | cnv | mirna | rna | mean | ci | mean | ci | mean | ci |
| 1 | 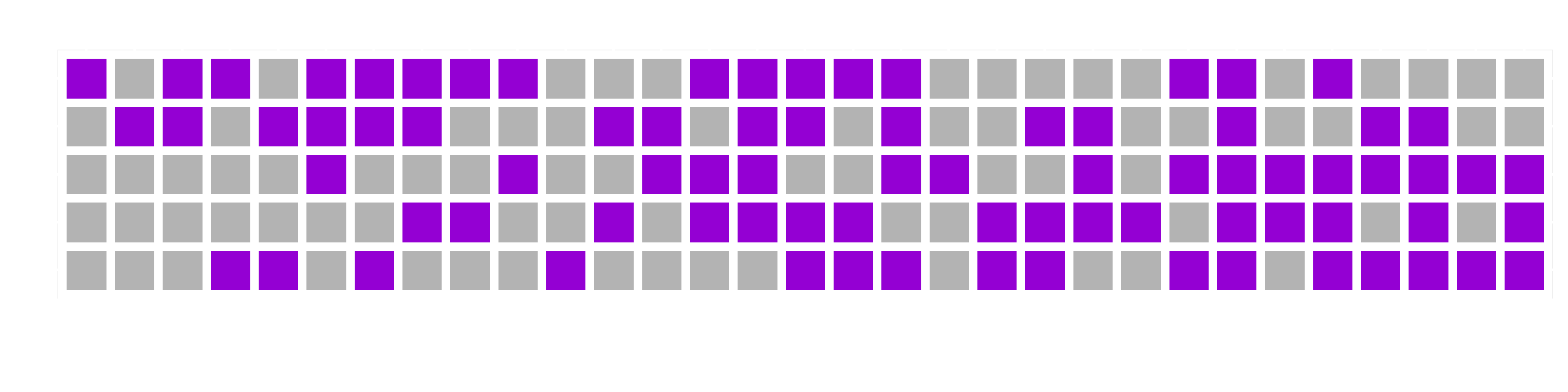 | | | | | 2.0 | [1.0, 5.0] | 1.8 | [1.0, 5.0] | 3.0 | [1.0, 9.0] |
| 2 |  |  |  |  |  | 2.6 | [1.0, 8.0] | 6.8 | [3.0, 17.0] | 8.1 | [1.0, 18.0] |
| 3 |  |  |  |  |  | 2.8 | [1.0, 6.0] | 1.6 | [1.0, 3.0] | 4.0 | [1.0, 12.0] |
| 4 |  |  |  |  |  | 5.7 | [1.0, 14.0] | 5.8 | [1.0, 15.0] | 12.8 | [4.0, 28.0] |
| 5 |  |  |  |  |  | 5.9 | [2.0, 14.0] | 11.8 | [4.0, 23.0] | 8.3 | [1.0, 20.0] |
| 6 |  |  |  |  |  | 8.5 | [4.0, 20.0] | 9.5 | [4.0, 19.0] | 4.0 | [1.0, 11.0] |
| 7 |  |  |  |  |  | 8.9 | [4.0, 20.0] | 7.5 | [3.0, 18.0] | 15.0 | [5.0, 30.0] |
| 8 |  |  |  |  |  | 8.8 | [3.0, 18.0] | 12.7 | [6.0, 21.0] | 23.3 | [7.0, 31.0] |
| 9 |  |  |  |  |  | 11.4 | [5.0, 22.0] | 10.6 | [4.0, 20.0] | 18.2 | [4.0, 27.0] |
| 10 |  |  |  |  |  | 12.3 | [5.0, 23.0] | 5.2 | [2.0, 12.0] | 9.5 | [5.0, 17.0] |
| 11 |  |  |  |  |  | 12.3 | [5.0, 22.0] | 10.1 | [3.0, 19.0] | 14.8 | [3.0, 23.0] |
| 12 |  |  |  |  |  | 13.1 | [5.0, 26.0] | 13.8 | [4.0, 26.0] | 13.1 | [2.0, 29.0] |
| 13 |  |  |  |  |  | 14.3 | [5.0, 26.0] | 12.0 | [4.0, 24.0] | 4.8 | [1.0, 13.0] |
| 14 |  |  |  |  |  | 14.4 | [7.0, 21.0] | 15.4 | [6.0, 23.0] | 24.2 | [13.0, 31.0] |
| 15 |  |  |  |  |  | 15.4 | [9.0, 22.0] | 20.5 | [12.0, 27.0] | 21.0 | [10.0, 30.0] |
| 16 |  |  |  |  |  | 15.8 | [6.0, 25.0] | 24.0 | [15.0, 30.0] | 22.3 | [14.0, 30.0] |
| 17 |  |  |  |  |  | 17.4 | [7.0, 26.0] | 24.9 | [13.0, 31.0] | 18.5 | [7.0, 29.0] |
| 18 |  |  |  |  |  | 17.6 | [8.0, 26.0] | 18.6 | [11.0, 27.0] | 17.9 | [8.0, 31.0] |
| 19 |  |  |  |  |  | 18.9 | [7.0, 29.0] | 13.8 | [5.0, 25.0] | 8.6 | [4.0, 20.0] |
| 20 |  |  |  |  |  | 18.7 | [8.0, 28.0] | 20.4 | [10.0, 29.0] | 26.7 | [16.0, 31.0] |
| 21 |  |  |  |  |  | 20.0 | [9.0, 28.0] | 19.0 | [9.0, 27.0] | 27.5 | [20.0, 31.0] |
| 22 |  |  |  |  |  | 21.1 | [12.0, 27.0] | 21.0 | [12.0, 28.0] | 24.1 | [12.0, 31.0] |
| 23 |  |  |  |  |  | 21.5 | [11.0, 30.0] | 10.3 | [4.0, 18.0] | 21.6 | [9.0, 30.0] |
| 24 |  |  |  |  |  | 21.9 | [10.0, 29.0] | 16.4 | [7.0, 27.0] | 11.2 | [1.0, 28.0] |
| 25 |  |  |  |  |  | 23.4 | [16.0, 28.0] | 26.5 | [18.0, 31.0] | 26.7 | [17.0, 31.0] |
| 26 |  |  |  |  |  | 24.5 | [16.0, 29.0] | 24.4 | [16.0, 30.0] | 21.0 | [7.0, 30.0] |
| 27 |  |  |  |  |  | 24.7 | [15.0, 29.0] | 25.8 | [18.0, 31.0] | 18.7 | [5.0, 28.0] |
| 28 |  |  |  |  |  | 23.9 | [10.0, 30.0] | 21.2 | [10.0, 30.0] | 7.5 | [1.0, 23.0] |
| 29 |  |  |  |  |  | 28.5 | [24.0, 30.0] | 29.0 | [24.0, 31.0] | 23.3 | [14.0, 31.0] |
| 30 |  |  |  |  |  | 29.1 | [22.0, 31.0] | 25.9 | [15.0, 31.0] | 11.5 | [4.0, 25.0] |
| 31 |  |  |  |  |  | 30.5 | [29.0, 31.0] | 29.7 | [26.0, 31.0] | 24.9 | [15.0, 31.0] |

Table S8: Results of the bootstrap analysis (cindex). The purple squares indicate which omics block(s) were included in the respective combinations. For the corresponding results obtained for rsf, bf, and ipflasso, see Table S7. The rows are ordered according to the positions obtained for rsf calculated using all datasets (without bootstrap). Low values correspond to a better and high values to a worse ranking. The columns “mean” and “ci” show the mean positions calculated using the 5,000 bootstrap samples and their 95% percentile confidence intervals. cnv: CNV, mirna: miRNA, mut: DNAseq, met: methylation, rna: mRNA.

| N0. | Combination | | | | | lasso | | prioritylasso | |
| --- | --- | --- | --- | --- | --- | --- | --- | --- | --- |
|  | mut | met | cnv | mirna | rna | mean | ci | mean | ci |
| 1 | 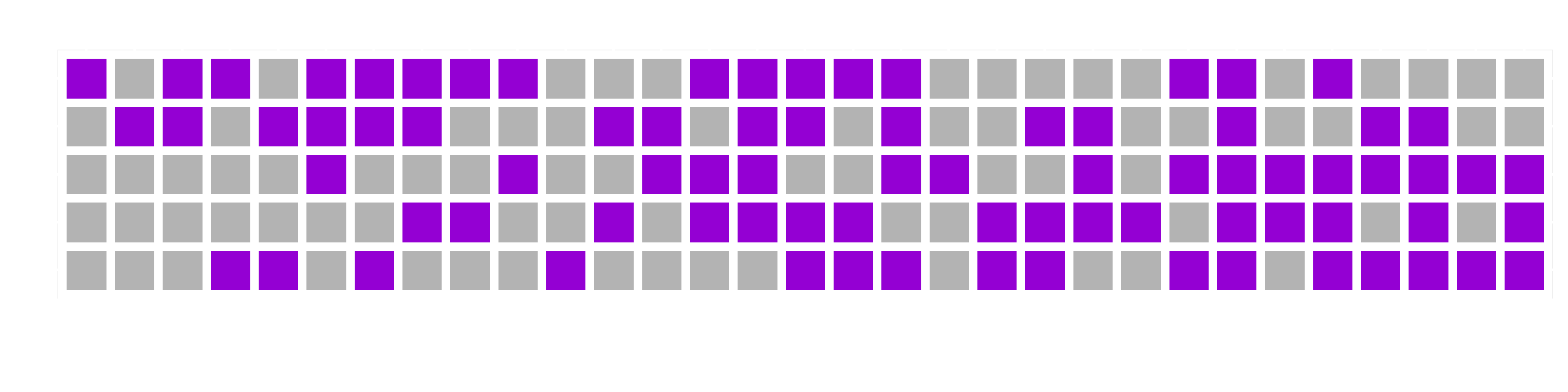 | | | | | 8.2 | [1.0, 24.0,] | 2.2 | [1.0, 6.0,] |
| 2 |  |  |  |  |  | 10.3 | [1.0, 24.0,] | 2.9 | [1.0, 8.0,] |
| 3 |  |  |  |  |  | 9.1 | [1.0, 26.0,] | 11.5 | [6.0, 13.0,] |
| 4 |  |  |  |  |  | 15.6 | [5.0, 28.0,] | 20.0 | [17.0, 25.0,] |
| 5 |  |  |  |  |  | 19.3 | [7.0, 28.0,] | 9.4 | [6.0, 13.0,] |
| 6 |  |  |  |  |  | 5.6 | [1.0, 18.0,] | 15.6 | [13.0, 18.0,] |
| 7 |  |  |  |  |  | 14.0 | [5.0, 26.0,] | 15.0 | [13.0, 18.0,] |
| 8 |  |  |  |  |  | 12.1 | [3.0, 24.0,] | 27.5 | [23.0, 31.0,] |
| 9 |  |  |  |  |  | 12.1 | [2.0, 25.0,] | 23.0 | [19.0, 27.0,] |
| 10 |  |  |  |  |  | 4.8 | [1.0, 16.0,] | 14.6 | [13.0, 17.0,] |
| 11 |  |  |  |  |  | 25.9 | [14.0, 31.0,] | 18.5 | [15.0, 22.0,] |
| 12 |  |  |  |  |  | 21.0 | [7.0, 30.0,] | 3.5 | [1.0, 8.0,] |
| 13 |  |  |  |  |  | 6.3 | [1.0, 16.0,] | 10.9 | [6.0, 14.0,] |
| 14 |  |  |  |  |  | 12.2 | [2.0, 27.0,] | 29.0 | [25.0, 31.0,] |
| 15 |  |  |  |  |  | 3.9 | [1.0, 12.0,] | 28.9 | [25.0, 31.0,] |
| 16 |  |  |  |  |  | 9.1 | [2.0, 18.0,] | 25.9 | [22.0, 30.0,] |
| 17 |  |  |  |  |  | 17.9 | [8.0, 27.0,] | 27.9 | [24.0, 31.0,] |
| 18 |  |  |  |  |  | 8.5 | [1.0, 20.0,] | 18.2 | [15.0, 22.0,] |
| 19 |  |  |  |  |  | 16.2 | [2.0, 29.0,] | 3.8 | [1.0, 8.0,] |
| 20 |  |  |  |  |  | 26.1 | [14.0, 31.0,] | 8.8 | [3.0, 13.0,] |
| 21 |  |  |  |  |  | 27.4 | [19.0, 31.0,] | 22.6 | [19.0, 27.0,] |
| 22 |  |  |  |  |  | 22.5 | [12.0, 30.0,] | 19.4 | [16.0, 22.0,] |
| 23 |  |  |  |  |  | 25.9 | [14.0, 31.0,] | 5.4 | [1.0, 10.0,] |
| 24 |  |  |  |  |  | 16.8 | [6.0, 28.0,] | 20.0 | [17.0, 24.0,] |
| 25 |  |  |  |  |  | 18.8 | [8.0, 29.0,] | 29.4 | [25.0, 31.0,] |
| 26 |  |  |  |  |  | 16.9 | [2.0, 30.0,] | 7.1 | [2.0, 13.0,] |
| 27 |  |  |  |  |  | 15.5 | [5.0, 25.0,] | 25.4 | [22.0, 28.0,] |
| 28 |  |  |  |  |  | 24.1 | [10.0, 31.0,] | 9.7 | [6.0, 13.0,] |
| 29 |  |  |  |  |  | 18.1 | [6.0, 27.0,] | 23.9 | [21.0, 29.0,] |
| 30 |  |  |  |  |  | 25.7 | [12.0, 31.0,] | 5.9 | [2.0, 10.0,] |
| 31 |  |  |  |  |  | 26.2 | [18.0, 31.0,] | 10.3 | [5.0, 14.0,] |

**Dataset specific ranks of each combination of prediction method and blocks (cindex)**


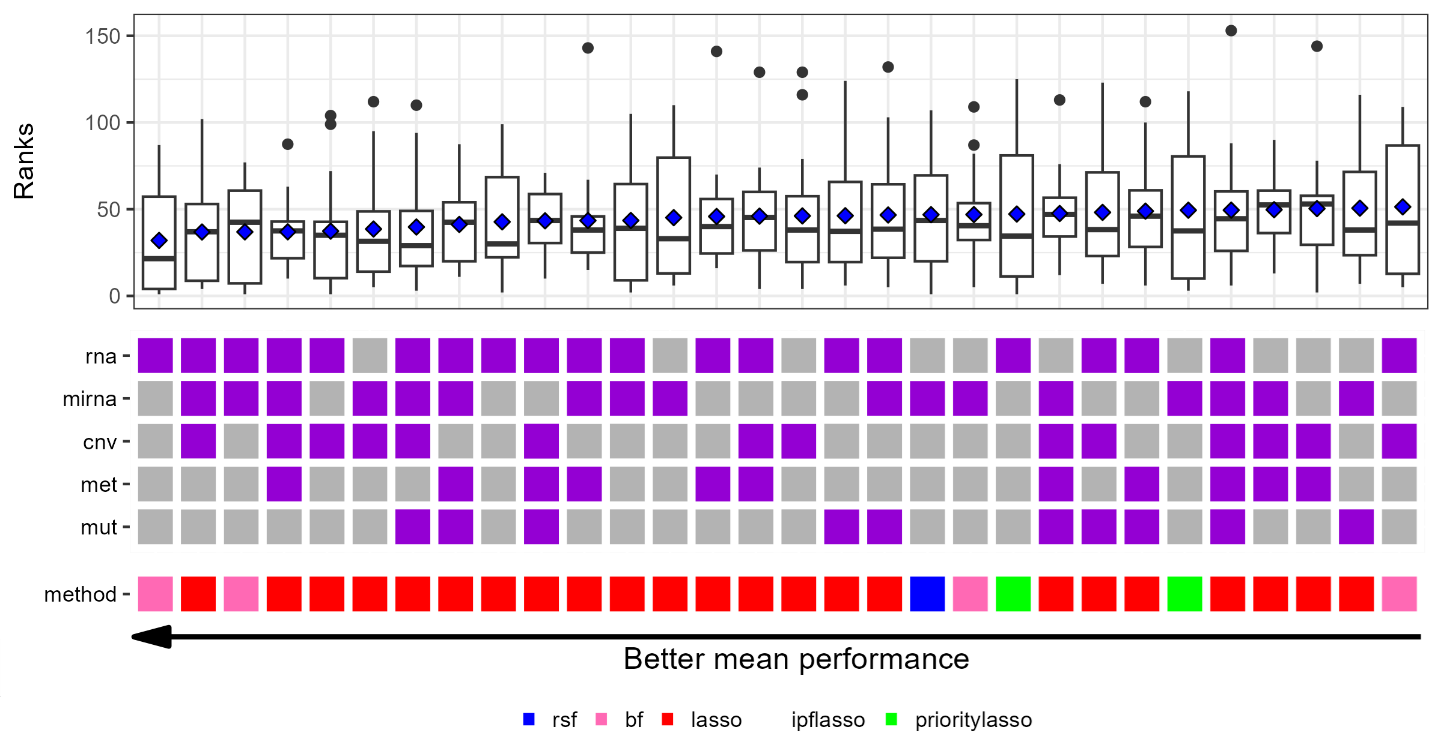


Figure S12: Dataset specific ranks of each combination of prediction method and blocks (cindex). The ranks of each combination among all 155 combinations of prediction methods and blocks are shown. The purple squares indicate which omics block(s) were included in the respective combinations. The values shown by the boxplots are the ranks achieved across all 14 datasets, where the blue diamonds represent the means of the ranks. Smaller ranks indicate a better predictive performance. The combinations are sorted in increasing order according to the mean ranks across the datasets, which is why the combinations further to the left tend to perform better. For reasons of clarity, only the 30 combinations with the smallest positions are shown. cnv: CNV, mirna: miRNA, mut: DNAseq, met: methylation, rna: mRNA.

**Dataset specific mean cross-validated performance measure values of each combination of prediction method and blocks**


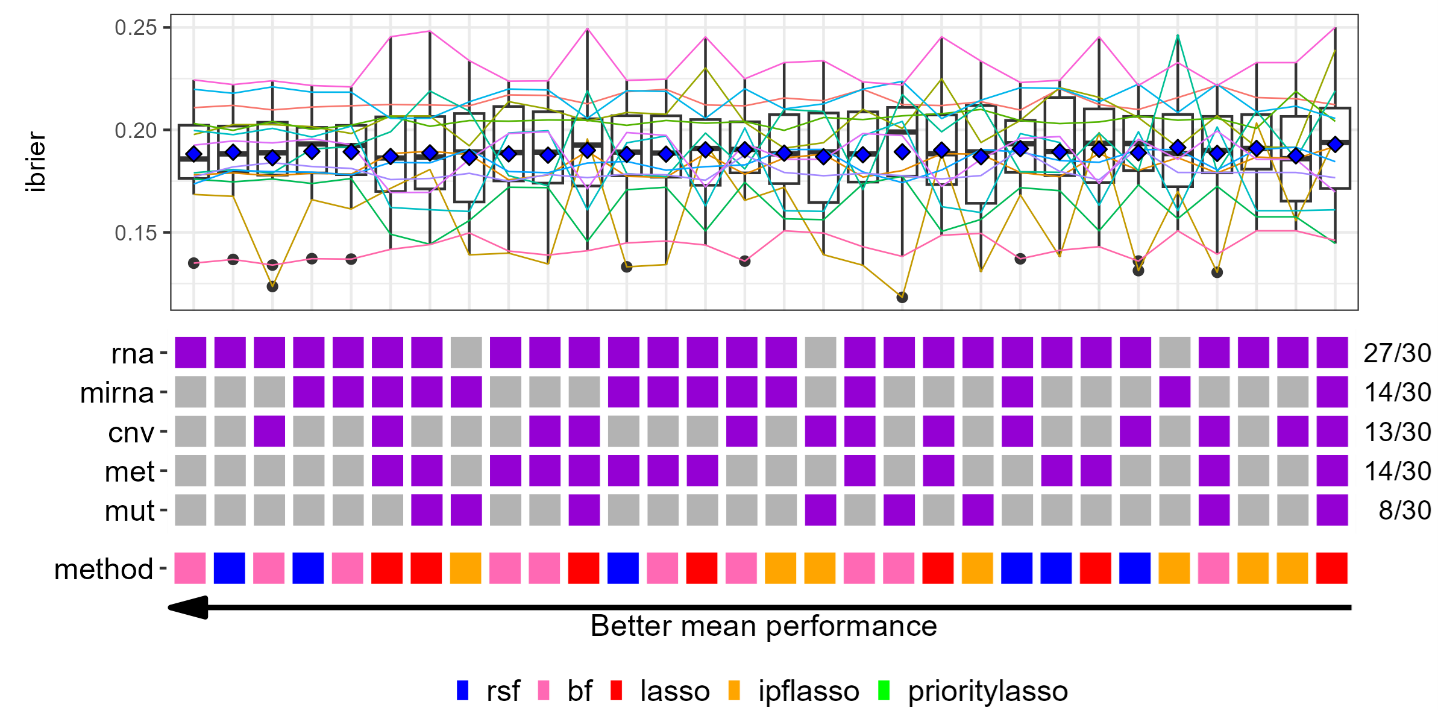


Figure S13: Dataset specific mean cross-validated ibrier values of each combination of prediction method and blocks. The purple squares indicate which omics block(s) were included in the respective combinations. The values shown by the boxplots are the ibrier values achieved across all 14 datasets, where the blue diamonds represent the means of the values. The combinations are sorted in increasing order according to the mean ranks across the datasets, which is why the combinations further to the left tend to perform better. For reasons of clarity, only the 30 combinations with the smallest mean ranks are shown. cnv: CNV, mirna: miRNA, mut: DNAseq, met: methylation, rna: mRNA.


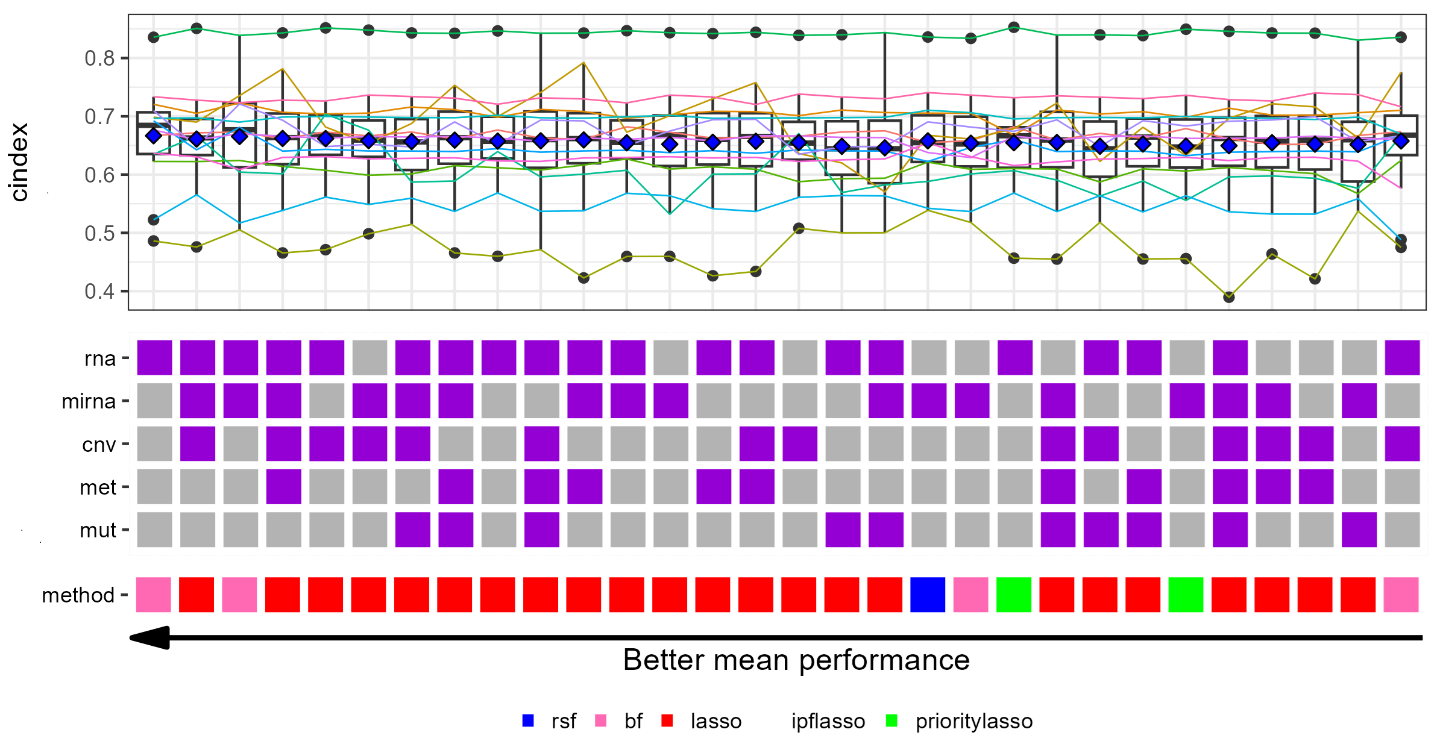


Figure S14: Dataset specific mean cross-validated cindex values of each combination of prediction method and blocks. The purple squares indicate which omics block(s) were included in the respective combinations. The values shown by the boxplots are the cindex values achieved across all 14 datasets, where the blue diamonds represent the means of the values. The combinations are sorted in increasing order according to the mean ranks across the datasets, which is why the combinations further to the left tend to perform better. For reasons of clarity, only the 30 combinations with the smallest mean ranks are shown. cnv: CNV, mirna: miRNA, mut: DNAseq, met: methylation, rna: mRNA.

**Statistical testing for overrepresentation of mRNA in the 30 best combinations**

We examine the chance of mRNA occurring in the top 30 combinations as frequently or more frequently as it actually occurred. We assume the null hypothesis that all 155 combinations are equally important in prediction and, for simplicity, that the combinations are independent. In this case, the number of combinations featuring mRNA within the top 30 is hypergeometrically distributed. Thus, using the hypergeometric distribution, we can calculate the probability of mRNA appearing at least 27 times under the null hypothesis - the frequency we observed. This probability is calculated to be $1.17\times{10}^{-6}$. The aforementioned value constitutes a p-value as it represents the probability of obtaining a result as extreme as the observed one, assuming the null hypothesis is true. It is important to note that this p-value requires adjustment for multiple testing. This adjustment is necessary because the test was conducted post hoc, following the observation that mRNA was the most common block in the best combinations. Considering that any of the five blocks could have been the most frequent, an adjustment for the data-driven decision to test mRNA is warranted. This adjustment can be performed using the Bonferroni correction, which in this case consists of multiplying the original p-value by five. Consequently, the adjusted p-value is $5.9\times{10}^{-6}$, thereby maintaining the high significance of the result even after adjusting for multiple testing.

**Statistical testing procedures regarding the best-performing combinations of prediction methods and blocks per dataset (Table 3 in the main paper)**

**Tests regarding the numbers of blocks in the best-performing combinations**

In the “Best-performing combinations of prediction methods and blocks per dataset” section of the main paper, we observed that for the great majority of datasets, the best performance was achieved using only up to two blocks. We now evaluate the likelihood of these findings. Under the null hypothesis that each combination has an equal chance of being the best-performing, the number of datasets where the best-performing combination according to the ibrier or cindex contains only one or two blocks follows a binomial distribution. Therefore, the binomial distribution is used to calculate the probability that this number is at least equal to the observed number. The calculated probabilities, which represent p-values, are 0.022 for the ibrier and $3.9\times{10}^{-5}$ for the cindex.

**Tests for overrepresentation of mRNA and miRNA in the best-performing combinations pre dataset**

As outlined in the “Best-performing combinations of prediction methods and blocks per dataset” section of the paper, we statistically evaluated whether mRNA and miRNA occurred statistically significantly more frequently in the best-performing combinations for each dataset than would be expected by chance. Here, our analysis conditioned on the observed numbers of blocks in the best-performing combinations. This consideration was crucial, as the occurrence probabilities of the blocks depend on these numbers. For instance, a block is thrice as likely to occur in a combination comprising three blocks compared to a combination with only one block.. Hereinafter, for succinctness, “combination(s)” will refer to “best-performing combination(s)”.

The null hypothesis posits that each block is equally probable to be present in the combinations. The p-value of the test for mRNA (miRNA) is calculated as the probability that, under the null hypothesis, the number of combinations including mRNA (miRNA) equals or exceeds the observed frequency, given the observed number of blocks in the combinations. The event of whether or not a specific dataset's combination contains mRNA (miRNA) follows a Bernoulli distribution with a success probability of m/5, where m is the number of blocks in the combination. For example, a combination with one block has a success probability of 1/5, whereas a combination with four blocks has a success probability of 4/5. Thus, under the null hypothesis, the number of combinations that contain mRNA (miRNA) is a sum of independent Bernoulli-distributed variables with differing probability parameters. Such sums are known to be Poisson binomially distribution because the Poisson binomial distribution is a generalization of the binomial distribution to cases, where the probabilities of the events differ from each other.

Therefore, we can use the Poisson binomial distribution to calculate the p-values of the tests for mRNA and miRNA. These are 0.134 and 0.516 for the ibrier, and 0.006 and 0.211 for the cindex, respectively. Similar to in the “Ranking of the predictive performance of all prediction methods on all block combinations” section of the paper, these p-values necessitate adjustment due to the data-driven focus on mRNA and miRNA. Since three of these p-values exceed the 0.05 threshold for statistical significance prior to adjustment for multiple testing, it is only necessary to adjust the p-value 0.006. This correction is again made using the Bonferroni correction, which requires multiplying by five. The resulting adjusted p-value is 0.031, thereby still representing a statistically significant result. Consequently, we can reject the null hypothesis only for the occurrence of mRNA, and solely for the cindex.

**Detailed description of the analysis of the dataset-specific rankings of combinations involving only mRNA, miRNA or methylation data**

In the following, we always consider the combinations of block combinations and prediction methods, that is, a total of 155 combinations (31 block combinations × 5 prediction methods). For the ibrier, the best-performing combination involving only mRNA or miRNA (excluding other blocks, i.e., solely mRNA, miRNA, or a combination of both) ranked in the top 10% for 9 out of 14 datasets and in the top 30% for the remaining 5 datasets (namely COAD, LGG, LIHC, LUSC, and SARC). Furthermore, for these latter 5 datasets, the best mRNA or miRNA combination was outperformed by the best out of the 5 combinations using all 5 blocks (one for each prediction method). Remarkably, for all of these 5 datasets, the optimal combination (refer to Table 3 in the main paper) included methylation data, even though methylation data was present in the best combination for only 6 datasets in total. This observation led us to speculate that methylation data played a crucial role in these results, a suggestion supported by the fact that for all 14 datasets, the best combination including mRNA, miRNA, or methylation data outperformed the best combination using all available blocks.

Similarly, for the cindex, the best mRNA or miRNA combination was in the top 10% for 11 out of 14 datasets and in the top 30% for all datasets. For all but one dataset, this combination outperformed the best combination using all available blocks. Interestingly, the exception, the SARC dataset, was the only one where the best overall combination included methylation data (see Table 3 in the main paper).

The inclusion of methylation data substantially enhanced the ranking of the best combinations (consisting of mRNA, miRNA, or methylation data) among all combinations for both performance metrics, particularly for the ibrier, where after adding methylation data, the best combinations for all datasets were in the top 10% of all combinations.

# References

[1] Q. Zhao, X. Shi, Y. Xie, J. Huang, B. Shia, and S. Ma, “Combining multidimensional genomic measurements for predicting cancer prognosis: observations from TCGA,” *Brief. Bioinform.*, vol. 16, no. 2, pp. 291–303, 2015.

[2] H. Gómez-Rueda, E. Mart\’\inez-Ledesma, A. Mart\’\inez-Torteya, R. Palacios-Corona, and V. Trevino, “Integration and comparison of different genomic data for outcome prediction in cancer,” *BioData Min.*, vol. 8, pp. 1–12, 2015.

[3] R. Hornung and M. N. Wright, “Block Forests: random forests for blocks of clinical and omics covariate data,” *BMC Bioinformatics*, vol. 20, no. 1, pp. 1–17, 2019.

[4] M. Herrmann, P. Probst, R. Hornung, V. Jurinovic, and A.-L. Boulesteix, “Large-scale benchmark study of survival prediction methods using multi-omics data,” *Brief. Bioinform.*, vol. 22, no. 3, p. bbaa167, 2021.

[5] D. Wissel, D. Rowson, and V. Boeva, “Systematic comparison of multi-omics survival models reveals a widespread lack of noise resistance,” *Cell Reports Methods*, vol. 3, no. 4, 2023.

[6] L. A. Vale-Silva and K. Rohr, “Long-term cancer survival prediction using multimodal deep learning,” *Sci. Rep.*, vol. 11, no. 1, p. 13505, 2021.

[7] A. Osipov *et al.*, “The Molecular Twin artificial-intelligence platform integrates multi-omic data to predict outcomes for pancreatic adenocarcinoma patients,” *Nat. cancer*, vol. 5, no. 2, pp. 299–314, 2024.

[8] B. Efron, “Bootstrap Methods: Another Look at the Jackknife,” *Ann. Stat.*, vol. 7, no. 1, pp. 1–26, 1979.

[9] R. Stine, “An introduction to bootstrap methods: Examples and ideas,” *Sociol. Methods Res.*, vol. 18, no. 2–3, pp. 243–291, 1989.
